# Inhibition of SUR1-TRPM4 attenuates astrocyte swelling and reactivity under oxygen-glucose deprivation/reoxygenation

**DOI:** 10.1371/journal.pone.0352151

**Published:** 2026-06-24

**Authors:** Yuanyuan Zhao, Narawut Pakaprot, Rujapope Sutiwisesak, Unchalee Vattarakorn, Sompol Tapechum

**Affiliations:** Department of Physiology, Faculty of Medicine Siriraj Hospital, Mahidol University, Bangkok, Thailand; Albany Medical College, UNITED STATES OF AMERICA

## Abstract

Astrocytes play essential roles in maintaining brain homeostasis but undergo pathological alterations following ischemia-reperfusion (I/R) injury. Astrocyte swelling and reactive changes are associated with secondary injury processes, including cerebral edema and neuroinflammatory responses. In vitro injury models have demonstrated cellular swelling and stress-induced alterations in astrocytes. The sulfonylurea receptor 1-transient receptor potential melastatin 4 (SUR1-TRPM4) channel has been implicated in astrocyte swelling; however, its potential association with downstream stress-related signaling responses remains incompletely understood. In this study, CTX-TNA2 cells, a rat astrocyte cell line, were subjected to oxygen-glucose deprivation/reoxygenation (OGD/R) to model I/R injury. Cellular swelling, astrocyte reactivity, and cell viability were evaluated together with indicators of oxidative stress, endoplasmic reticulum stress, and STAT3-associated signaling responses. OGD/R induced cell swelling, increased GFAP expression, and reduced cell viability, accompanied by enhanced NOX4-related oxidative stress, activation of PERK-mediated endoplasmic reticulum stress signaling, and STAT3 activation. Pharmacological inhibition of SUR1-TRPM4 attenuated astrocyte swelling and those signaling cascades under OGD/R conditions, accompanied by reduced GFAP expression and improved cell viability. Modulation of individual stress pathways partially attenuated GFAP expression under OGD/R conditions. These findings suggest that SUR1-TRPM4 contributes not only to astrocytic swelling but also to astrocyte reactivity and cellular stress responses under in vitro ischemia-like conditions.

## Introduction

Ischemic stroke is a major cause of mortality and long-term neurological impairment worldwide [1]. Although reperfusion remains the cornerstone of acute treatment, it paradoxically initiates ischemia-reperfusion (I/R) injury, a complex pathological process involving ionic imbalance [2], oxidative stress [3], endoplasmic reticulum (ER) stress [4], and inflammatory responses [5,6]. These secondary injury mechanisms exacerbate cellular dysfunction and contribute substantially to brain tissue damage [7], highlighting the importance of identifying upstream molecular regulators that coordinate post-ischemic stress responses.

As the most abundant glial cell population in the central nervous system, astrocytes play a central role in maintaining brain homeostasis and act as key regulators of cellular responses to ischemic stress [8,9]. Under physiological conditions, astrocytes regulate extracellular ionic homeostasis, neurotransmitter clearance and recycling, metabolic coupling with neurons, and immune signaling within the neural microenvironment [10]. Following cerebral ischemia, astrocytes undergo reactive changes characterized by cellular hypertrophy, upregulation of intermediate filament proteins such as glial fibrillary acidic protein (GFAP), and transcriptional reprogramming [11,12]. Although astrocyte reactivation is generally considered an early adaptive response to ischemic insult, excessive reactivation and swelling contribute to cerebral edema and generate a microenvironment that is unfavorable for neural recovery [13,14]. Notably, astrocyte swelling, driven by ionic dysregulation and osmotic imbalance under ischemic conditions, is associated with subsequent cellular stress responses such as oxidative stress and inflammation [15,16].

Cerebral edema is a major pathological feature of I/R injury, reflecting abnormal water and ion accumulation within brain tissue [17]. At the cellular level, this process is largely driven by astrocyte swelling and is closely associated with dysregulated ion transport, pointing to ion imbalance as a key mechanistic trigger [18]. The sulfonylurea receptor 1-transient receptor potential melastatin 4 (SUR1-TRPM4) channel is increasingly recognized as a critical molecular regulator of ischemia-induced astrocyte swelling [19,20]. Importantly, this channel is a de novo-induced non-selective cation channel that is minimally expressed under physiological conditions but markedly upregulated following central nervous system injury [21,22]. Upon ischemic insult, transcriptional induction of SUR1 and its assembly with TRPM4 give rise to a functional channel complex that mediates sustained sodium influx and membrane depolarization. This pathological ionic dysregulation facilitates osmotic water accumulation, leading to astrocyte swelling and blood-brain barrier dysfunction [23,24]. Pharmacologically, SUR1-TRPM4 activity can be inhibited by glibenclamide, a widely used sulfonylurea compound. Pharmacological inhibition of SUR1-TRPM4 with glibenclamide (GLI) has been shown to reduce cerebral edema and tissue damage in experimental models of ischemic stroke [23,25–27]. However, whether SUR1-TRPM4 acts solely as an ionic effector or additionally serves as an upstream regulator of intracellular stress signaling in astrocytes under ischemic conditions remains unclear.

Oxidative stress, ER stress, and inflammatory signaling constitute a tightly coupled intracellular stress network that governs astrocyte fate and reactivity during I/R injury [13,28,29]. In astrocytes, NADPH oxidase 4 (NOX4) is a major enzymatic source of reactive oxygen species (ROS), whereas ER stress activates the unfolded protein response (UPR), predominantly through PERK-dependent signaling pathways [30]. These stress responses are not independent but are functionally interrelated: ROS exacerbates ER stress and inflammatory signaling, ER stress promotes inflammatory cytokine production via UPR activation, and inflammatory pathways further amplify oxidative and ER stress, forming a self-reinforcing signaling loop [28,31–33]. Signal transducer and activator of transcription 3 (STAT3) acts as a central transcriptional integrator within this network, translating oxidative and ER stress signals into inflammatory gene programs and reactive astrocyte phenotypes [30,34]. Genetic and pharmacological studies have demonstrated that astrocyte-specific STAT3 activation is required for the development and maintenance of reactive astrocyte states, whereas its inhibition attenuates astrocyte reactivity and limits tissue damage [35–37]. Previous studies have established important roles for SUR1-TRPM4 channel in ischemia-associated swelling responses and edema-related injury mechanisms in experimental central nervous system injury models [20,38,39]. Despite substantial evidence linking SUR1-TRPM4 channel to ionic dysregulation and edema-associated cellular responses, whether this channel acts as an upstream regulator of oxidative stress, ER stress, and inflammatory signaling in astrocyte remains unclear. In particular, the relationship between SUR1-TRPM4-associated swelling responses and downstream stress-related or paracrine signaling mechanisms under OGD/R conditions has not been fully characterized. Clarifying these interactions may provide mechanistic insight into how ischemia- associated ionic disturbances contribute to astrocytes reactivity in vitro.

Here, we investigated SUR1-TRPM4-associated swelling and astrocyte reactivity in CTX-TNA2 cells following oxygen-glucose deprivation/reoxygenation (OGD/R), an in vitro model of I/R injury. We further examined whether SUR1-TRPM4 channel is associated with NOX4-related oxidative stress, PERK- associated ER stress, and STAT3-related inflammatory responses under OGD/R conditions. This study provides insight into potential links between SUR1-TRPM4-associated ionic dysregulation and downstream signaling cascades in an in vitro astrocyte injury model.

## Materials and methods

### Cell culture

The CTX-TNA2 cells (ATCC CRL-2006, USA), originally derived from neonatal rat cortical astrocytes, was obtained from the American Type Culture Collection. Cells were cultured in Dulbecco’s modified Eagle’s medium (DMEM; Gibco, USA) supplemented with 10% fetal bovine serum (FBS; Sigma-Aldrich, USA) and 1% penicillin-streptomycin (Thermo Fisher Scientific, USA). Cells were maintained at 37 °C in a humidified incubator with 5% CO_2_. The culture medium was replaced every 2–3 days, and experiments were performed when cells reached approximately 60–70% confluence.

### Oxygen-glucose deprivation/reoxygenation

CTX-TNA2 cells were subjected to OGD/R using a previously described in vitro protocol with minor modifications [40]. Briefly, cells were incubated in glucose-free DMEM and placed in a 2 L sterilized polytetrafluoroethylene (PTFE) gas-sampling bag filled with 95% N_2_ and 5% CO_2_, together with an anaerobic pack and an oxygen indicator (Mitsubishi Gas Chemical, Tokyo, Japan). The sealed bag containing the culture plates was then maintained at 37 °C for 6 h to induce OGD. After OGD, cultures were washed once with 1 × PBS and the medium was replaced with complete culture medium with or without pharmacological treatments. Cells were then returned to normoxic conditions (37 °C, 5% CO_2_) for 24 h of reoxygenation, except for cell volume analysis, which was performed 12 h after reoxygenation. Control cells were maintained in complete culture medium under normoxic conditions throughout the experiment. At the end of the designated reoxygenation period, cells were collected for subsequent biochemical and imaging analyses.

### Drugs preparation

Glibenclamide (Sigma-Aldrich, Singapore) was dissolved in dimethyl sulfoxide (DMSO) and diluted to a final concentration of 40 µM, with the final DMSO concentration not exceeding 0.1% (v/v). GSK2606414 (GSK), a selective PERK inhibitor (Cayman Chemical, USA), was dissolved in DMSO and diluted to a final concentration of 5 µM. Setanaxib (GKT137831, GKT), a dual NADPH oxidase 1/4 (NOX1/4) inhibitor (MedChemExpress, USA), was dissolved in DMSO and diluted to a final concentration of 10 µM. Mannitol (Sigma-Aldrich, Singapore) was dissolved directly in culture medium to generate a hypertonic culture medium (final concentration 60 mM; osmolarity ≈ 390 mOsm/L). All solutions were freshly prepared before use. Treatments were initiated immediately after the OGD, and cells were incubated under standard culture conditions for 12 or 24 h before sample collection.

### Astrocyte-conditioned medium preparation

Astrocyte-conditioned medium (ACM) was collected from CTX-TNA2 cells under normoxic condition or following 6 h of OGD with or without 40 μM glibenclamide (GLI), followed by 24 h of reoxygenation. To minimize experimental variability in ACM composition, donor cells were cultured under identical experimental conditions using similar passage numbers and identical seeding densities across all experiments. ACM collection was performed using the same preparation procedure across all experimental replicates. Culture supernatants were collected and centrifuged at 1000 × g for 10 min at 4 °C to remove cellular debris. Equal volumes of centrifuged ACM from each experimental group were transferred to recipient CTX-TNA2 cells by replacing the original culture medium, followed by incubation for 24 h under normoxic conditions prior to subsequent analyses.

### Assessment of cell viability

Cell viability was assessed using the PrestoBlue™ Cell Viability Reagent (Thermo Fisher Scientific, USA). CTX-TNA2 cells were seeded in 96-well plates and exposed to the indicated experimental conditions. Separate plates were used for normoxic and OGD/R experiments, and each condition was analyzed using three technical replicate wells per experiment. At the end of treatment, the culture medium was replaced with 100 µL of 10% (v/v) PrestoBlue™ solution prepared in complete DMEM. Plates were incubated at 37 °C for 1 h, protected from light. Absorbance was measured at 570 nm with a reference wavelength of 600 nm using a microplate reader (Agilent Technologies, USA). Background signals were corrected using wells containing reagent without cells. The absorbance at 570 nm was corrected by subtracting the reference absorbance at 600 nm for optical imperfections, and background values from cell-free wells were further subtracted. Cell viability was expressed as a percentage relative to the control group.

### Assessment of cell volume

Cell volume was quantified using the Tomocube HT-2H system (Tomocube Inc., Korea), which employs refractive index (RI) tomography and quantitative phase imaging for label-free three-dimensional reconstruction of living cells. CTX-TNA2 cells were cultured in TomoDish imaging dishes (Tomocube Inc., Korea; Cat. No. 901002−02) and subjected to the indicated experimental conditions. Each dish was divided into four regions, and representative images were acquired from each region. Image processing and parameter extraction were performed using the HTX processing server (Tomocube Inc., Korea), which automatically generated single-cell measurements, including cell volume, mean RI, and intracellular concentration. In the Tomocube analysis system, intracellular concentration represents an RI-derived parameter reflecting the relative intracellular dry mass concentration within individual cells.

### ROS assay

Intracellular ROS production was measured using the Total ROS Assay Kit (Thermo Fisher Scientific, USA; Cat. No. 88-5930-74). CTX-TNA2 cells were seeded in 96-well plates and subjected to the indicated experimental conditions. Separate 96-well plates were used for normoxic and OGD/R experiments. Each condition was analyzed using three technical replicate wells per experiment.After treatment, cells were washed once with 1 × PBS and incubated with the ROS detection reagent at 37 °C for 1 h protected from light according to the manufacturer’s instructions. Cells were subsequently washed once with PBS to reduce extracellular background fluorescence, and fluorescence intensity was measured using a microplate reader with an excitation wavelength of 488 nm and an emission wavelength of 520 nm. Background fluorescence was corrected using wells containing detection reagent without cells. ROS levels were expressed as relative fluorescence units (RFU) normalized to the control group.

### Immunofluorescent staining

Immunofluorescence staining was performed as previously described [41], with minor modifications. Briefly, CTX-TNA2 cells cultured on glass coverslips were fixed with 4% paraformaldehyde for 15 min (Sigma-Aldrich, USA), permeabilized with 0.2% Triton X-100 for 10 min (Sigma-Aldrich, USA) and blocked with 2% bovine serum albumin (BSA; Sigma-Aldrich, USA) for 1 h at room temperature. Cells were incubated with primary antibodies against GFAP (rabbit, 1:100; Cell Signaling Technology, USA; Cat. No. 12389, or mouse, 1:100; Thermo Fisher Scientific, USA; Cat. No. 14989282), NOX4 (rabbit, 1:100; Thermo Fisher Scientific, USA; Cat. No. PA5–95083), p-PERK (rabbit, 1:100; Thermo Fisher Scientific, USA; Cat. No. PA5–40294), and phospho-STAT3 (p-STAT3; rabbit, 1:100; Cell Signaling Technology, USA; Cat. No. 9145) for 2 h at room temperature. After washing, cells were incubated with Alexa Fluor 488-conjugated goat anti-rabbit IgG (H + L) (1:1000; Thermo Fisher Scientific, USA; Cat. No. A11008) or Alexa Fluor 555-conjugated goat anti-mouse IgG (H + L) (1:1000; Thermo Fisher Scientific, USA; Cat. No. A21422) for 1 h at room temperature. Nuclei were counterstained with DAPI (Thermo Fisher Scientific, USA). Fluorescence images were acquired using an Axio Imager.M2 fluorescence microscope (Carl Zeiss, Jena, Germany) equipped with a 40 × objective lens. Fluorescence intensity was quantified using ImageJ software (NIH, USA).

### Western blot analysis

Western blot analysis was performed according to standard procedures with minor modifications. Total protein was extracted from cultured cells using RIPA lysis buffer supplemented with protease and phosphatase inhibitors. Protein concentrations were determined using the Bradford assay (Bio-Rad, USA; Cat. No. 5000006). Equal amounts of protein (30 µg) were separated by SDS-PAGE using 6–10% gels, depending on protein molecular weight, and subsequently transferred onto polyvinylidene difluoride (PVDF) membranes. Membranes were blocked with 5% nonfat milk or 5% BSA in 1 × TBST depending on the antibody and incubated overnight at 4 °C with the following primary antibodies: GFAP (rabbit, 1:1000; Cell Signaling Technology, USA; Cat. No. 12389), NOX4 (rabbit, 1:1000; Thermo Fisher Scientific, USA; Cat. No. PA5–95083), STAT3 (mouse, 1:1000; Cell Signaling Technology, USA; Cat. No. 9139), phospho-STAT3 (p-STAT3; rabbit, 1:1000; Cell Signaling Technology, USA; Cat. No. 9145), and β-actin (mouse, 1:2000; Cell Signaling Technology, USA; Cat. No. 4970S). After washing with 1 × TBST, membranes were incubated with the appropriate HRP-conjugated secondary antibodies: anti-mouse IgG-HRP (horse, 1:3000; Cell Signaling Technology, USA; Cat. No. 7076) or anti-rabbit IgG-HRP (goat, 1:4000; Thermo Fisher Scientific, USA; Cat. No. 656120). Immunoreactive bands were detected using enhanced chemiluminescence (ECL) reagents (Thermo Fisher Scientific, USA) and visualized with an ImageQuant LAS 4010 biomolecular imager (GE Healthcare, USA). Band intensities were quantified using ImageJ software (NIH, USA) and normalized to β-actin. β-actin loading controls and the corresponding target proteins were obtained from the same membrane, although different exposure times were used to improve band visualization.

### Statistical analysis

Data are presented as mean ± SD unless otherwise indicated. Single-cell biophysical parameters are presented as median with interquartile range (IQR), with minimum and maximum values indicated. For normalized datasets in which control values were set to 100 or 1, statistical significance against the normalized control value was determined using one-sample t-tests with Bonferroni correction. Comparisons among treatment groups were analyzed using ordinary one-way ANOVA followed by Tukey’s post hoc test where appropriate. A value of p ≤ 0.05 was considered statistically significant. All statistical analyses were performed using GraphPad Prism (version 9.0; GraphPad Software, USA). All experiments were repeated independently at least three times.

## Results

### Glibenclamide improved cell viability following OGD/R

**Fig 1:**
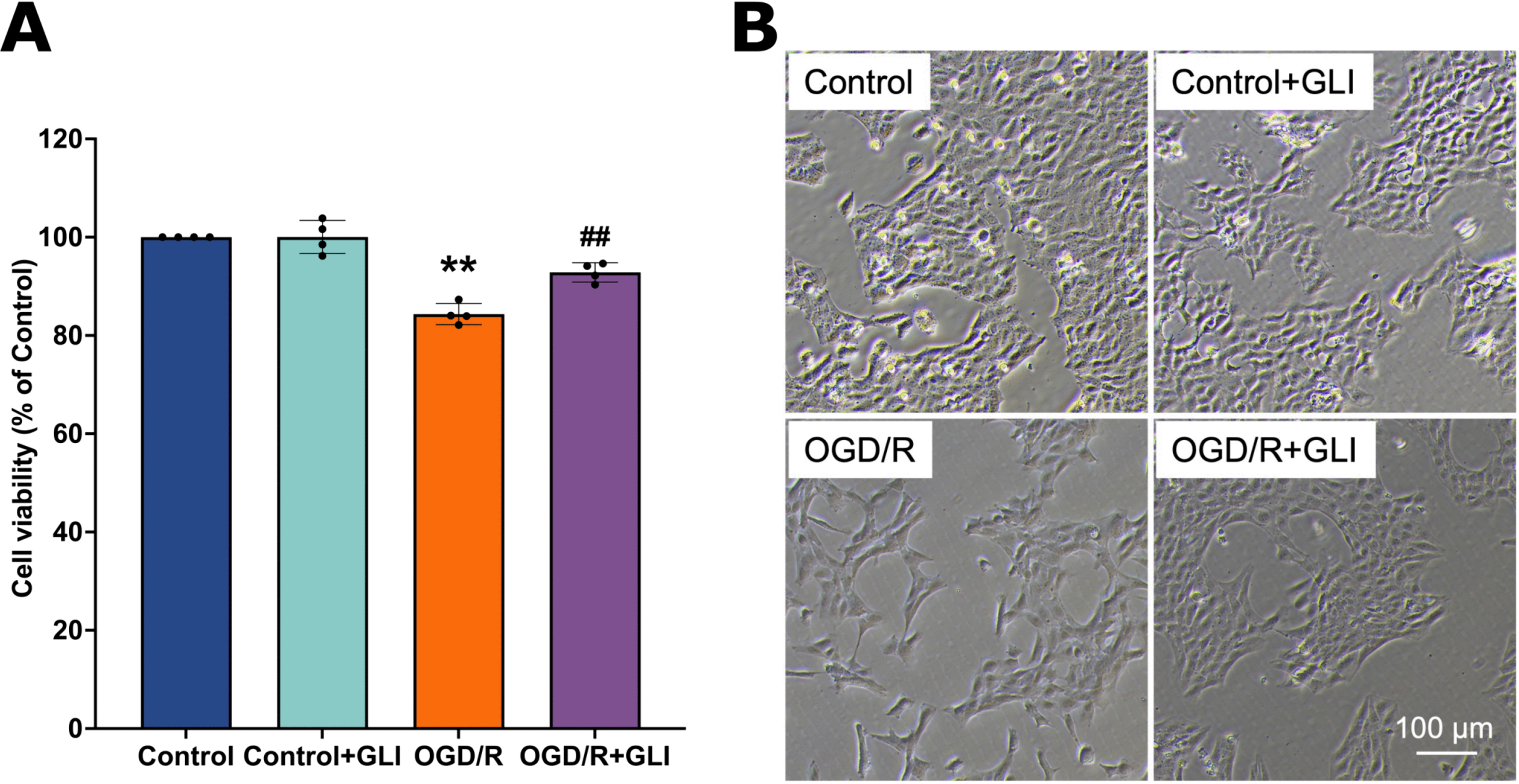
Glibenclamide attenuates OGD/R-induced reduction in cell viability and morphological alterations. (A) Cell viability of CTX-TNA2 cells under control and OGD/R conditions with or without 40 μM glibenclamide (GLI) treatment. (B) Phase-contrast images showing cell morphology under indicated conditions. Data are presented as mean ± SD from four independent experiments (n = 4). Statistical significance against control (100%) was determined using one-sample t-tests with Bonferroni correction. Comparisons among treatment groups were analyzed using ordinary one-way ANOVA followed by Tukey’s post hoc test. Scale bar: 100 μm. **p < 0.01 vs. Control; ##p < 0.01 vs. OGD/R.

To assess the effects of OGD/R and glibenclamide on cell viability, CTX-TNA2 cells were subjected to OGD for 6 h followed by 24 h of reoxygenation. Cell viability was significantly reduced under OGD/R conditions compared with the control group (84.34 ± 2.15%; *p* = 0.0021 vs. Control). This reduction was significantly attenuated by glibenclamide treatment during the reoxygenation phase (OGD/R + GLI: 92.82 ± 1.98%; *p* = 0.0031vs. OGD/R). In contrast, glibenclamide did not affect cell viability under normoxic conditions (Control + GLI: 100.05 ± 3.39%; *p* = 0.978 vs. Control) (Fig 1A). Phase-contrast imaging revealed morphological differences among the experimental groups (Fig 1B). Under normoxic conditions, CTX-TNA2 cells exhibited high cell density and typical morphology, with normal cell bodies and processes, regardless of GLI treatment. Compared with normoxic conditions, CTX-TNA2 cells subjected to OGD/R showed reduced cell density and morphological alterations, including enlarged cell bodies and a rounded appearance. In the OGD/R + GLI group, CTX-TNA2 cells displayed partial recovery in cell density and morphology compared with the OGD/R group.

### Glibenclamide alleviated OGD/R-induced cell swelling

**Fig 2:**
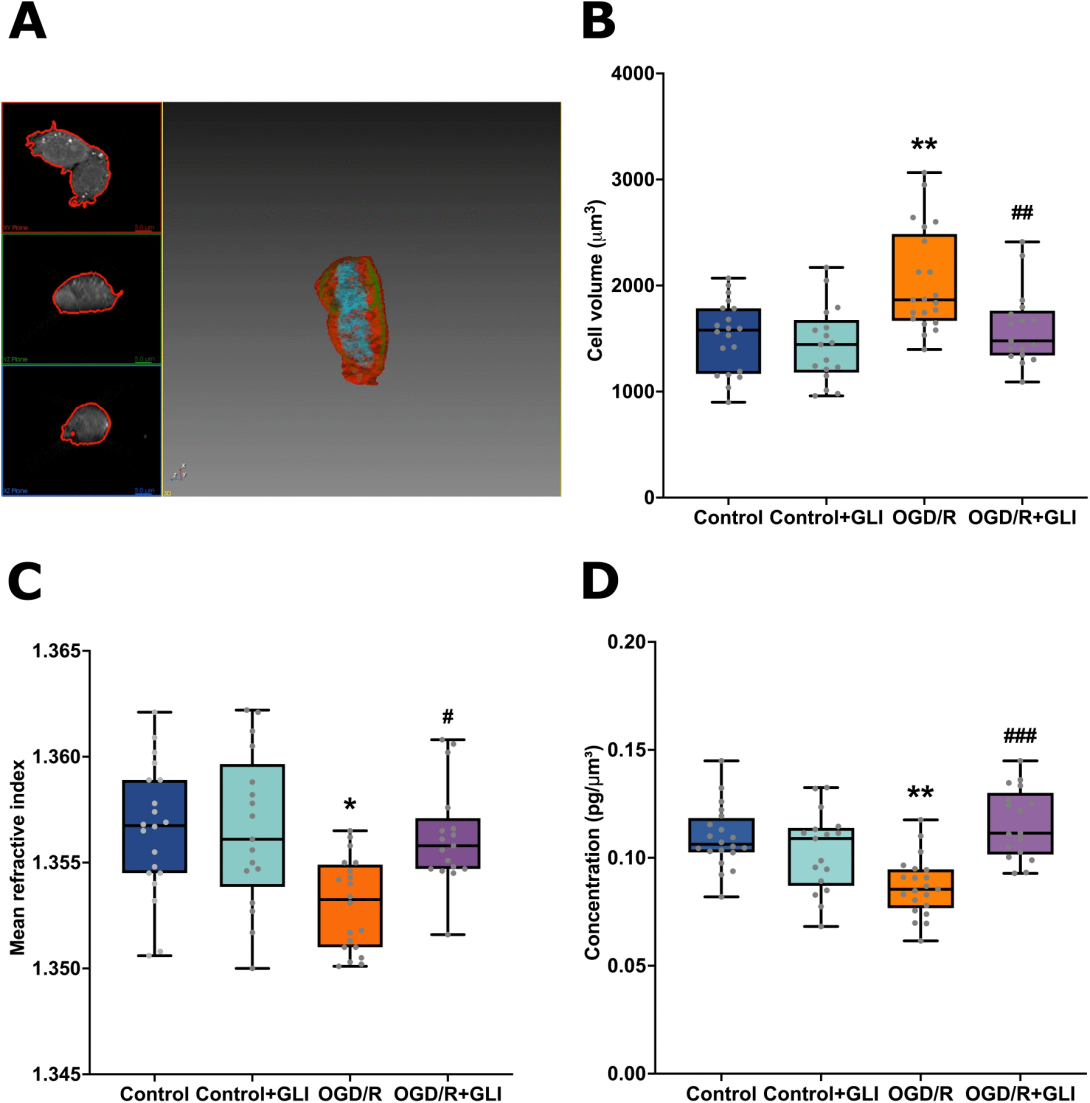
Glibenclamide attenuates OGD/R-induced cell swelling and associated biophysical alterations. (A) Representative three-dimensional tomography images of CTX-TNA2 cells acquired for quantitative volumetric analysis. Red, cell membrane; green, cytoplasm; blue, nucleus. (B) CTX-TNA2 cell volume under control and OGD/R conditions with or without GLI treatment. (C) Mean RI of cells under the indicated experimental conditions. (D) Intracellular concentration under the indicated experimental conditions. All measurements were performed at 12 h after reoxygenation. Each dot represents a single cell. Cells were collected from four independent biological experiments (n = 4). Data are displayed as median with interquartile range (IQR), with minimum and maximum values indicated. For statistical analysis, data were averaged per independent experiment, and comparisons were performed using one way ANOVA followed by Tukey’s multiple comparisons test. *p < 0.05 and **p < 0.01 vs. Control; #p < 0.05, ##p < 0.01 and ###p < 0.001 vs. OGD/R.

To characterize cell volume under OGD/R conditions, quantitative biophysical parameters were analyzed at an early reperfusion stage (12 h of reoxygenation). Representative three-dimensional reconstructions acquired using a Tomocube system are shown in Fig 2A, with the cell membrane shown in red, the cytoplasm in green, and the nucleus in blue. Astrocyte cell volume remained unchanged under normoxic conditions with or without GLI (Control: 1528.22 ± 81.89 μm³; Control + GLI: 1431.89 ± 136.42 μm³; p = 0.7861), but was significantly increased following OGD/R (2047.99 ± 174.77 μm³; p = 0.0014 vs. Control). This increase was significantly attenuated by GLI treatment during the reoxygenation phase (OGD/R + GLI: 1604.11 ± 172.47 μm³; p = 0.0048 vs. OGD/R), restoring cell volume to a level not different from the control group (p = 0.8799; Fig 2B).

To further characterize changes in cellular properties, mean RI and intracellular dry mass concentration were quantified at 12 h of reoxygenation. Both parameters remained unchanged under normoxic conditions (Control: RI = 1.3565 ± 0.0013, concentration = 0.1094 ± 0.0017; Control + GLI: RI = 1.3565 ± 0.0018, concentration = 0.1024 ± 0.0095; all p > 0.05). However, both mean RI and intracellular concentration were significantly reduced following OGD/R (RI: 1.3530 ± 0.0011, p = 0.0013 vs. Control; concentration: 0.0875 ± 0.0093, p < 0.0001 vs. Control). These reductions were significantly attenuated by GLI treatment during the reoxygenation phase (OGD/R + GLI: RI = 1.3562 ± 0.0016, p = 0.0058 vs. OGD/R; concentration = 0.1154 ± 0.0070, p < 0.0001 vs. OGD/R). In the OGD/R + GLI group, both parameters were not significantly different from those in the control group (all p > 0.05; Fig 2C, D).

### Glibenclamide reduced OGD/R-induced GFAP upregulation

**Fig 3:**
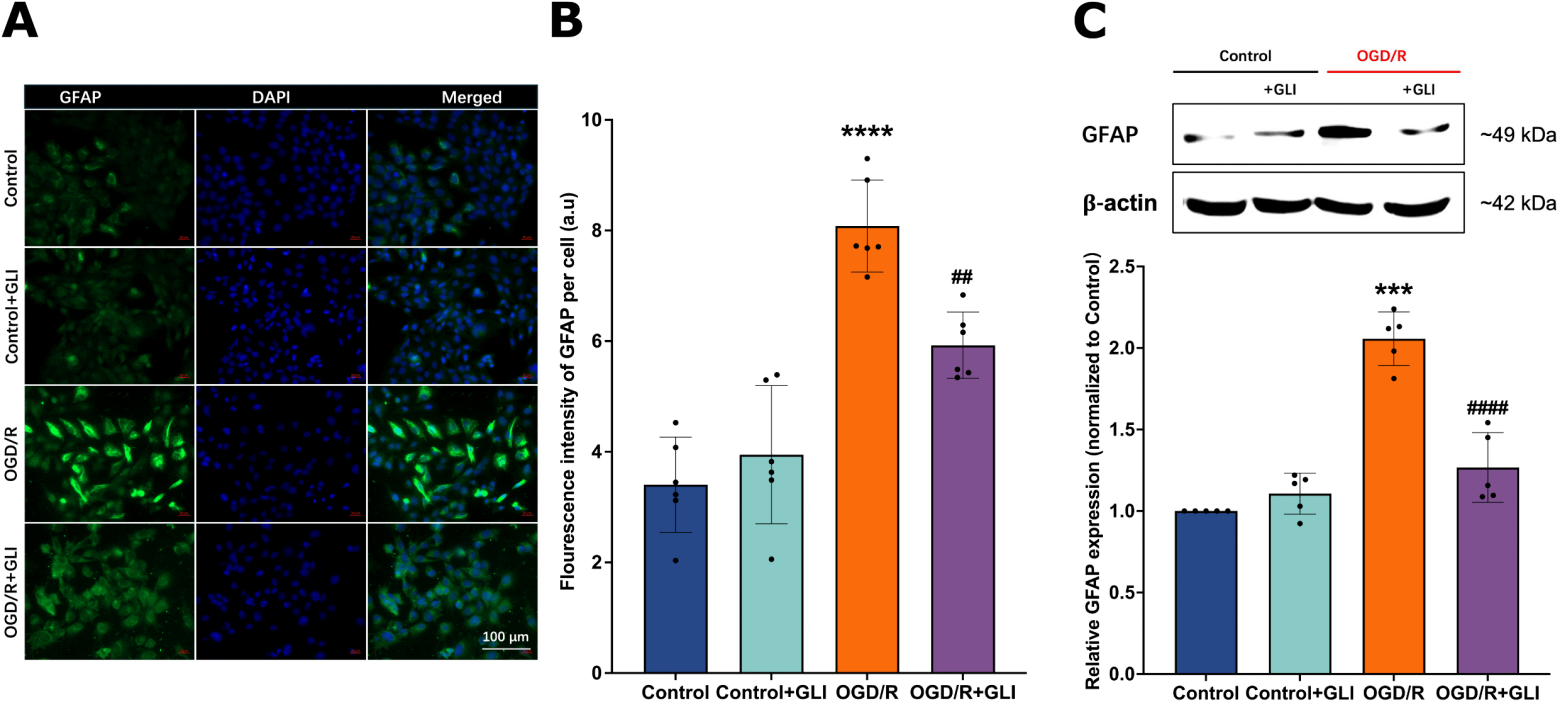
Glibenclamide suppresses OGD/R-induced GFAP upregulation. (A) Representative immunofluorescence images of GFAP (green) with nuclear counterstaining by DAPI (blue) in CTX-TNA2 cells under control and OGD/R conditions with or without 40 μM glibenclamide (GLI). (B) Quantification of GFAP fluorescence intensity per cell. (C) Representative western blot images and quantitative analysis of GFAP protein expression. Data are presented as mean ± SD from independent biological replicates. Immunofluorescence analyses were performed in six independent biological experiments (n = 6), whereas western blot analyses were performed in five independent biological experiments (n = 5). Statistical significance against normalized control values (1.0) was determined using one-sample t-tests with Bonferroni correction, while comparisons among treatment groups were analyzed using one-way ANOVA followed by Tukey’s multiple comparisons test. ***P < 0.001, ****P < 0.0001 vs. Control; ##P < 0.01, ####P < 0.0001 vs. OGD/R.

To evaluate the effects of glibenclamide on astrocyte reactivity under OGD/R conditions, GFAP expression was assessed by immunofluorescence staining and Western blot analysis. As shown in Fig 3A, OGD/R markedly increased GFAP immunoreactivity compared with the control group, whereas glibenclamide reduced GFAP signal intensity. Quantitative analysis demonstrated a significant increase in GFAP fluorescence intensity per cell following OGD/R (Control: 3.41 ± 0.88; OGD/R: 8.08 ± 0.87; p < 0.0001), which was significantly attenuated by glibenclamide treatment (OGD/R + GLI: 5.92 ± 0.59; *p* = 0.0030 vs. OGD/R; Fig 3B). Under normoxic conditions, GLI did not alter GFAP fluorescence intensity (Control + GLI: 3.95 ± 1.34 vs. Control: 3.406 ± 0.53; p = 0.7367). Consistent with the immunofluorescence findings, Western blot analysis showed that GFAP protein levels were significantly increased following OGD/R (OGD/R: 2.057 ± 0.164; p = 0.00027 vs. Control). This increase was significantly attenuated by GLI treatment during the reoxygenation phase (OGD/R + GLI: 1.267 ± 0.215; p = 0.00041 vs. OGD/R; Fig 3C). Under normoxic conditions, GLI did not affect GFAP protein expression (Control + GLI: 1.107 ± 0.126; p = 0.262 vs. Control).

### Glibenclamide attenuated OGD/R-induced oxidative stress

**Fig 4:**
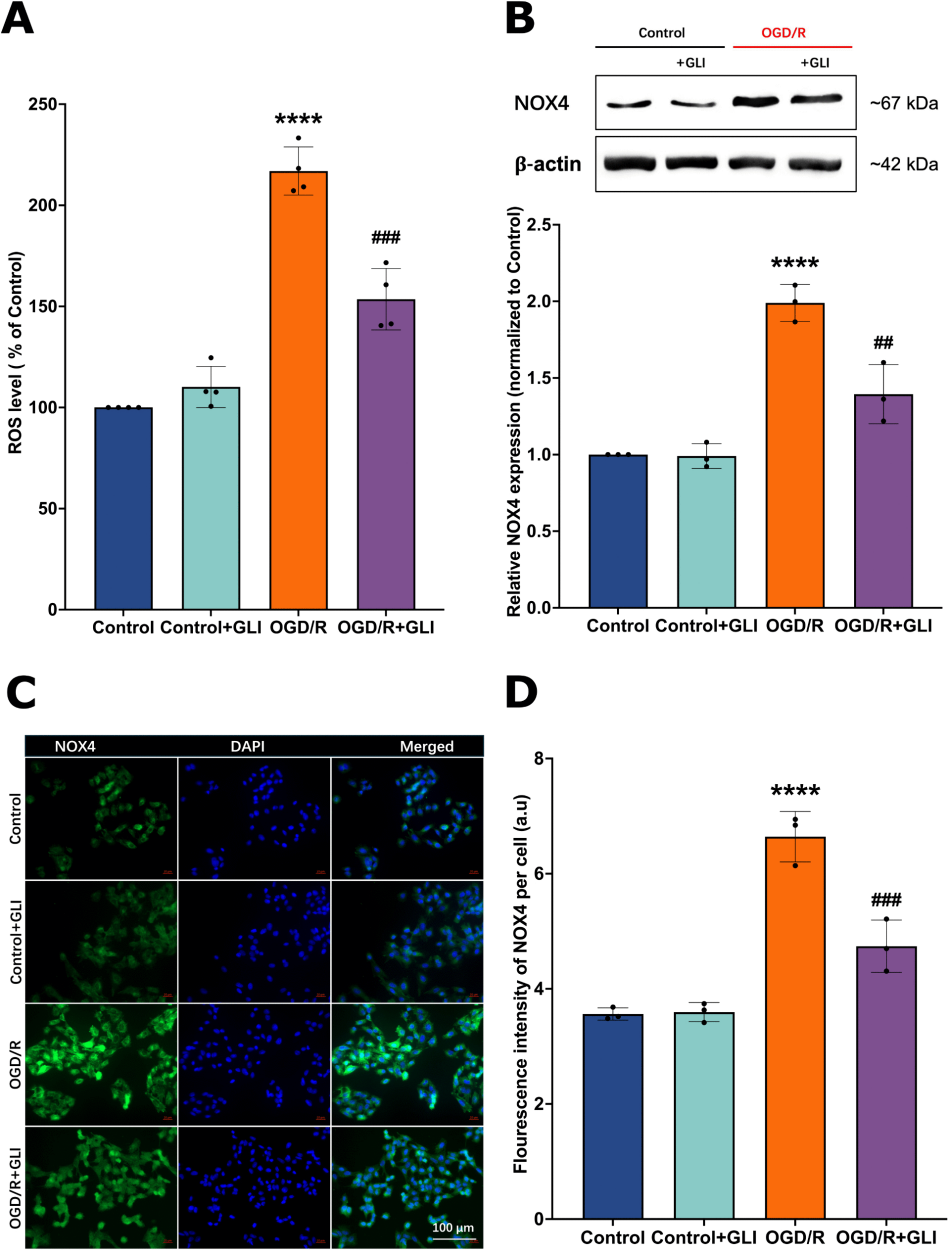
Glibenclamide attenuates OGD/R-induced oxidative stress and NOX4 upregulation in CTX-TNA2 cells. (A) Intracellular ROS levels in CTX-TNA2 cells under control and OGD/R conditions with or without 40 μM glibenclamide (GLI) treatment. (B) Representative western blot images and quantitative analysis of NOX4 protein expression. (C) Representative immunofluorescence images of NOX4 (green) with nuclear counterstaining by DAPI (blue). (D) Quantification of NOX4 fluorescence intensity per cell. Data are presented as mean ± SD from independent biological replicates. ROS data were obtained from four independent experiments (n = 4), whereas western blot and immunofluorescence analyses were performed in three independent experiments (n = 3). Statistical significance against normalized control values (100% or 1.0) was determined using one-sample t-tests with Bonferroni correction, while comparisons among treatment groups were analyzed using one-way ANOVA followed by Tukey’s multiple comparisons test. Scale bar: 100 μm. ****P < 0.0001 vs. Control; ##P < 0.01, ###P < 0.001 vs. OGD/R.

To examine changes in oxidative stress under OGD/R conditions, intracellular ROS levels were measured in CTX-TNA2 cells. Under normoxic conditions, ROS levels remained low and were not affected by GLI treatment (Control + GLI: 110.18 ± 10.14%; p = 0.236 vs. Control). OGD/R induced a marked increase in intracellular ROS levels (OGD/R: 216.98 ± 12.18%; p = 0.000124 vs. Control), which was significantly attenuated by glibenclamide treatment during the reoxygenation phase (OGD/R + GLI: 153.57 ± 15.22; p = 0.00079 vs. OGD/R; Fig 4A).

To further assess ROS-associated signaling, NOX4 expression was evaluated by Western blot analysis and immunofluorescence staining. Western blot analysis showed that NOX4 protein levels were significantly increased following OGD/R (OGD/R: 1.990 ± 0.120; p = 0.0020 vs. Control) and were significantly reduced by GLI treatment (OGD/R + GLI: 1.394 ± 0.194; p = 0.019 vs. OGD/R; Fig 4B). Under normoxic conditions, GLI did not affect NOX4 expression (Control + GLI: 0.991 ± 0.081; p = 0.9997 vs. Control). Immunofluorescence analysis showed a similar pattern, with NOX4 fluorescence intensity significantly increased following OGD/R (Control: 3.56 ± 0.11; OGD/R: 6.64 ± 0.44; p < 0.0001 vs. Control) and reduced by GLI treatment (OGD/R + GLI: 4.74 ± 0.45; p = 0.0004 vs. OGD/R; Fig 4D). No significant difference was observed between control and GLI-treated cells under normoxic conditions (Control + GLI: 3.60 ± 0.16; p > 0.05 vs. Control).

### Glibenclamide attenuated OGD/R-induced ER stress

**Fig 5:**
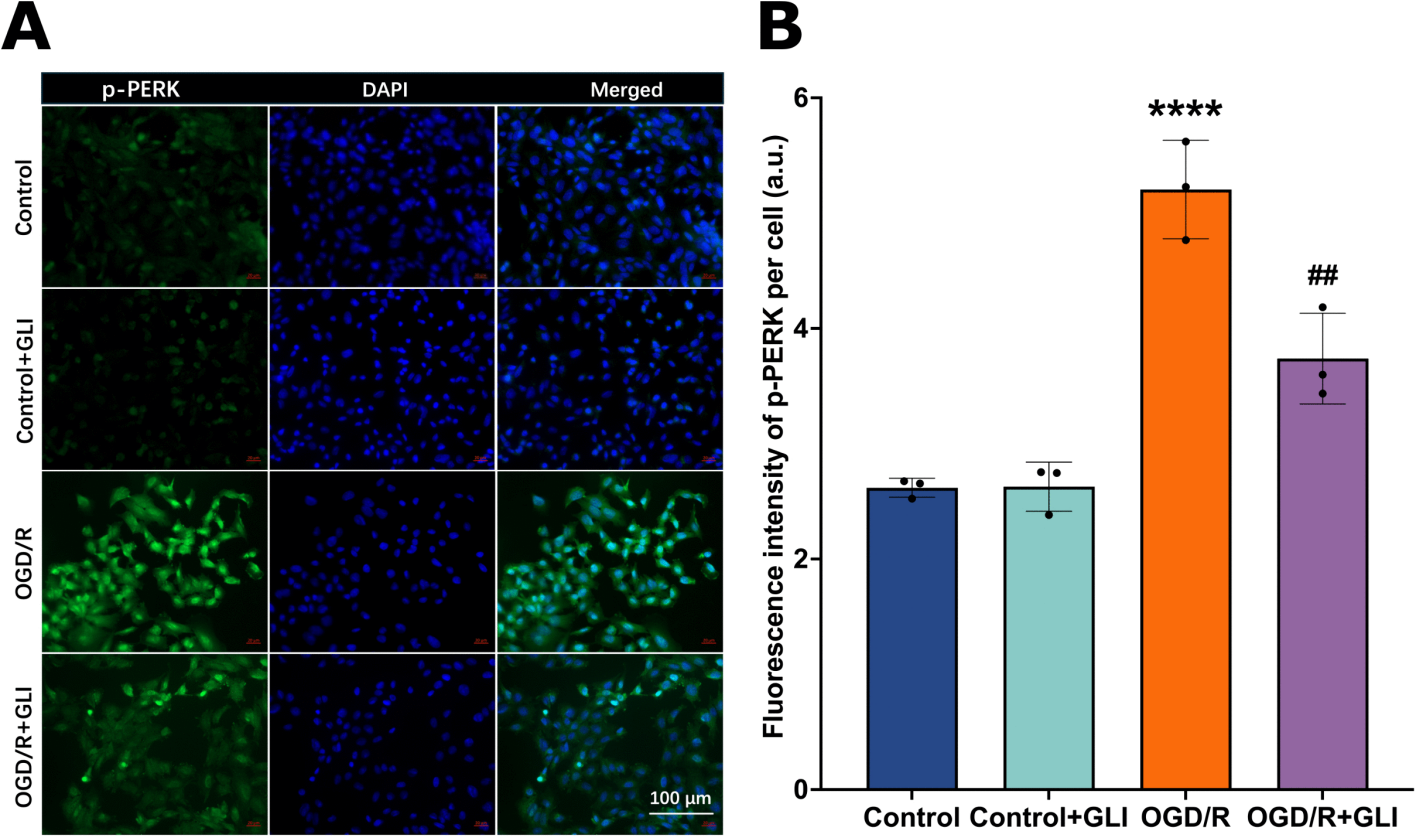
Glibenclamide attenuates ER stress signaling following OGD/R in CTX-TNA2 cells. (A) Representative immunofluorescence images of phosphorylated PERK (p-PERK, green) with nuclear counterstaining by DAPI (blue) in CTX-TNA2 cells under control and OGD/R conditions with or without 40 μM glibenclamide (GLI) treatment. **(B)** Quantification of p-PERK fluorescence intensity per cell. Data are presented as mean ± SD from three independent biological replicates (n = 3). Statistical significance was analyzed using one-way ANOVA followed by Tukey’s multiple comparisons test. Scale bar: 100 μm. ****p < 0.0001 vs. Control; ##p < 0.01 vs. OGD/R.

To determine whether ER stress is altered under OGD/R conditions, phosphorylated PERK (p-PERK) was examined in CTX-TNA2 cells by immunofluorescence staining. Representative fluorescence images (Fig 5A) showed low basal p-PERK immunoreactivity under normoxic conditions, a marked increase following OGD/R, and a apparent reduction with glibenclamide treatment during reoxygenation. Quantitative analysis demonstrated that p-PERK levels were significantly increased following OGD/R (Control: 2.62 ± 0.08; OGD/R: 5.20 ± 0.43; p < 0.0001 vs. Control), and this increase was significantly attenuated by GLI treatment during reoxygenation (OGD/R + GLI: 3.74 ± 0.40; p = 0.0021 vs. OGD/R; Fig 5B). Under normoxic conditions, GLI treatment alone did not affect p-PERK levels (Control + GLI: 2.63 ± 0.21; p = 0.995 vs. Control).

### Glibenclamide attenuated OGD/R-induced STAT3 activation

**Fig 6:**
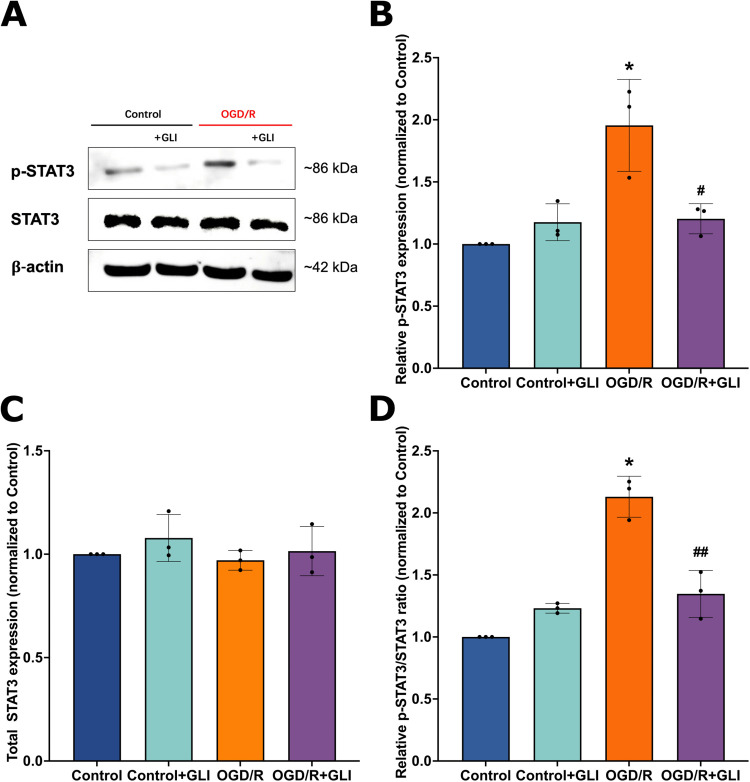
Glibenclamide inhibits STAT3 activation induced by OGD/R. (A) Representative Western blot images of p-STAT3 and total STAT3 in CTX-TNA2 cells under control and OGD/R conditions with or without 40 μM glibenclamide (GLI) treatment. (B) Quantification of p-STAT3 expression. (C) Quantification of total STAT3 expression. (D) Quantification of the p-STAT3/STAT3 ratio. Data are presented as mean ± SD from three independent biological replicates (n = 3). Statistical significance against normalized control values (1.0) was determined using one-sample t-tests with Bonferroni correction, while comparisons among treatment groups were analyzed using one-way ANOVA followed by Tukey’s multiple comparisons test. *P < 0.05 vs. Control; #P < 0.05, ##P < 0.01 vs. OGD/R.

STAT3 signaling was examined in CTX-TNA2 cells by evaluating phosphorylated STAT3 (p-STAT3) and total STAT3 expression using Western blot analysis (Fig 6A). Under normoxic conditions, basal p-STAT3 levels were low and remained unchanged following GLI treatment (Control + GLI: 1.176 ± 0.149; p = 0.28). OGD/R significantly increased p-STAT3 expression (OGD/R: 1.955 ± 0.372; p = 0.036 vs. Control), which was reduced following GLI treatment during the reoxygenation phase (OGD/R + GLI: 1.203 ± 0.122; p = 0.031 vs. OGD/R; Fig 6B). Total STAT3 protein levels remained comparable across all groups (Control: 1.000; Control + GLI: 1.079 ± 0.114; OGD/R: 0.971 ± 0.048; OGD/R + GLI: 1.015 ± 0.118; all p > 0.05; Fig 6C). The p-STAT3/STAT3 ratio was markedly increased following OGD/R (OGD/R: 2.130 ± 0.165; p = 0.011 vs. Control) and was reduced by GLI treatment (OGD/R + GLI: 1.348 ± 0.190; p = 0.041 vs. OGD/R; Fig 6D).

### Cell swelling, ER stress, and oxidative stress contributed to OGD/R-induced GFAP upregulation

**Fig 7:**
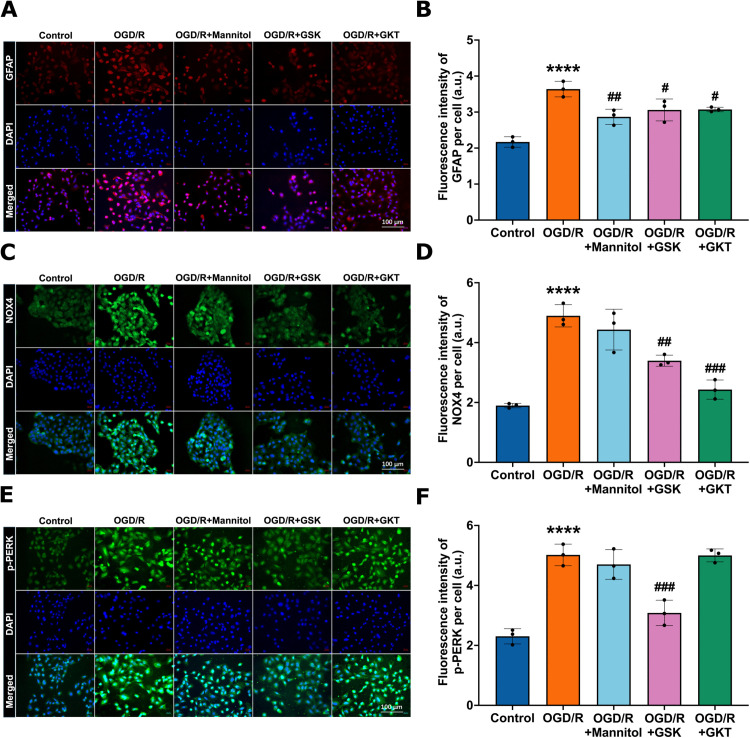
Modulation of downstream stress pathways influences GFAP expression and stress signaling. (A, C, E) Representative immunofluorescence images of GFAP (A), NOX4 (C), and phosphorylated PERK (p-PERK; E) in CTX-TNA2 cells under control and OGD/R conditions with or without 40 μM glibenclamide (GLI) treatment. (B, D, F) Quantitative fluorescence intensity per cell of GFAP (B), NOX4 (D), and p-PERK (F). Data are presented as mean ± SD from three independent biological replicates (n = 3). Statistical significance was analyzed using one-way ANOVA followed by Tukey’s multiple comparisons test. Scale bar: 100 μm. ****p < 0.0001 vs. Control; #p < 0.05, ##p < 0.01 and ###p < 0.001 vs. OGD/R. Abbreviations: GSK, GSK2606414 (PERK inhibitor); GKT, GKT137831 (NOX1/4 inhibitor).

To dissect the contribution of distinct stress pathways to astrocyte reactivity under OGD/R conditions, GFAP expression was examined by immunofluorescence staining (Fig 7A). Representative images show low basal GFAP immunoreactivity under normoxic conditions, a marked increase following OGD/R, and partial attenuation by hyperosmotic treatment or pharmacological inhibition of ER stress and NOX4 signaling. Quantitative analysis revealed that GFAP fluorescence intensity was significantly increased following OGD/R compared with control group (Control: 2.17 ± 0.15; OGD/R: 3.64 ± 0.22; p < 0.0001 vs. Control). This increase was attenuated by mannitol treatment (Mannitol: 2.87 ± 0.21; p = 0.0067 vs. OGD/R), as well as by the PERK inhibitor GSK2606414 (GSK: 3.06 ± 0.31; p = 0.0382 vs. OGD/R) and the NOX4 inhibitor Setanaxib (GKT: 3.07 ± 0.06; p = 0.0431 vs. OGD/R; Fig 7B).

To further evaluate oxidative stress signaling, NOX4 expression was assessed by immunofluorescence (Fig 7C). Representative images showed low basal NOX4 immunoreactivity under normoxic conditions, a marked increase following OGD/R, and differential modulation by hyperosmotic treatment or pharmacological inhibition of ER stress and NOX4 signaling. Quantitative analysis showed that NOX4 fluorescence intensity was markedly increased following OGD/R compared with the control group (Control: 1.90 ± 0.08; OGD/R: 4.90 ± 0.38; p < 0.0001 vs. Control). Mannitol treatment did not significantly affect OGD/R-induced NOX4 upregulation (Mannitol: 4.44 ± 0.67; p = 0.6054 vs. OGD/R). In contrast, treatment with the PERK inhibitor GSK2606414 significantly reduced NOX4 expression (GSK: 3.40 ± 0.19; p = 0.0054 vs. OGD/R), while the NOX4 inhibitor Setanaxib (GKT) markedly attenuated NOX4 upregulation (GKT: 2.43 ± 0.32; p = 0.0001 vs. OGD/R; Fig 7D).

ER stress signaling was assessed by p-PERK immunofluorescence staining (Fig 7E). Quantitative analysis showed that p-PERK fluorescence intensity was significantly increased following OGD/R compared with the control group (Control: 2.31 ± 0.10; OGD/R: 5.06 ± 0.23; p < 0.0001 vs. Control). Hyperosmotic treatment with mannitol did not significantly affect OGD/R-induced p-PERK upregulation (Mannitol: 4.69 ± 0.57; p = 0.64 vs. OGD/R). In contrast, treatment with the PERK inhibitor GSK2606414 significantly attenuated p-PERK expression under OGD/R conditions (GSK2606414: 3.08 ± 0.42; p = 0.0007 vs. OGD/R). Treatment with the NOX4 inhibitor Setanaxib (GKT) did not alter p-PERK levels following OGD/R (GKT: 5.00 ± 0.21; p = 0.997 vs. OGD/R; Fig 7F).

### ACM regulated p-STAT3 and GFAP expression

**Fig 8:**
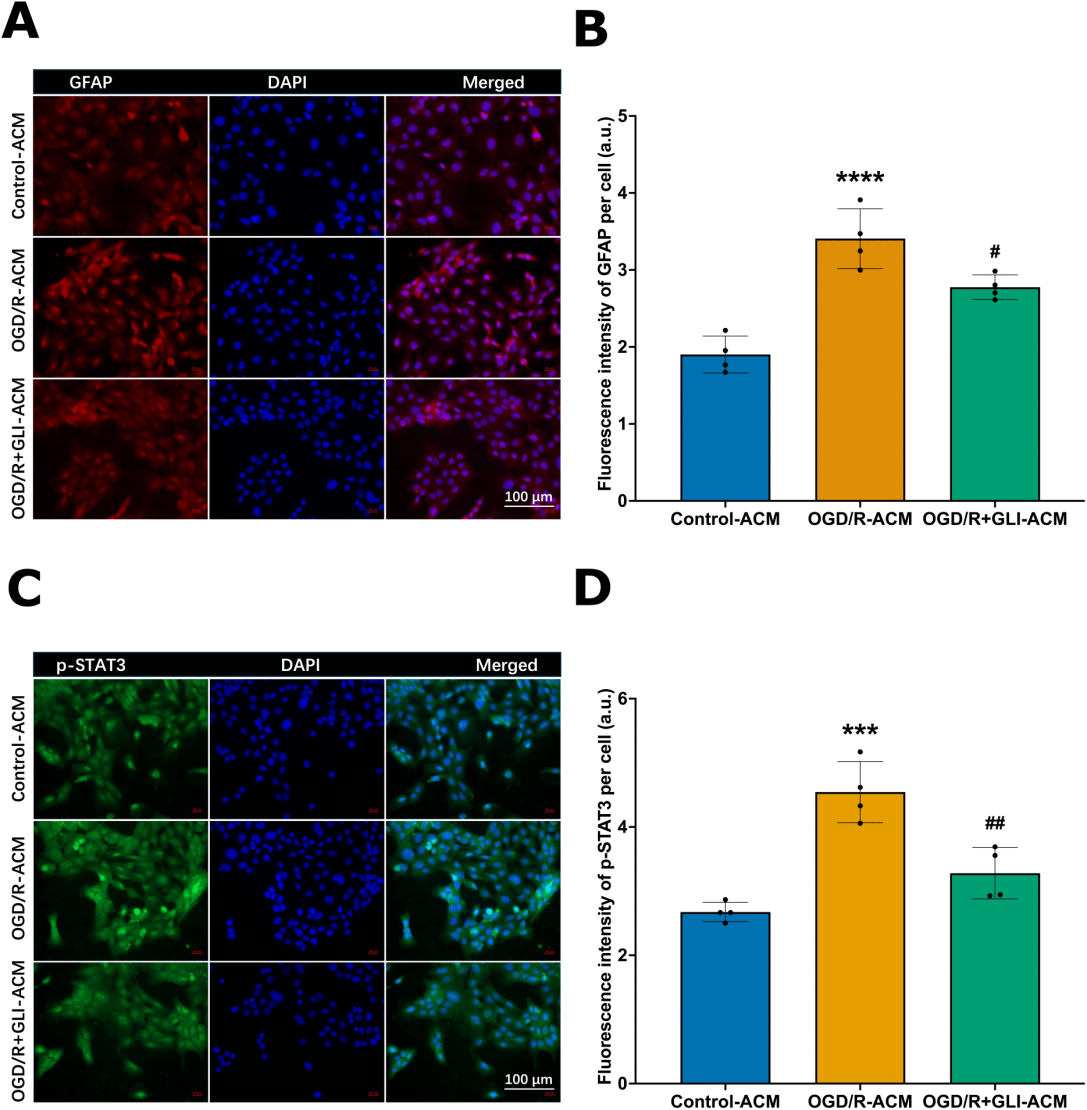
Effects of astrocyte-conditioned medium (ACM) on GFAP expression and STAT3 phosphorylation in recipient CTX-TNA2 cells. (A, C) Representative immunofluorescence images of GFAP (A) and phosphorylated STAT3 (p-STAT3; C) in recipient CTX-TNA2 cells treated with ACM collected from Control, OGD/R, or OGD/R + 40 μM glibenclamide (GLI)-treated donor cultures. (B, D) Quantitative fluorescence intensity of GFAP (B) and p-STAT3 (D). Data are presented as mean ± SD from four independent biological replicates (n = 4). Statistical significance was analyzed using one-way ANOVA followed by Tukey’s multiple comparisons test. Scale bar: 100 μm. ***p < 0.001, ****p < 0.0001 vs. Control-ACM; #p < 0.05, ##p < 0.01 vs. OGD/R-ACM.

To investigate the effects of ACM on astrocyte responses, naïve CTX-TNA2 cells were treated with ACM collected from donor cells subjected to different experimental conditions. The experimental groups included Control-ACM, OGD/R-ACM, and OGD/R + GLI-ACM. GFAP expression was assessed by immunofluorescence staining. Representative immunofluorescence images showed that exposure to OGD/R-ACM markedly increased GFAP immunoreactivity in recipient cells compared with Control-ACM group, whereas this effect was attenuated when ACM was derived from glibenclamide-treated donor cells (Fig 8A). Quantitative analysis demonstrated that GFAP fluorescence intensity was significantly increased in the OGD/R-ACM group compared with the Control-ACM group (Control-ACM: 1.90 ± 0.25; OGD/R-ACM: 3.41 ± 0.39; p < 0.0001 vs. Control-ACM). This increase was significantly attenuated by OGD/R + GLI-ACM group (OGD/R + GLI-ACM: 2.78 ± 0.16; p = 0.0264 vs. OGD/R-ACM; Fig 8B).

The recipient CTX-TNA2 cells exposed to OGD/R-ACM also exhibited markedly increased p-STAT3 immunoreactivity, which was attenuated when ACM was derived from glibenclamide-treated donor cells (Fig 8C). Quantitative analysis demonstrated that p-STAT3 fluorescence intensity was significantly increased in the OGD/R-ACM group compared with the Control-ACM group (Control-ACM: 2.68 ± 0.15; OGD/R-ACM: 4.54 ± 0.49; p = 0.0001 vs. Control-ACM). This increase was significantly attenuated in the OGD/R + GLI-ACM group (OGD/R + GLI-ACM: 3.28 ± 0.41; p = 0.0024 vs. OGD/R-ACM), with no significant difference compared with the Control-ACM group (p = 0.1049; Fig 8D). Together, these findings indicate that OGD/R-derived ACM promotes GFAP upregulation and STAT3 phosphorylation in recipient CTX-TNA2 cells, and that these responses are attenuated when ACM is collected from glibenclamide-treated donor cells.

In the present study, we investigated the effect of in vitro I/R injury (OGD/R) on CTX-TNA2 cells, an astrocyte cell line, with GFAP upregulation serving as the marker of astrocyte reactivity. We found that pharmacological inhibition of the SUR1-TRPM4 channel significantly attenuated cell swelling and astrocyte reactivity in CTX-TNA2 cells following OGD/R injury. To examine potential upstream and downstream signaling relationships, we used hyperosmotic unloading and pathway-specific inhibition. Hyperosmotic treatment suppressed GFAP upregulation without affecting p-PERK or NOX4 expression. In contrast, PERK inhibition reduced both p-PERK and NOX4 expression, whereas NOX inhibition selectively reduced NOX4. These dissociable pharmacological effects suggest that SUR1-TRPM4 -associated swelling may occur independently of the PERK-NOX4 cascade, while PERK-mediated ER stress may function upstream of NOX4-related oxidative signaling, with both pathways converging on astrocyte reactivity under OGD/R conditions. Conditioned-medium experiments further demonstrated that stressed CTX-TNA2 cells released soluble factors capable of inducing STAT3 phosphorylation and GFAP upregulation in naïve recipient cells, suggesting a potential contribution of paracrine signaling mechanisms to astrocyte reactivity. Together, these findings identify SUR1-TRPM4 channel may work as a regulator that couples volume dysregulation, intrinsic stress signaling, and intercellular propagation to shape astrocyte reactivity after OGD/R injury.

Although the immortalized cells used in this study may not fully reproduce the molecular, metabolic, and phenotypic characteristics of primary astrocytes in vivo, this simplified in vitro system enabled controlled investigation of OGD/R-associated swelling and stress-related signaling responses. Nevertheless, CTX-TNA2 cells have been widely used in previous studies investigating OGD/R-associated astrocyte reactivation, oxidative stress, inflammatory signaling, inflammasome activation, and GFAP-associated responses under ischemic conditions, including studies directly comparing CTX-TNA2 cells with primary astrocytes [42–44].

The present OGD/R-based experimental system also lacks blood-derived components and the complex multicellular interactions present in the ischemic brain microenvironment. Accordingly, the SUR1-TRPM4-associated responses observed in CTX-TNA2 cells may not fully reflect the pathological processes occurring in activated astrocytes within post-ischemic brain tissue in vivo. Despite these limitations, the present in vitro model still provides a controlled experimental framework for examining potential relationships between astrocytic swelling, stress-associated signaling pathways, and astrocyte reactivity under OGD/R conditions.

### SUR1-TRPM4 mediates astrocyte swelling and reactivity in response to OGD/R

Astrocyte swelling is increasingly recognized as an early cellular contributor to cytotoxic cerebral edema following I/R injury in vivo [45,46]. In parallel, in vitro OGD/R models using primary astrocytes have demonstrated rapid volumetric expansion during the early phase of reperfusion [47,48]. The SUR1-TRPM4 channel has emerged as a key molecular determinant of ionic dysregulation and cytotoxic edema following central nervous system injury. Unlike constitutively expressed ion channels, SUR1-TRPM4 is de novo transcriptionally induced under pathological conditions, including ischemic stroke, traumatic brain injury, and subarachnoid hemorrhage [20,49,50]. Functionally, SUR1 associates with TRPM4 to form a non-selective, Ca² ⁺ -activated monovalent cation channel that drives sustained Na⁺ influx and membrane depolarization, leading to intracellular ionic accumulation and the formation of an osmotic gradient that favors water entry and cellular swelling [38,49]. In addition to its role in Na ⁺ -dependent membrane depolarization and osmotic swelling, previous studies have suggested that SUR1-TRPM4 activity may also influence intracellular Ca² ⁺ signaling indirectly through regulation of membrane potential and secondary Ca² ⁺ influx pathways [23,38]. Such Ca² ⁺ -associated signaling mechanisms have been implicated in astrocyte stress responses, oxidative stress, and downstream inflammatory signaling following central nervous system injury [15]. These observations raise the possibility that Ca² ⁺ -dependent pathways may contribute to the stress-related signaling responses observed under the present experimental conditions. Previous studies have primarily focused on SUR1-TRPM4 function in endothelial cells and neurons, where its upregulation has been shown to drive blood-brain barrier disruption, neuronal depolarization-associated cytotoxic swelling, and secondary tissue injury following I/R injury [50,51]. However, accumulating evidence indicates that astrocytes also express SUR1-TRPM4 following ischemic insult, and channel activation may directly perturb ionic homeostasis and volume regulation in astrocytes [24,49]. This raises the possibility that the channel influences astrocyte ionic balance and cell volume [52,53]. In this context, the biophysical measurements in the present study revealed a characteristic swelling phenotype during early reoxygenation, manifested by increased cell volume accompanied by reduced intracellular concentration and mean RI. This pattern is consistent with osmotic water influx and cytoplasmic dilution rather than cellular growth, in line with previous reports of water-driven astrocyte swelling under ischemic conditions [47,48,54]. Accordingly, inhibition of SUR1-TRPM4 with glibenclamide during OGD/R significantly reduced cell swelling and increase cell viability. These findings are consistent with previous reports demonstrating that glibenclamide inhibits SUR1-TRPM4 channel activity and reduces pathological Na ⁺ -dependent cation influx following ischemic injury [23,26].

Astrocyte reactivity represents a common cellular response to central nervous system injury and is characterized by morphological alterations and increased expression of intermediate filament proteins such as GFAP [55,56]. Reactive astrocytes can exert context-dependent effects, including modulation of inflammatory responses and regulation of the local neural microenvironment following ischemic insult, which could contribute to both protective and detrimental effects on the existing injury. GFAP upregulation is widely used as a hallmark of astrocyte reactivation across diverse central nervous system injury paradigms [57–59]. Whether astrocyte swelling directly drives astrocyte reactivation and the underlying mechanisms remain unresolved.

Our morphological and immunological analyses demonstrated that CTX-TNA2 cell reactivity occurs following OGD/R, as evidenced by cellular hypertrophy and increased GFAP expression. Pharmacological inhibition with glibenclamide attenuated these changes, suggesting that SUR1-TRPM4 activity contributes to the development of astrocyte reactivity after ischemic injury. Given that SUR1-TRPM4 activation promotes pathological ion influx and cell swelling, these findings indicate that astrocyte swelling may be associated with the development of astrocyte reactivity. This interpretation is further supported by the observation that hyperosmotic modulation with mannitol, which reduces cell swelling, similarly attenuated GFAP upregulation. Together, these findings support a link between SUR1-TRPM4-associated swelling and astrocyte reactivity in CTX-TNA2 cells under OGD/R conditions.

### SUR1-TRPM4-associated swelling responses are linked to intracellular stress signaling

Oxidative stress is a major pathogenic component of aging and neurological disease. While low levels of ROS function as redox signaling mediators, excessive ROS production disrupts cellular redox balance and promotes damage to proteins, lipids, and nucleic acids. In the central nervous system, NOX4 has been identified as an important enzymatic source of pathological ROS and is induced by ischemic and inflammatory stress, contributing to sustained oxidant production and redox-sensitive signaling [60,61]. In astrocytes, increased NOX4 expression has been reported in neurodegenerative disease models and is associated with mitochondrial dysfunction, lipid peroxidation, and glial pathology [62]. Beyond direct oxidative injury, NOX4-derived ROS also engage inflammatory and redox-dependent signaling pathways that regulate astrocyte reactivity and neuroinflammatory responses [59,63,64]. In line with these observations, we observed increases in intracellular ROS levels and NOX4 expression in CTX-TNA2 cells following OGD/R. Moreover, pharmacological inhibition of NOX activity by GKT137831 reduced GFAP upregulation, suggesting an association between NOX4-dependent oxidative signaling and astrocyte reactivity under OGD/R conditions.

ER stress is an important mediator of astrocyte dysfunction following I/R injury. Activation of the PERK branch of the UPR integrates multiple ischemia-associated stressors, including ionic imbalance, metabolic perturbation, and protein misfolding, and has been implicated in astrocyte reactivation and neuroinflammatory responses [30,65–68]. Consistent with these observations, we detected increased PERK phosphorylation in CTX-TNA2 cells after OGD/R. Furthermore, pharmacological inhibition of PERK with GSK2606414 reduced GFAP upregulation, suggesting that PERK-dependent ER stress is associated with astrocyte reactivity following OGD/R.

Mechanistic studies have shown that ER stress and oxidative stress form an interrelated stress network. NOX4-derived ROS localized to intracellular membranes, including the ER, can activate PERK signaling under conditions of metabolic and oxidative stress, whereas ER stress has also been reported to amplify oxidative injury through redox-sensitive pathways [63,66]. These observations provide a conceptual framework for coordinated PERK and NOX4 engagement during ischemic injury. Importantly, although ER stress and oxidative stress can engage in bidirectional crosstalk in multiple cellular systems [63], our findings suggest a preferential PERK-NOX4 signaling association in CTX-TNA2 cells subjected to OGD/R. Pharmacological inhibition of PERK significantly suppressed NOX4 upregulation, whereas NOX inhibition did not alter PERK phosphorylation, suggesting that NOX4 activation depends on PERK-dependent ER stress but is not sufficient to drive ER stress signaling.

The results of this study demonstrate that SUR1-TRPM4 activation, cell swelling, oxidative stress, and ER stress contribute to the development of astrocyte reactivity. However, the interactions among these processes have not previously been clearly defined. In the present study, inhibition of SUR1-TRPM4 with glibenclamide reduced NOX4 expression, ROS production, and PERK activation, supporting an association between SUR1-TRPM4 and downstream oxidative stress- and ER stress-related signaling responses under OGD/R conditions. Interestingly, hyperosmotic treatment designed to reduce cell swelling did not affect NOX4 expression or PERK activation, suggesting that SUR1-TRPM4-associated responses may influence oxidative and ER stress signaling through mechanisms that extend beyond cell swelling alone.

In summary, these findings support a model in which SUR1-TRPM4 is associated with PERK-dependent ER stress and downstream NOX4-driven oxidative signaling. This interconnected stress network may amplify intracellular stress responses and contribute to astrocyte reactivity. Our data indicate that this interaction is organized along a preferential PERK-to-NOX4 signaling axis in astrocytes under OGD/R conditions.

### STAT3-associated paracrine signaling under OGD/R conditions

STAT3 is an important regulator of stress-associated astrocyte responses, with phosphorylation of STAT3 closely associated with reactive changes and glial scar formation in multiple central nervous system injury models, including ischemia [36,69]. In astrocytes, activation of the JAK2-STAT3 pathway precedes and has been associated with GFAP upregulation, a key transcriptional hallmark of astrocyte reactivity [70,71]. Mechanistically, phosphorylated STAT3 binds regulatory elements of the GFAP promoter, positioning STAT3 activation as a transcriptional switch linking stress- and inflammation-associated signaling to astrocyte structural remodeling.

In astrocytes, STAT3 activation is primarily mediated by gp130-family cytokines, including interleukin-6 (IL-6) and leukemia inhibitory factor [11,72]. These cytokines are robustly induced by ER stress and oxidative stress and activate the JAK-STAT3 pathway in both autocrine and paracrine modes [30,72]. Through this mechanism, stressed astrocyte propagate reactive signaling to neighboring cells, amplifying astrocyte reactivity [73].

Consistent with this framework, we observed marked STAT3 phosphorylation in CTX-TNA2 cells following OGD/R, which was significantly attenuated by inhibition of SUR1-TRPM4. Because donor cells subjected to OGD/R remained exposed to their own conditioned medium during the experimental period, the observed STAT3 phosphorylation under OGD/R conditions may reflect combined contributions from intracellular stress responses and paracrine signaling mediated by stress-associated soluble factors released by injured cells. ACM collected from OGD/R-injured donor cells was sufficient to induce both STAT3 phosphorylation and GFAP upregulation in naïve recipient CTX-TNA2 cells, suggesting that soluble factors released under OGD/R conditions may propagate astrocyte reactivity in neighboring cells. In contrast, ACM derived from glibenclamide-treated OGD/R astrocytes failed to elicit STAT3 phosphorylation or GFAP upregulation, indicating that SUR1-TRPM4 activity in donor cells may influence STAT3-associated paracrine signaling under OGD/R conditions. However, the investigation of specific soluble mediators responsible for the observed recipient-cell responses will be important for further understanding of the mechanisms.

Together, these findings support a model in which SUR1-TRPM4-dependent stress signaling in astrocytes promotes the release of soluble factors that activate STAT3 in neighboring cells. Although the specific paracrine mediators were not identified in the present study, IL-6 family cytokines are well-established STAT3 activators in reactive astrocytes under conditions of ER and oxidative stress [30,72]. Within this framework, STAT3 phosphorylation may represent a potential convergence point through which intracellular stress responses and intercellular inflammatory signaling contribute to astrocyte reactivity under OGD/R conditions.

Although the molecular composition of ACM was not directly characterized in the present study and likely represents a complex mixture of cytokines, lipids, and other stress-associated soluble mediators, the observed recipient-cell responses were reproducibly detected under standardized culture conditions and ACM preparation procedures, supporting a potential contribution of ACM-associated paracrine signaling under OGD/R conditions. The identification of the specific ACM-associated mediators responsible for the observed recipient-cell responses warrants further investigation.

### Integrated model of astrocyte stress signaling under OGD/R conditions

**Fig 9:**
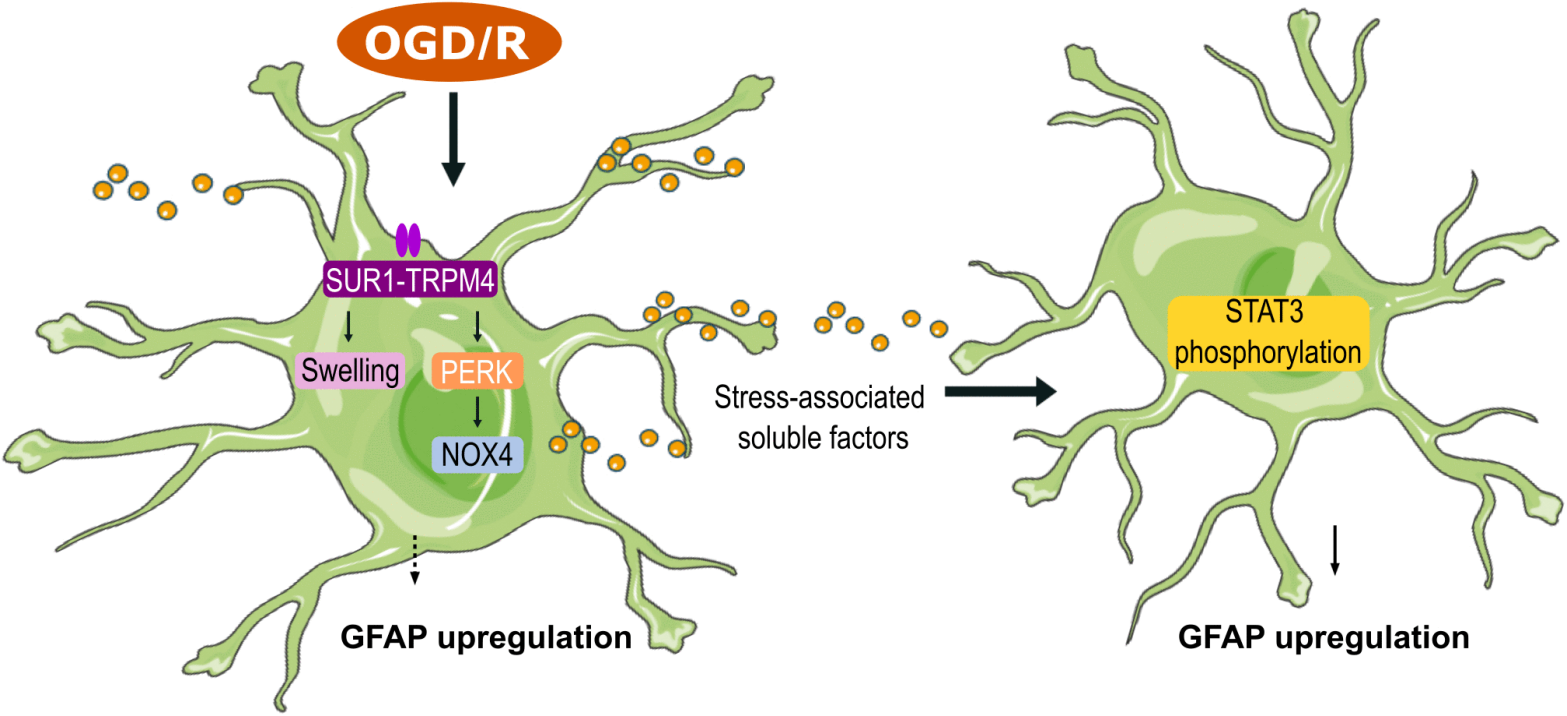
Proposed schematic model of SUR1-TRPM4-associated stress-related signaling responses under OGD/R conditions. OGD/R activates SUR1-TRPM4 and promotes astrocyte swelling in CTX-TNA2 cells, accompanied by increased PERK-related ER stress signaling and NOX4-associated oxidative stress. PERK-NOX4 signaling is linked to GFAP upregulation under OGD/R conditions. Stress-associated soluble factors released during OGD/R may contribute to STAT3 phosphorylation, GFAP upregulation, and astrocyte reactivity in neighboring cells through paracrine signaling. Purple: SUR1-TRPM4; pink box: cell swelling; orange box: PERK; light blue box: NOX4; yellow box: STAT3 phosphorylation; yellow dots: stress-associated soluble factors. Icons in this figure were adapted from Smart Servier Medical Art (https://smart.servier.com), licensed under a Creative Commons Attribution 3.0 Unported License.

The present findings support a working model in which SUR1-TRPM4-associated ionic dysregulation and swelling responses are linked to downstream stress-related signaling pathways in CTX-TNA2 cells subjected to OGD/R. Within this framework, PERK-associated ER stress, NOX4-related oxidative signaling, and STAT3-associated signaling responses may represent interconnected components of a broader intracellular stress network. ACM-mediated responses observed in recipient CTX-TNA2 cells suggest that soluble mediators released under OGD/R conditions may contribute to stress-associated intercellular signaling. Pharmacological inhibition of SUR1-TRPM4 with glibenclamide attenuated multiple stress-associated responses observed under the present experimental conditions, including swelling-related changes, oxidative stress-associated signaling, ER stress-associated signaling, and STAT3 phosphorylation. Together, these observations suggest potential associations between SUR1-TRPM4 activity and downstream stress-related signaling responses in this in vitro astrocyte injury model (Fig 9).

## Conclusion

In conclusion, the present findings suggest that, beyond its established role in astrocyte swelling, SUR1-TRPM4 may also contribute to astrocyte reactivity and stress-associated signaling responses under OGD/R conditions, accompanied by reduced cell viability. These responses were associated with NOX4-specific oxidative stress, ER stress signaling, and paracrine signaling mechanisms. The conclusions in this study further suggest a potential role for SUR1-TRPM4 in modulating both astrocyte swelling and astrocyte reactivity under in vitro conditions mimicking ischemic injury. Further studies using primary astrocytes, multicellular co-culture systems, and in vivo ischemia models will be important to further clarify the physiological relevance and mechanistic basis of these findings.

## Supporting information

S1 Data Underlying data for all figures and statistical analyses. (XLSX)

S1 File Original experimental images underlying the reported results. (PDF)
