# Supplementary material for: Inhibition of SUR1-TRPM4 attenuates astrocyte swelling and reactivity under oxygen-glucose deprivation/reoxygenation
Source: PLoS One. 2026 Jun 24;21(6):e0352151. doi: 10.1371/journal.pone.0352151 (PMC13293404; doi:10.1371/journal.pone.0352151)
Supplement: S1 File — (PDF) [file pone.0352151.s002.pdf]

S1 File. Raw images corresponding to Fig. 1–8

Part I. Bright-field images

Fig. 1. Representative bright-field images of CTX-TNA2 astrocytes. Boxed regions indicate areas selected for higher magnification. Scale bar: 100 µm. GLI: glibenclamide.

Control

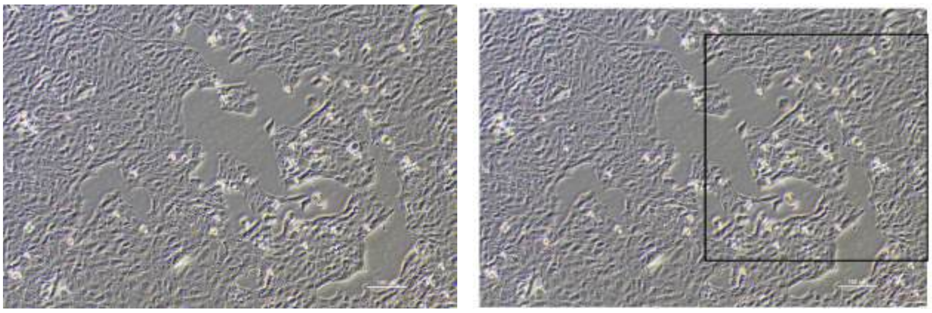

Control+GLI

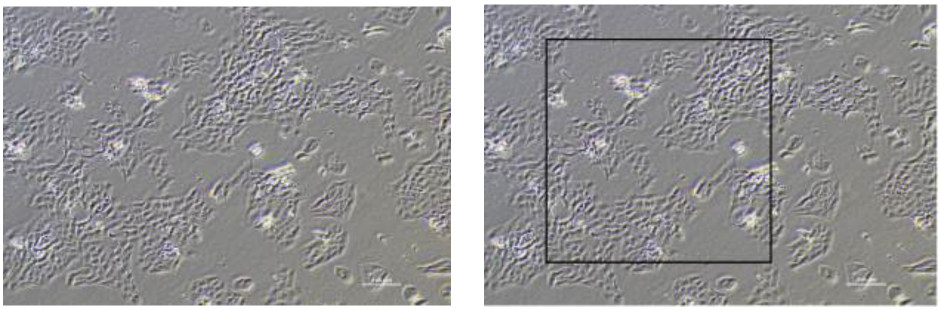

OGD/R

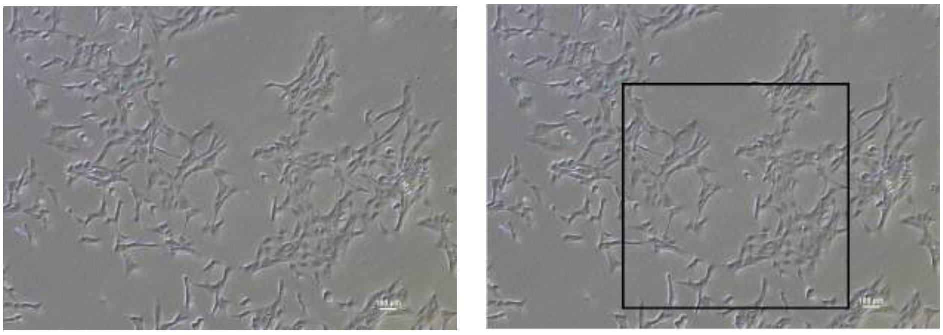

OGD/R+GLI

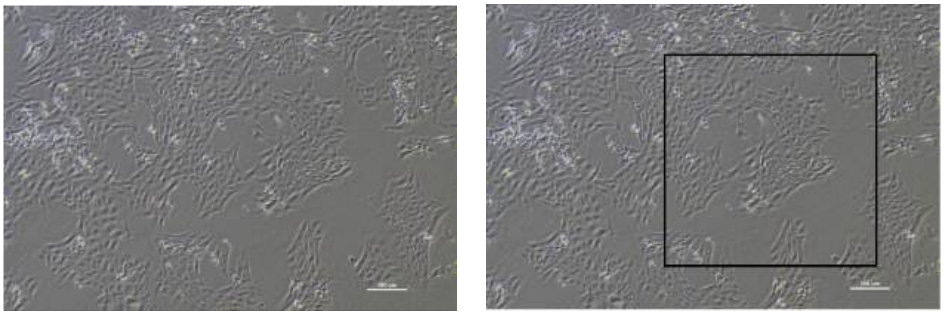

Part II. Holotomography images

Fig. 2. Representative holotomography images of CTX-TNA2 astrocytes. Raw refractive index (RI) slices in the XY (A), XZ (B), and YZ (C) planes are shown with corresponding segmentation outlines. Representative three-dimensional (3D) reconstructions from different viewing angles are presented in (D–F). Scale bar: 5.0 µm. Images were acquired using a holotomography system (Tomocube).

A. XY plane

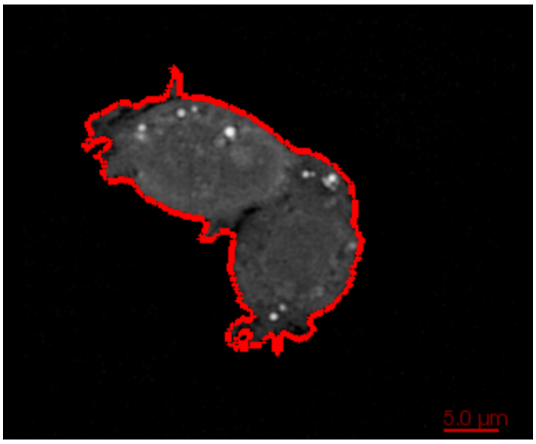

B. XZ plane

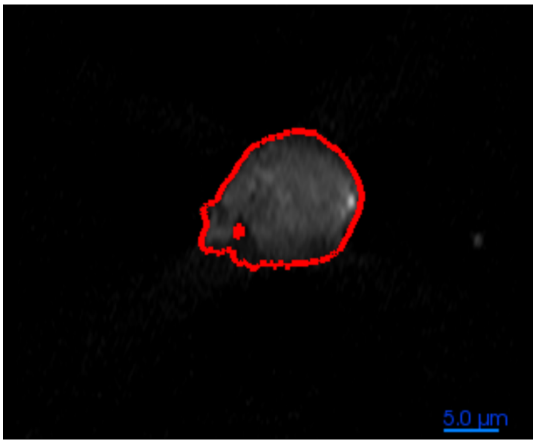

C. YZ plane

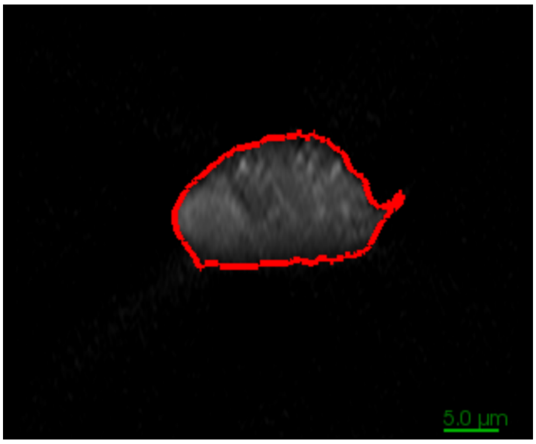

D. 3D View 1

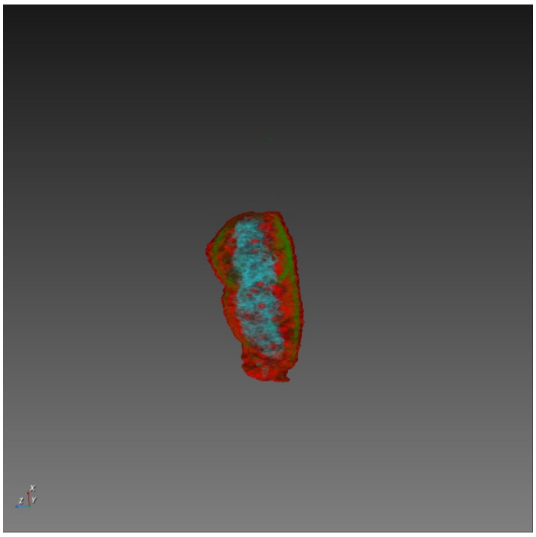

E. 3D View 2

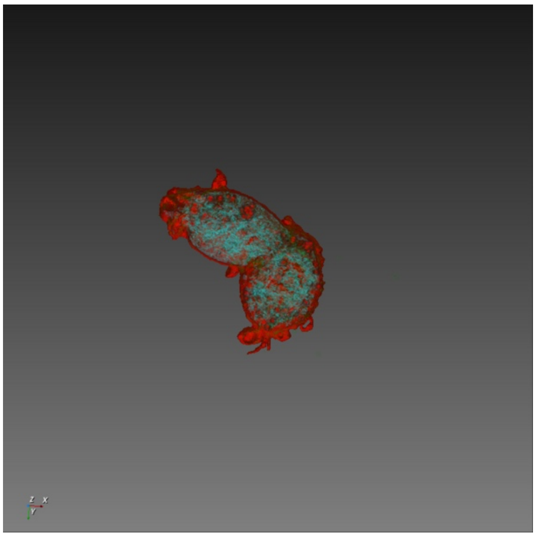

F. 3D View 3

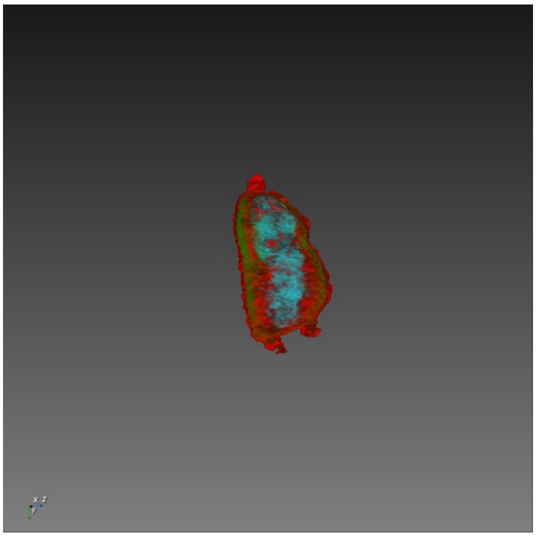

Part III. Immunofluorescence images

Fig. 3. Representative immunofluorescence images of GFAP in CTX-TNA2 astrocytes. GFAP is shown in green, and nuclei are counterstained with DAPI (blue). Scale bars: 20 μm. GLI: glibenclamide.

Control

GFAP

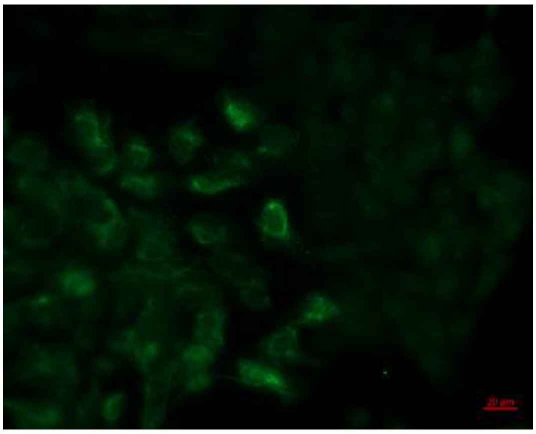

DAPI

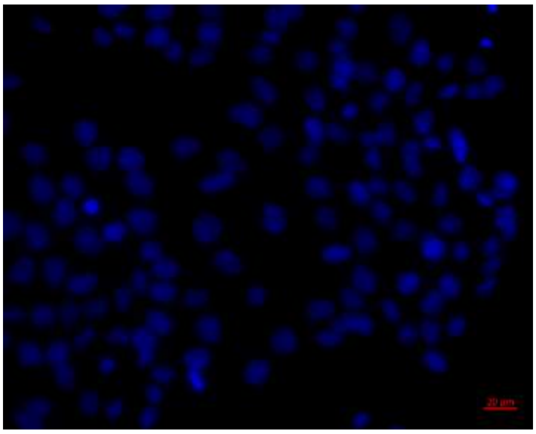

Merge

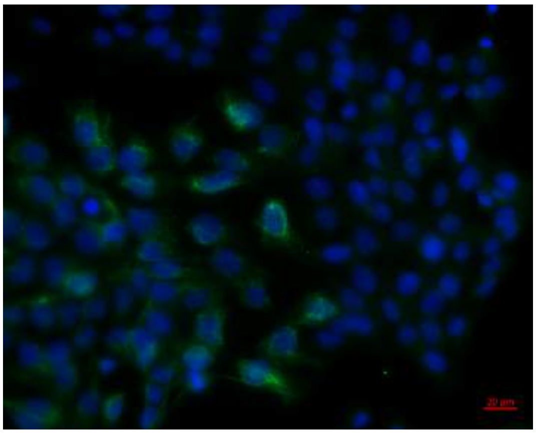

Control + GLI

GFAP

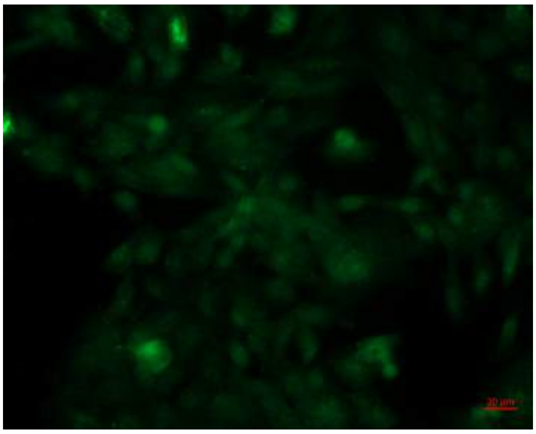

DAPI

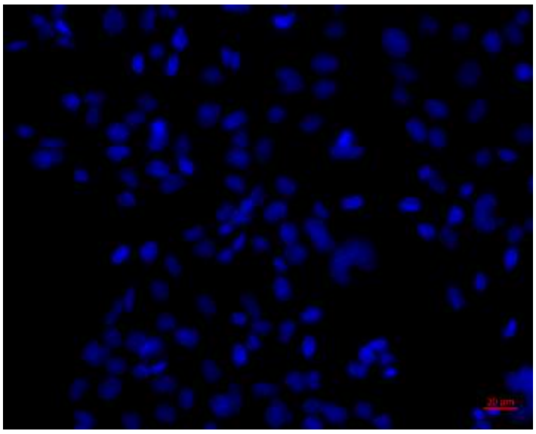

Merge

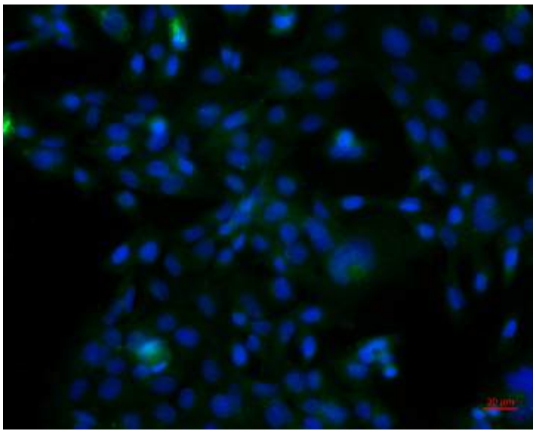

OGD/R

GFAP

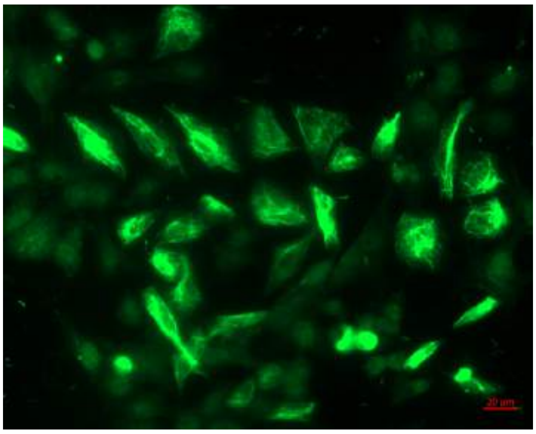

DAPI

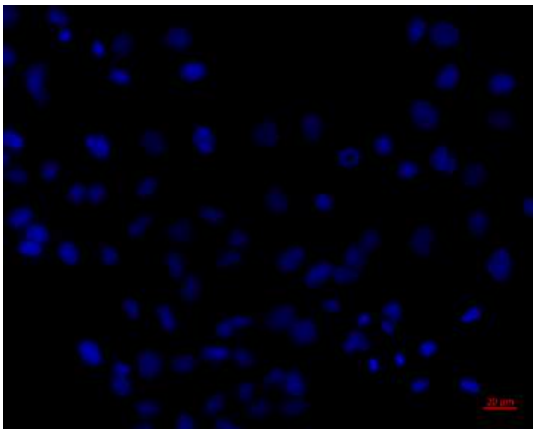

Merge

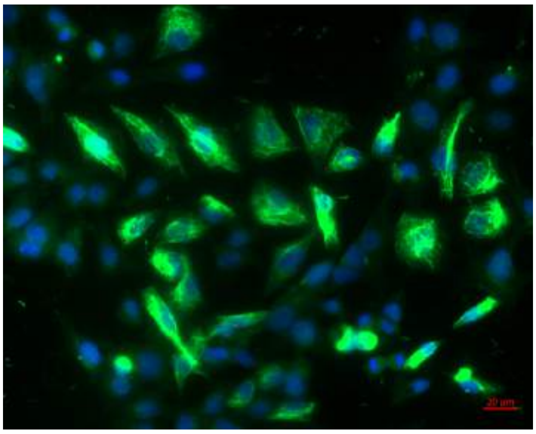

OGD/R + GLI

GFAP

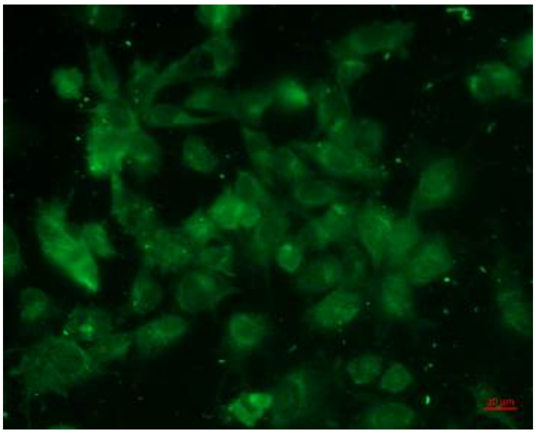

DAPI

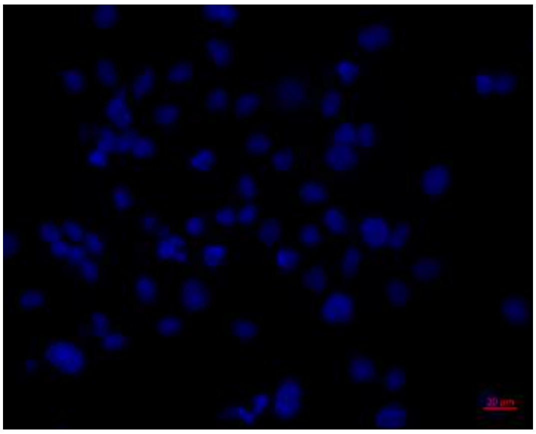

Merge

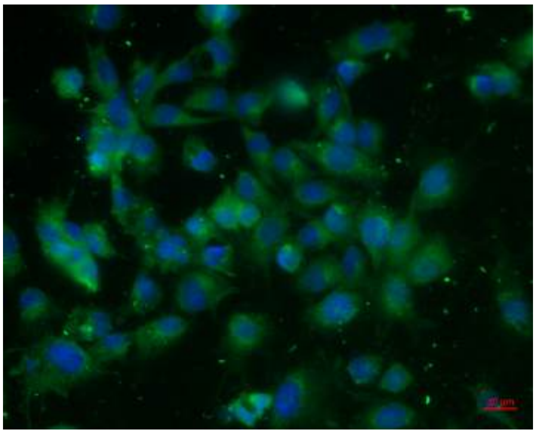

Fig. 4. Representative immunofluorescence images of NOX4 in CTX-TNA2 astrocytes. NOX4 is shown in green, and nuclei are counterstained with DAPI (blue). Scale bars: 20 μm. GLI: glibenclamide.

Control

NOX4

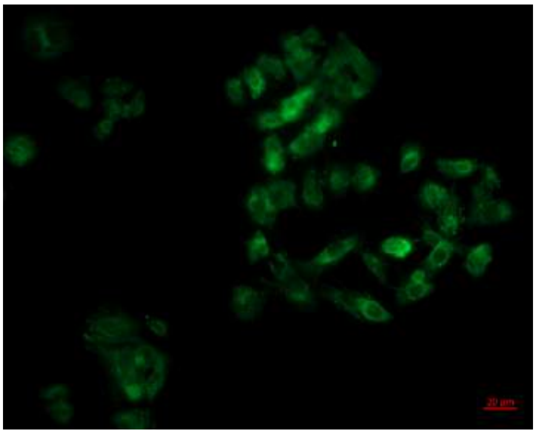

DAPI

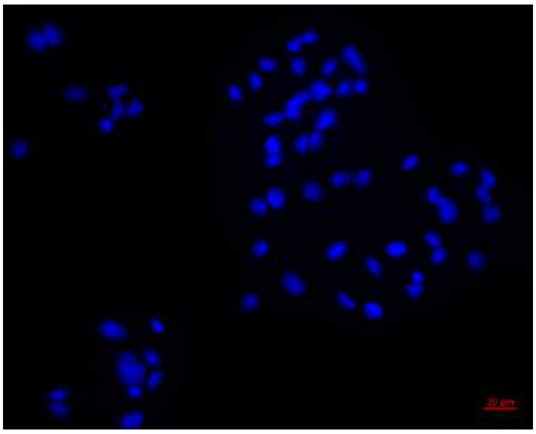

Merge

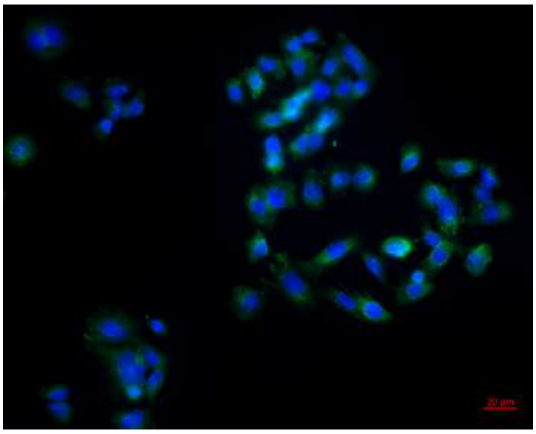

Control +GLI

NOX4

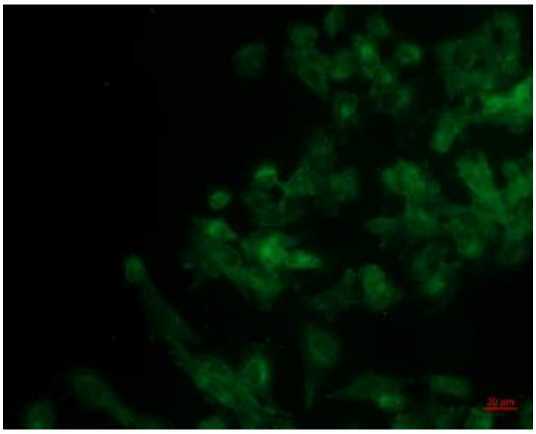

DAPI

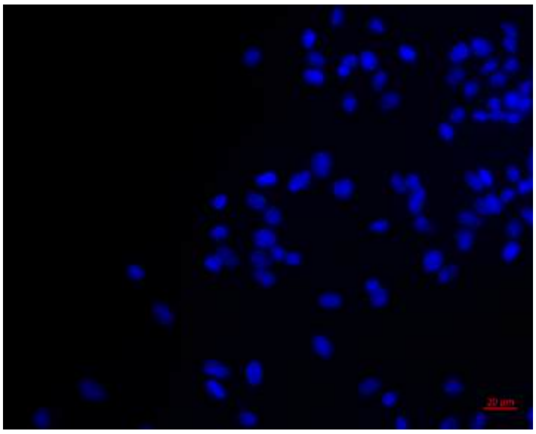

Merge

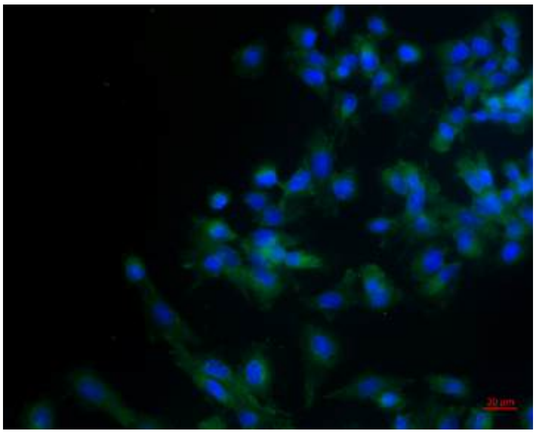

OGD/R

NOX4

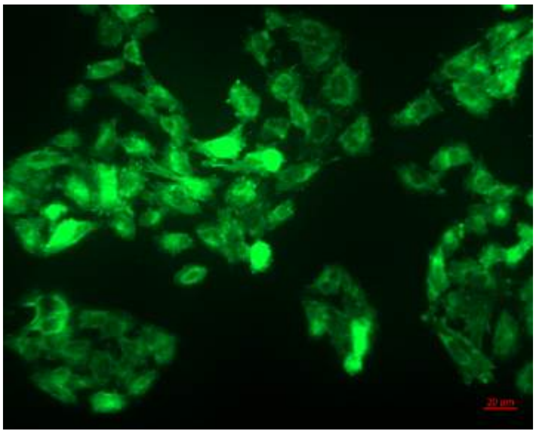

DAPI

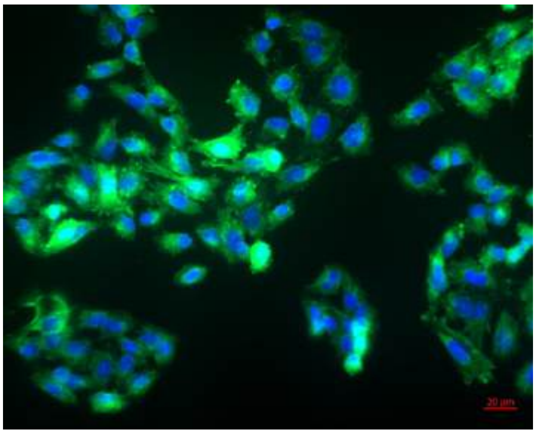

Merge

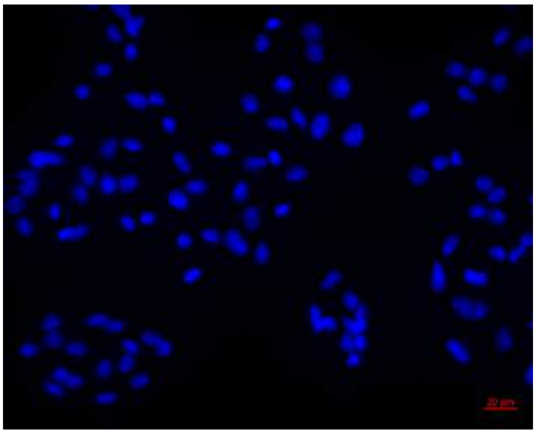

OGD/R + GLI

NOX4

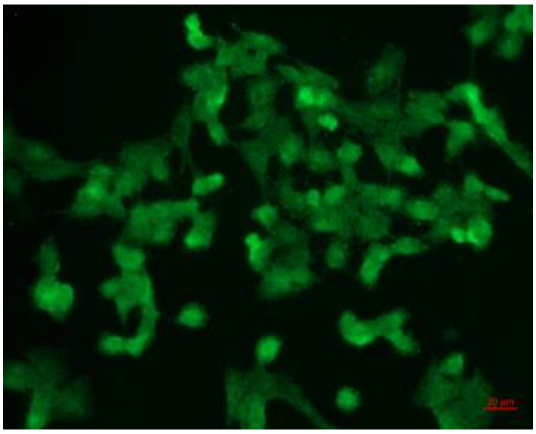

DAPI

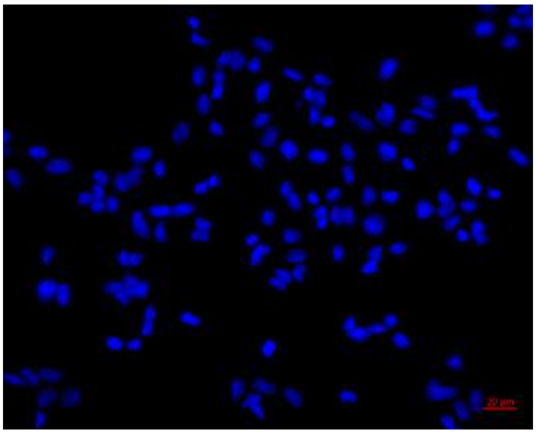

Merge

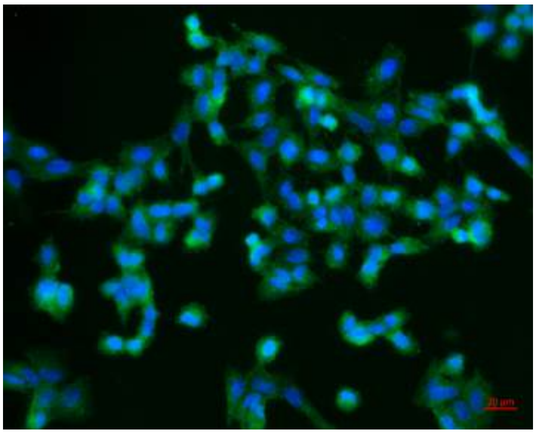

Fig. 5. Representative immunofluorescence images of p-PERK in CTX-TNA2 astrocytes. p-PERK is shown in green, and nuclei are counterstained with DAPI (blue). Scale bars: 20 μm. GLI: glibenclamide.

Control

p-PERK

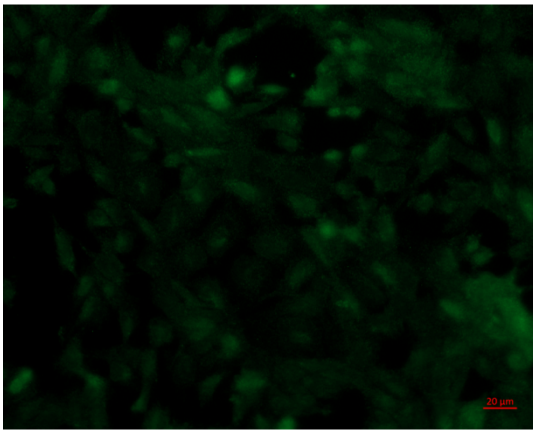

DAPI

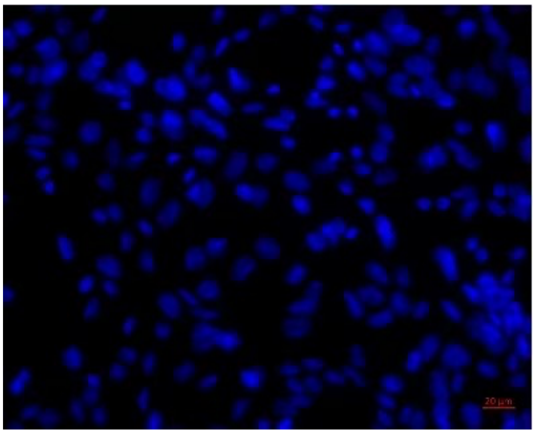

Merge

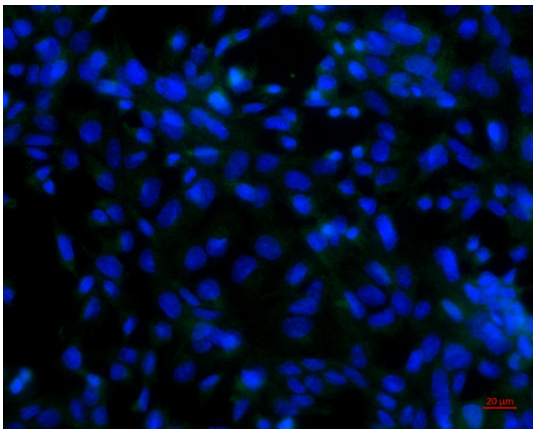

Control +GLI

p-PERK

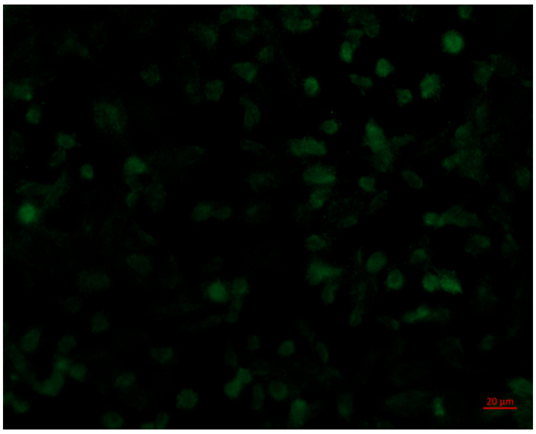

DAPI

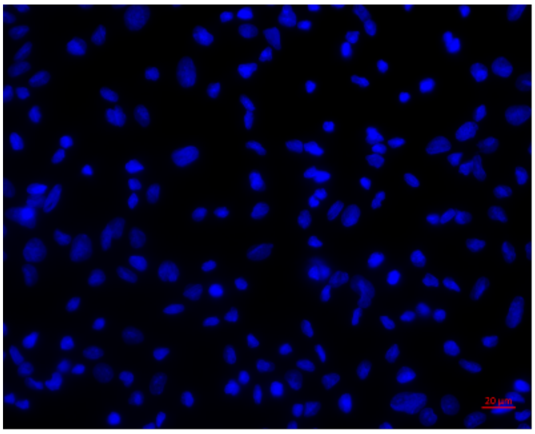

Merge

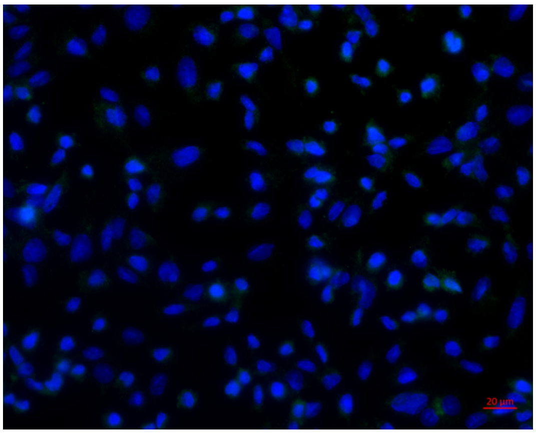

OGD/R

p-PERK

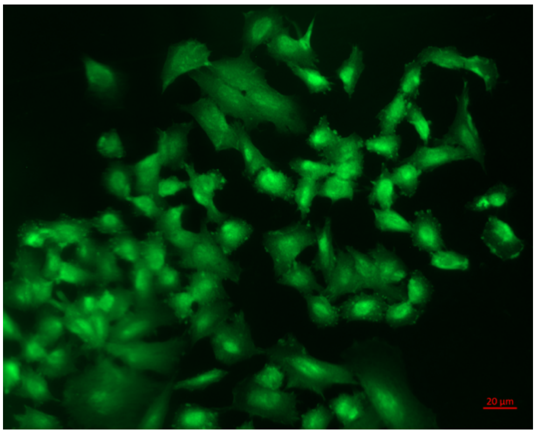

DAPI

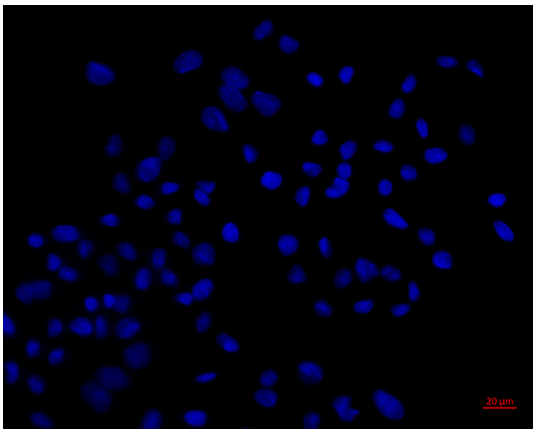

Merge

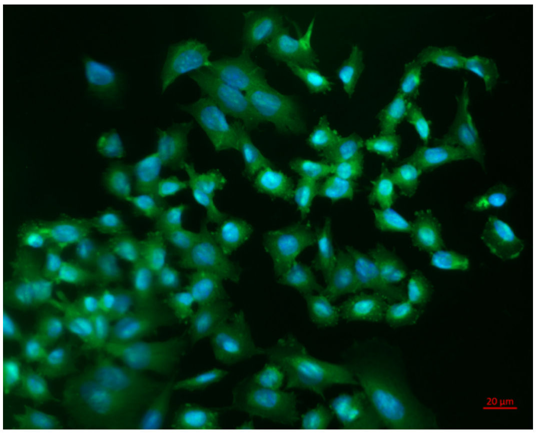

OGD/R + GLI

p-PERK

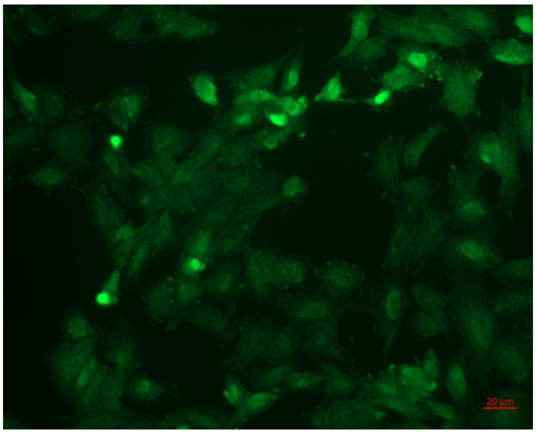

DAPI

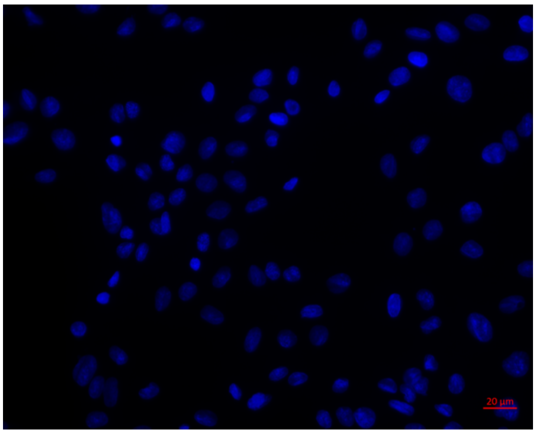

Merge

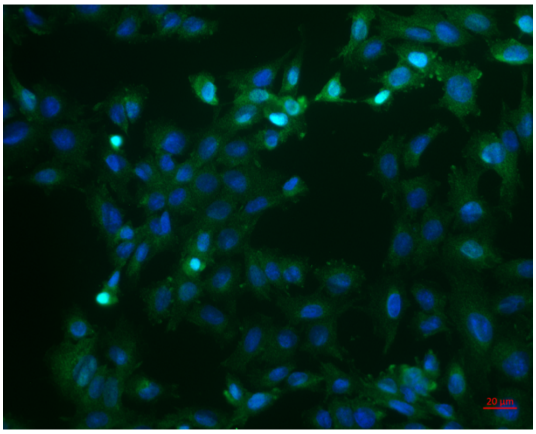

Fig. 7. Representative immunofluorescence images of GFAP in CTX-TNA2 astrocytes. p-PERK is shown in red, and nuclei are counterstained with DAPI (blue). Scale bars: 20 µm. GLI: glibenclamide; GSK: GSK2606414; GKT: GKT137831.

Control

GFAP

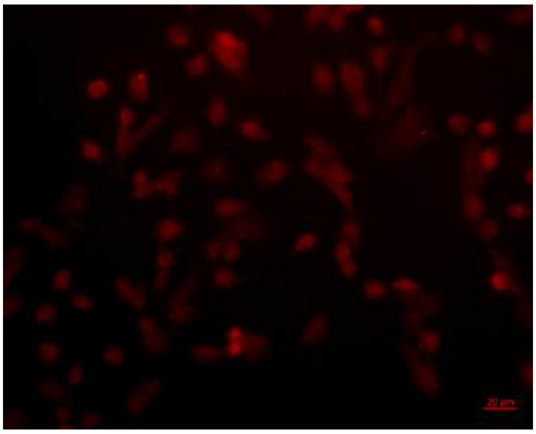

DAPI

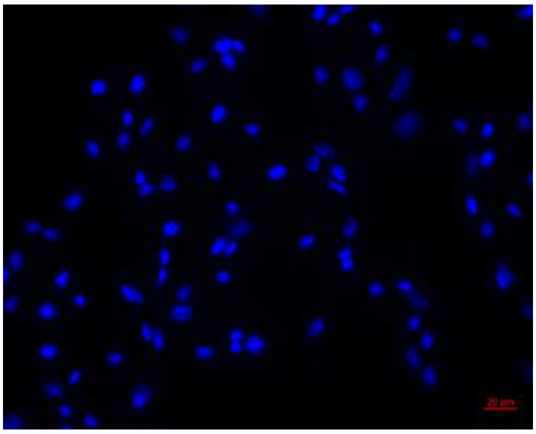

Merge

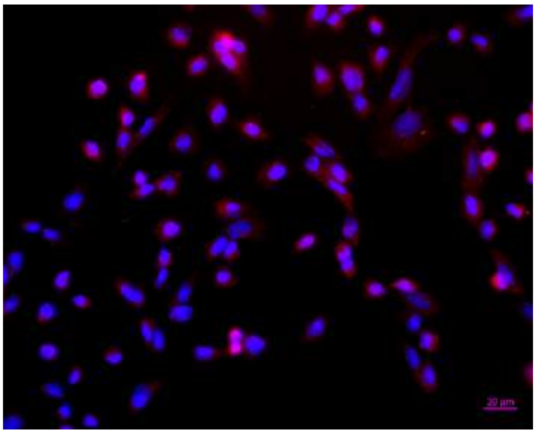

OGD/R

GFAP

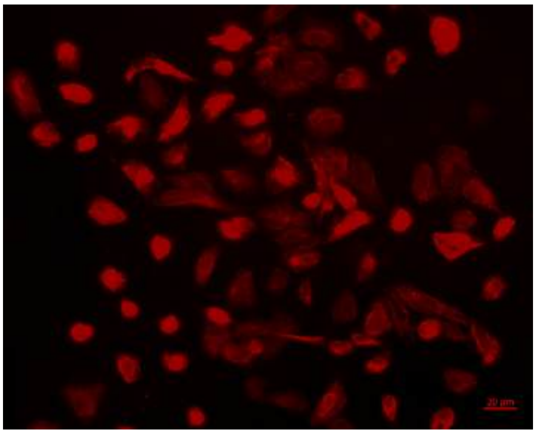

DAPI

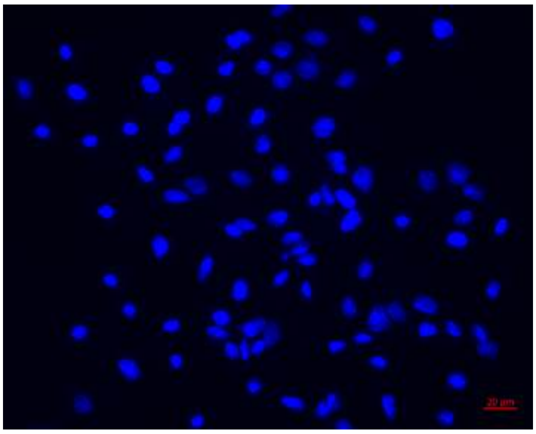

Merge

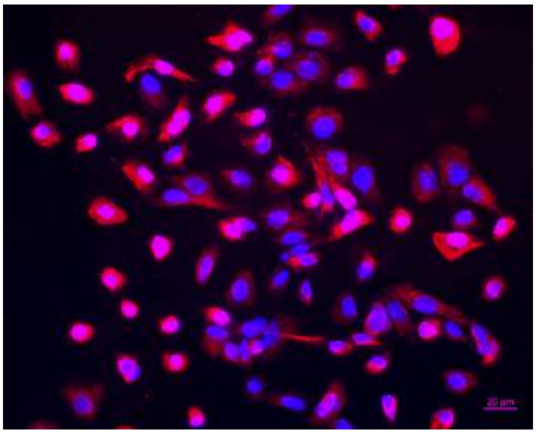

OGD/R + Mannitol

GFAP

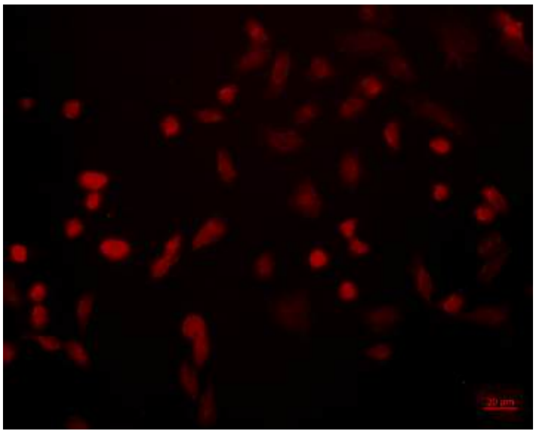

DAPI

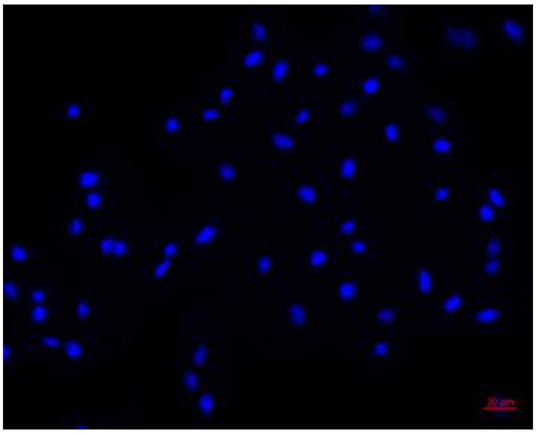

Merge

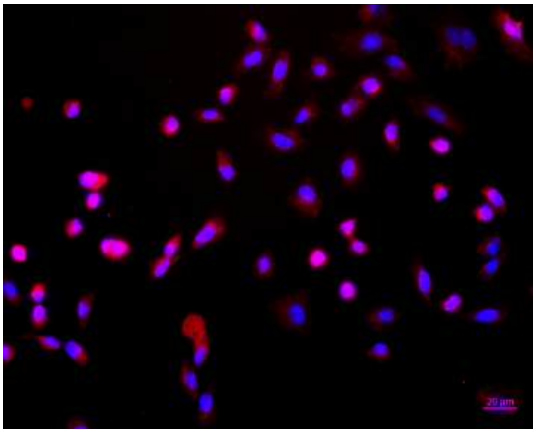

OGD/R + GSK

GFAP

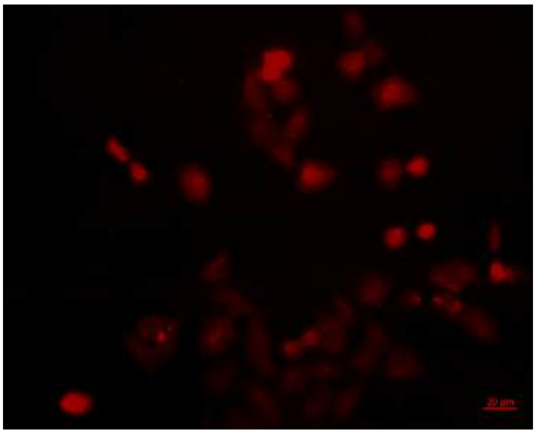

DAPI

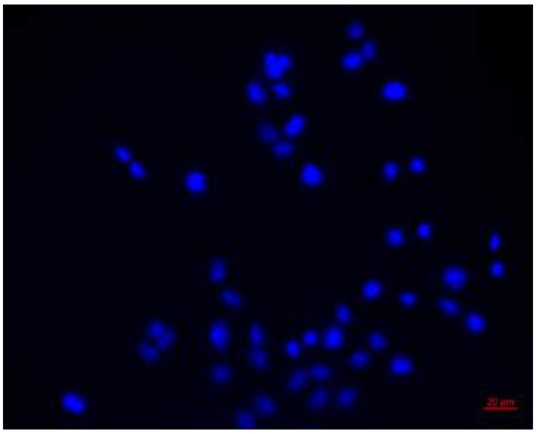

Merge

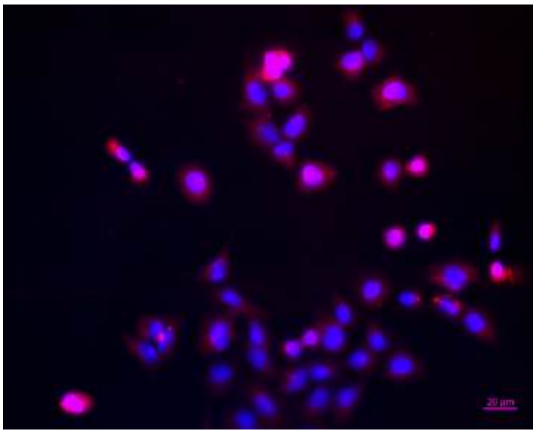

OGD/R + GKT

GFAP

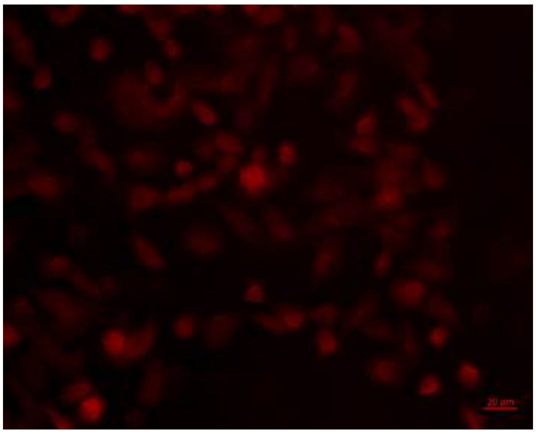

DAPI

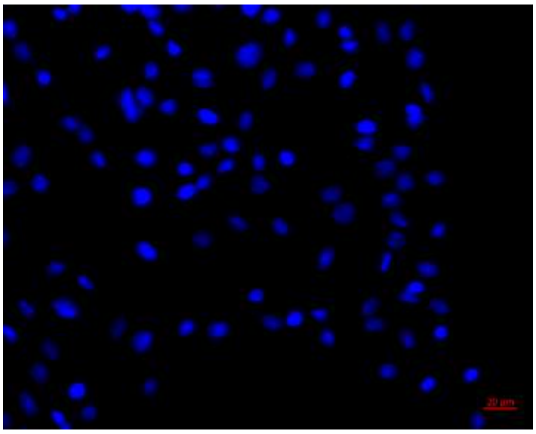

Merge

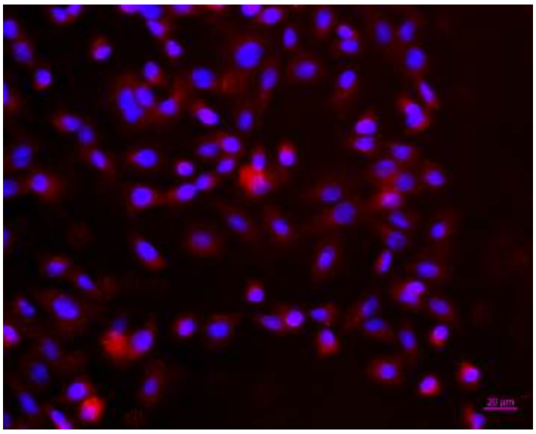

Fig. 7. Representative immunofluorescence images of NOX4 in CTX-TNA2 astrocytes. NOX4 is shown in green, and nuclei are counterstained with DAPI (blue). Scale bars: 20 μm. GLI: glibenclamide; GSK: GSK2606414; GKT: GKT137831.

Control

NOX4

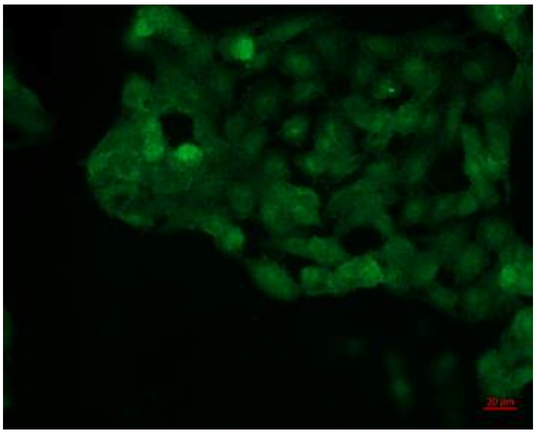

DAPI

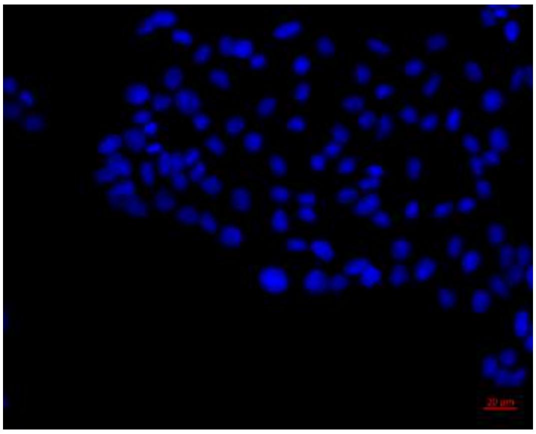

Merge

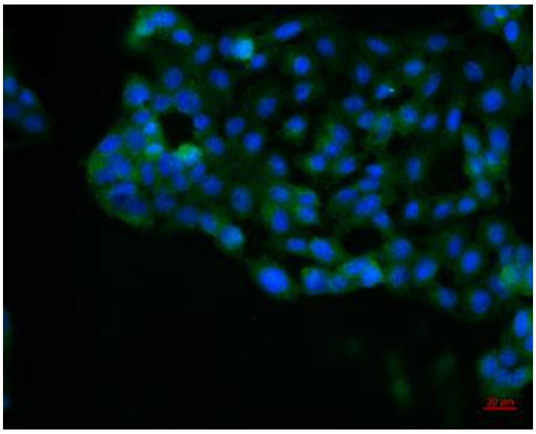

OGD/R

NOX4

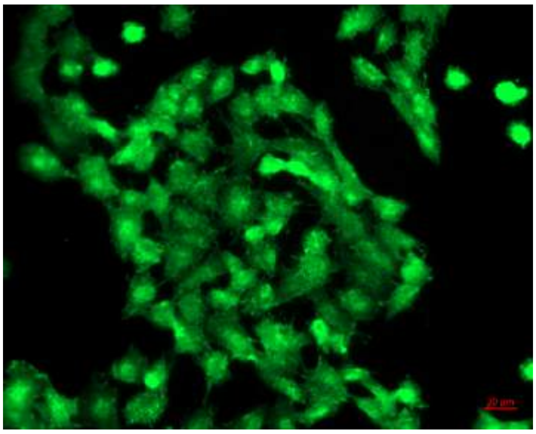

DAPI

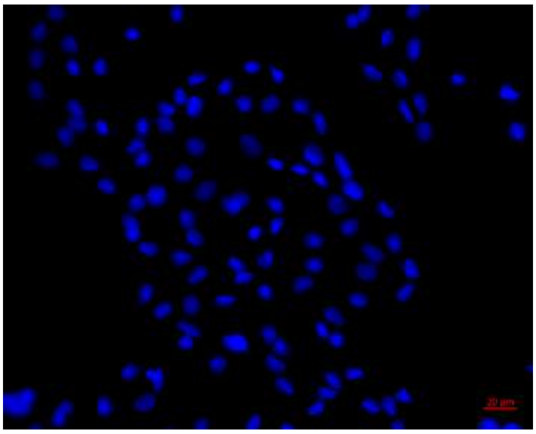

Merge

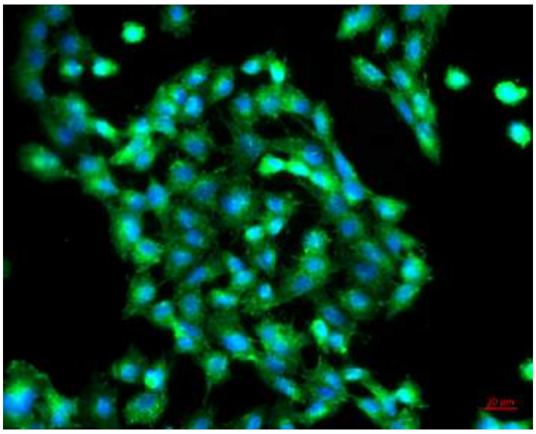

OGD/R + Mannitol

NOX4

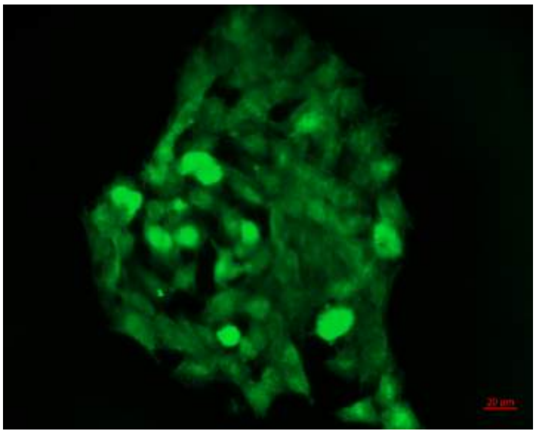

DAPI

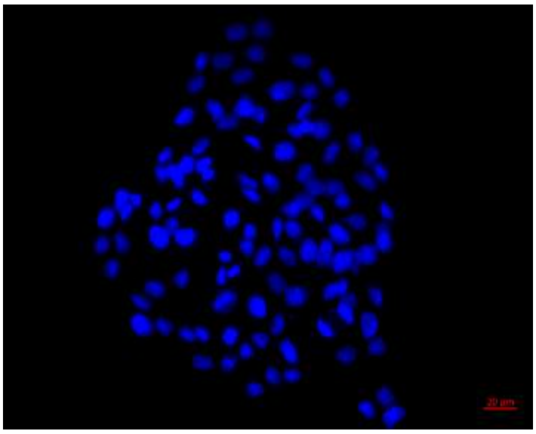

Merge

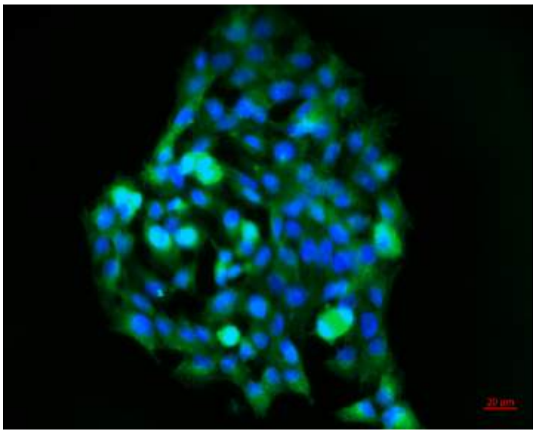

OGD/R + GSK

NOX4

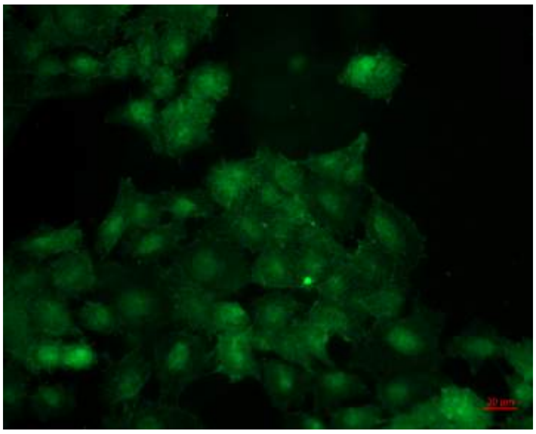

DAPI

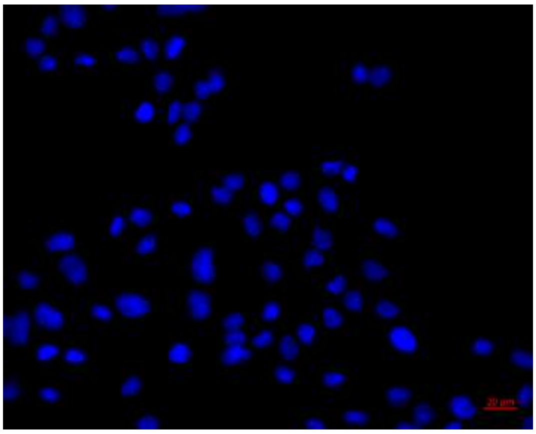

Merge

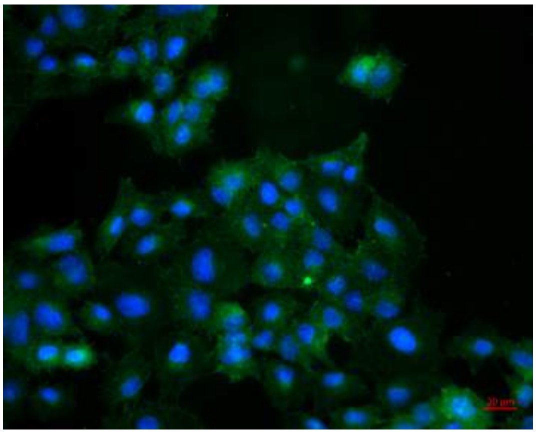

OGD/R + GKT

NOX4

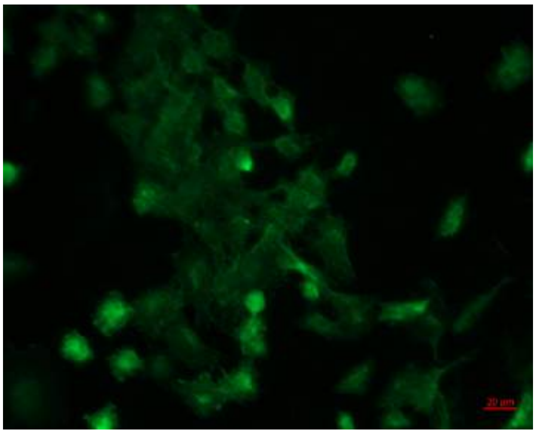

DAPI

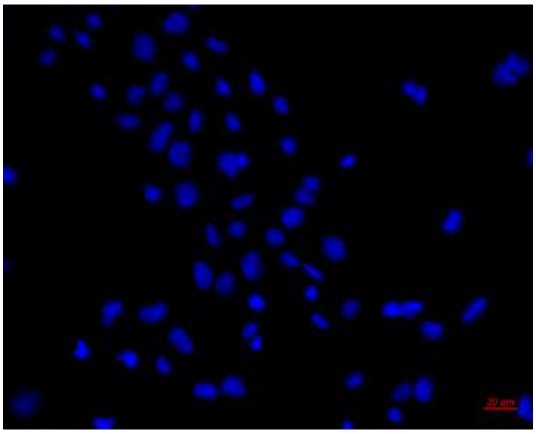

Merge

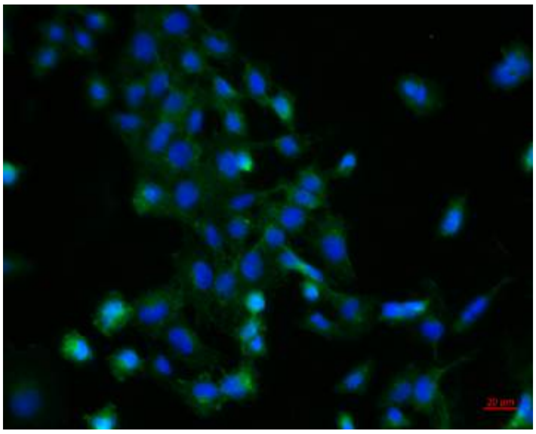

Fig. 7. Representative immunofluorescence images of p-PERK in CTX-TNA2 astrocytes. p-PERK is shown in green, and nuclei are counterstained with DAPI (blue). Scale bars: 20 μm. GLI: glibenclamide; GSK: GSK2606414; GKT: GKT137831.

Control

p-PERK

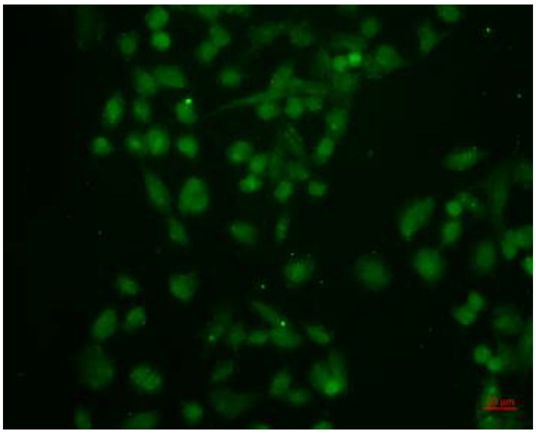

DAPI

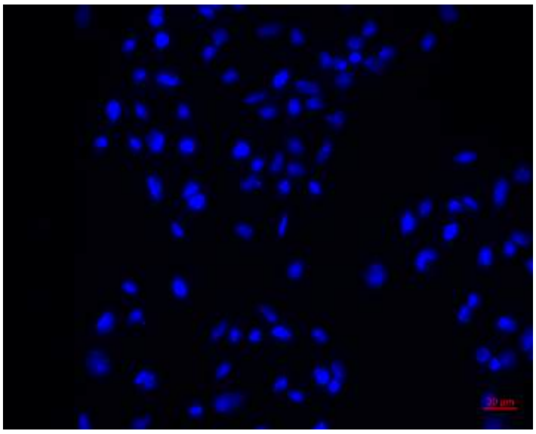

Merge

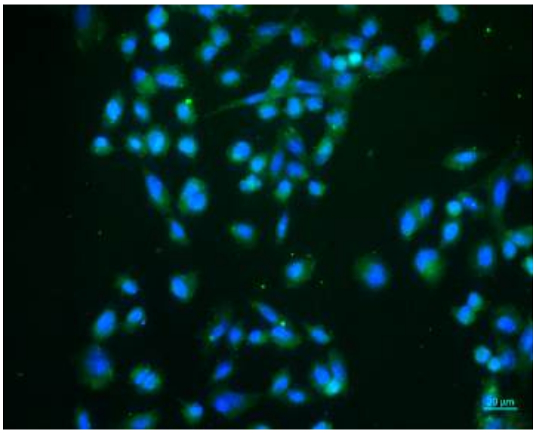

OGD/R

p-PERK

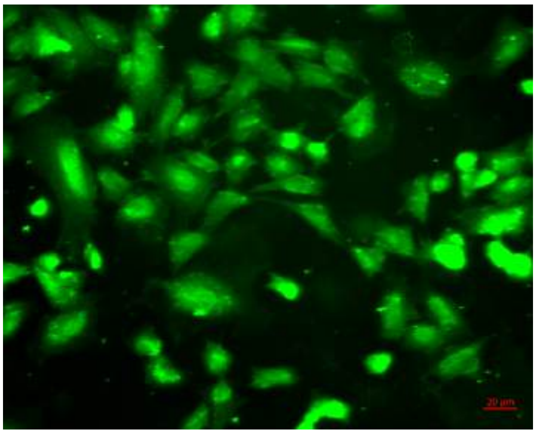

DAPI

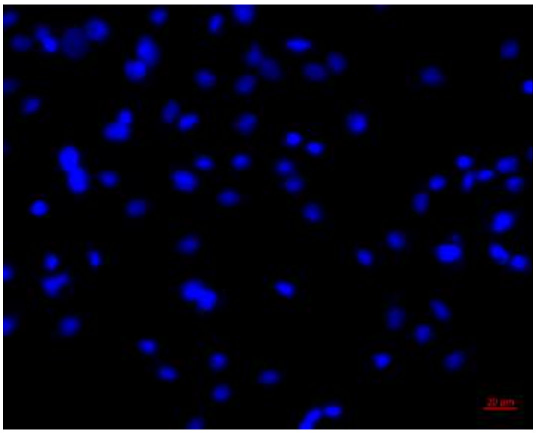

Merge

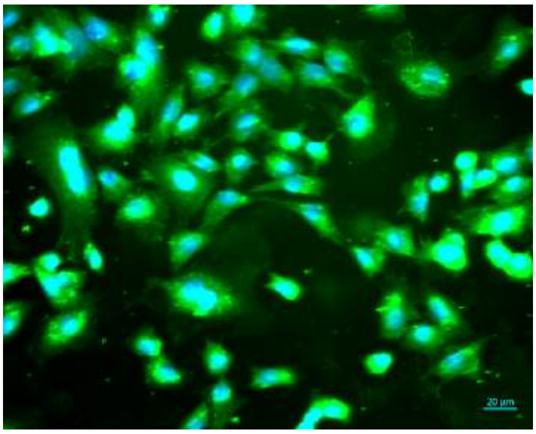

OGD/R + Mannitol

p-PERK

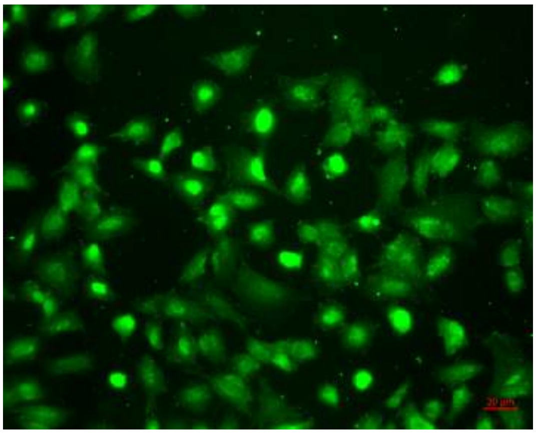

DAPI

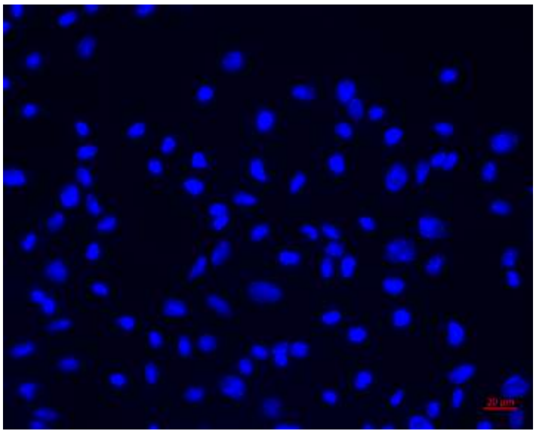

Merge

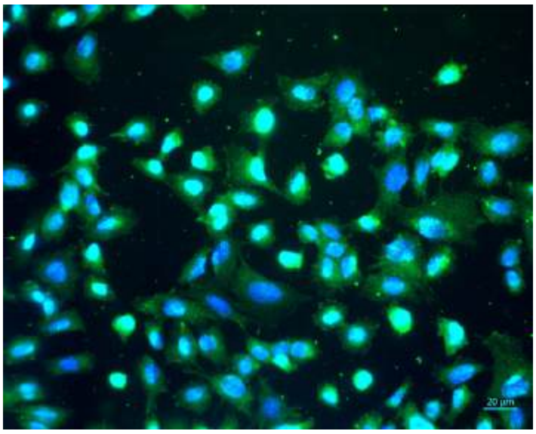

OGD/R + GSK

p-PERK

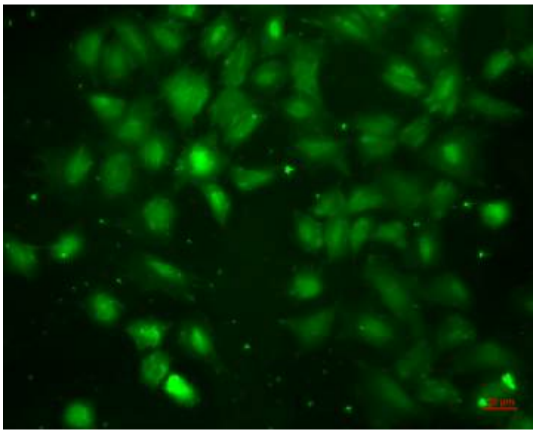

DAPI

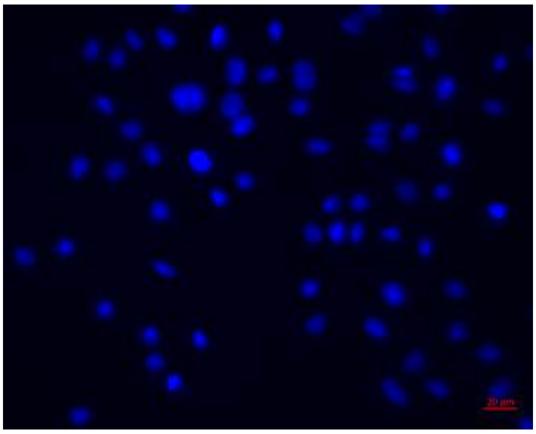

Merge

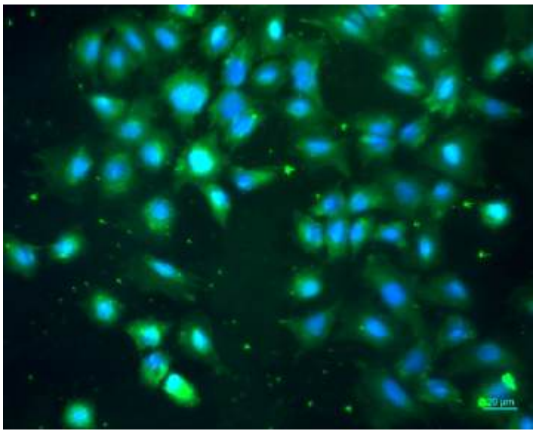

OGD/R + GKT

p-PERK

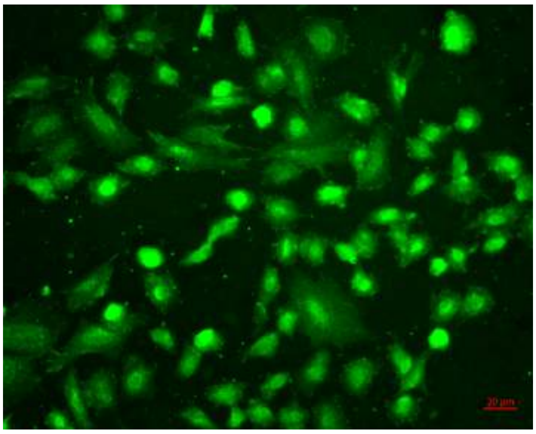

DAPI

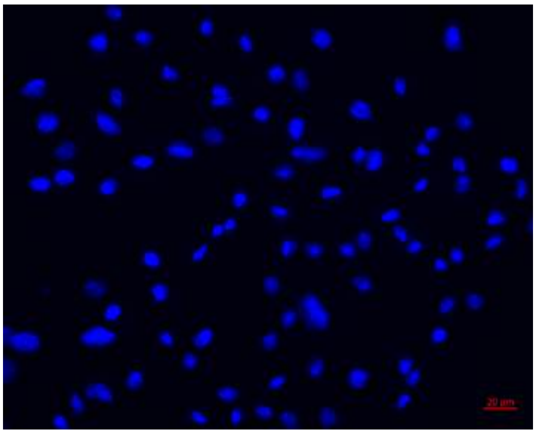

Merge

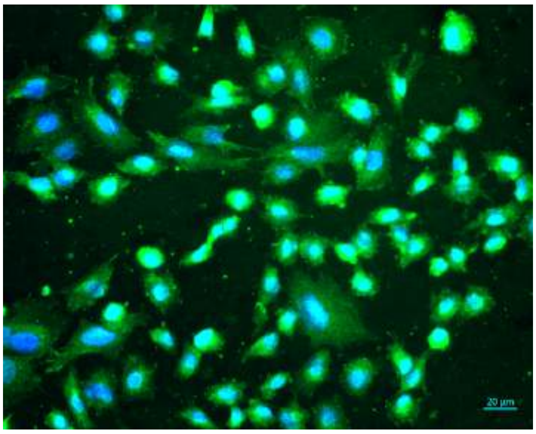

Fig. 8. Representative immunofluorescence images of GFAP in CTX-TNA2 astrocytes treated with astrocyte-conditioned medium (ACM) derived from donor cells under the indicated conditions. GFAP is shown in red, and nuclei are counterstained with DAPI (blue). Scale bars: 20 μm. GLI: glibenclamide.

Control-ACM

GFAP

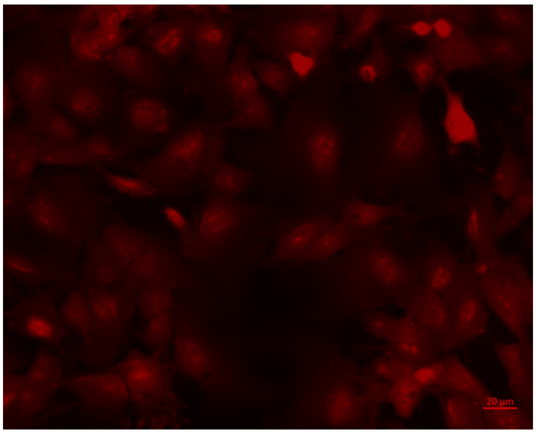

DAPI

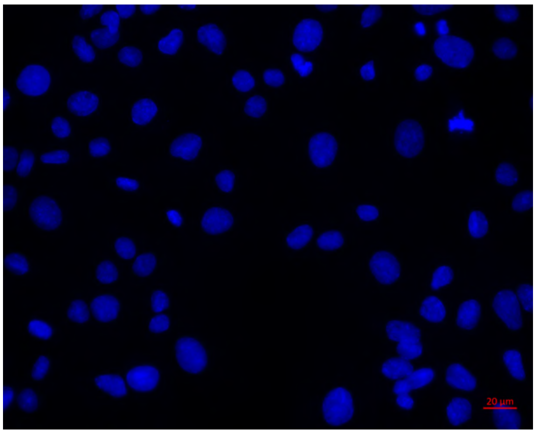

Merge

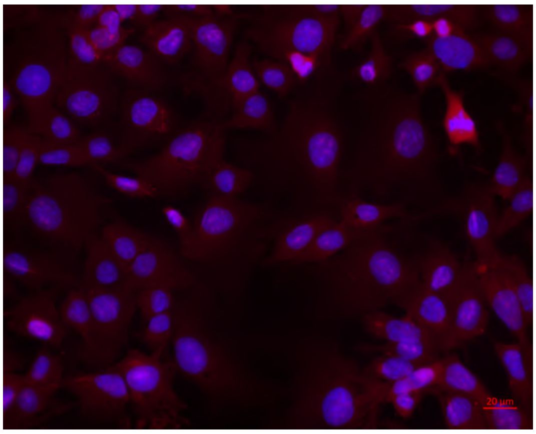

OGD/R-ACM

GFAP

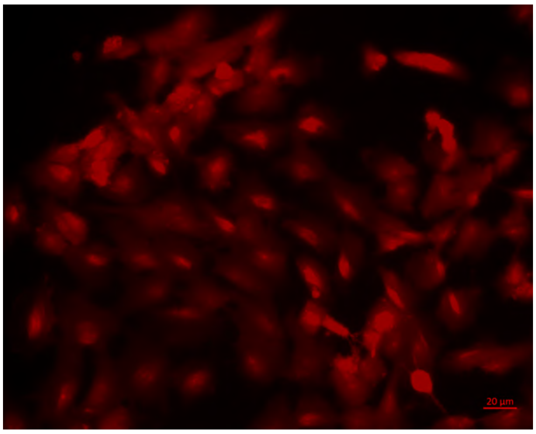

DAPI

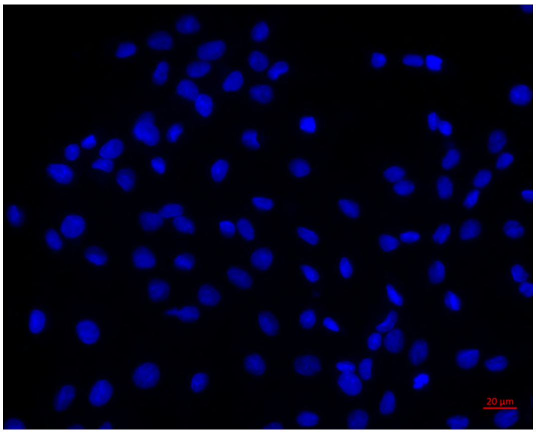

Merge

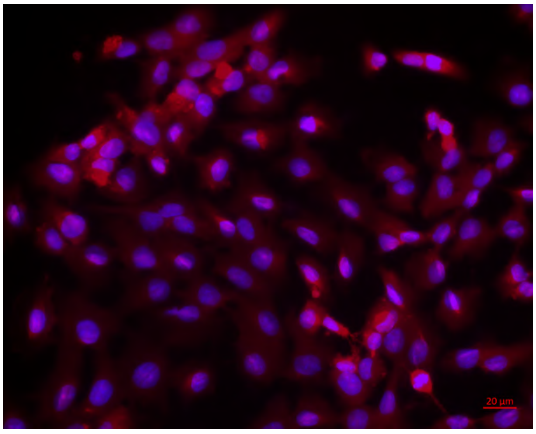

OGD/R+GLI-ACM

GFAP

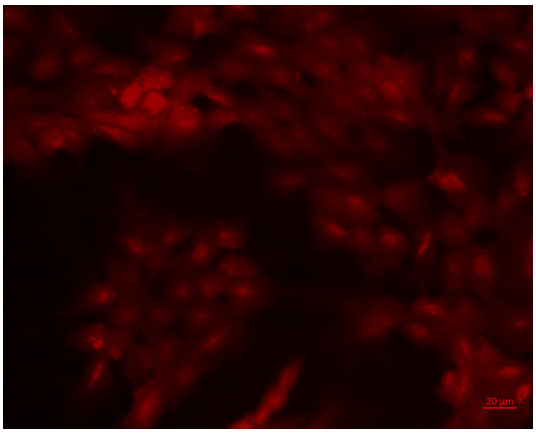

DAPI

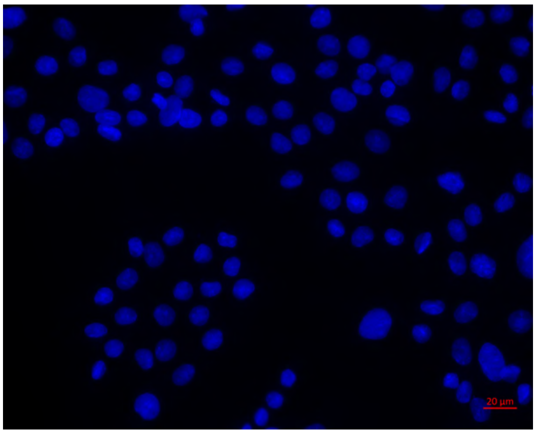

Merge

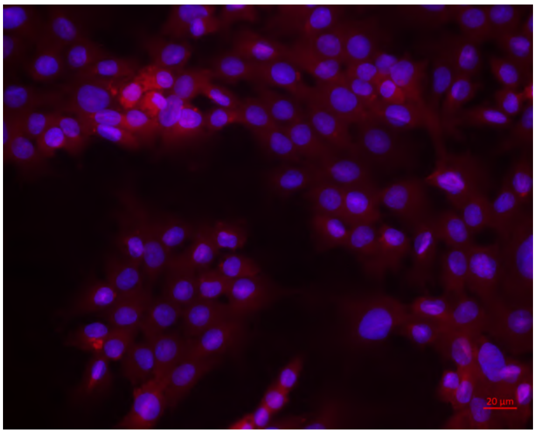

Fig. 8. Representative immunofluorescence images of p-STAT3 in CTX-TNA2 astrocytes treated with astrocyte-conditioned medium (ACM) derived from donor cells under the indicated conditions. p-STAT3 is shown in red, and nuclei are counterstained with DAPI (blue). Scale bars: 20 μm. GLI: glibenclamide.

Control-ACM

p-STAT3

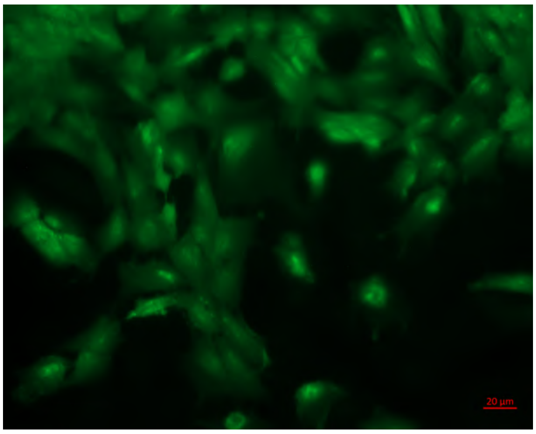

DAPI

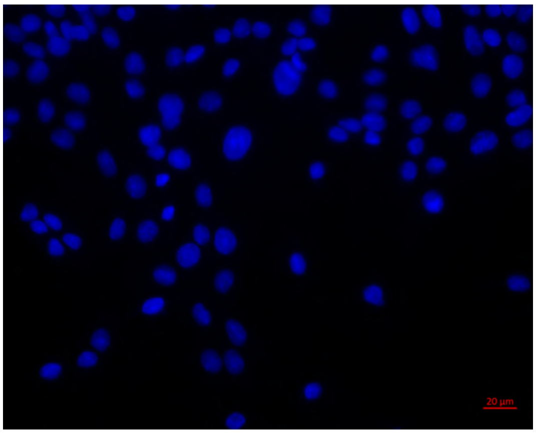

Merge

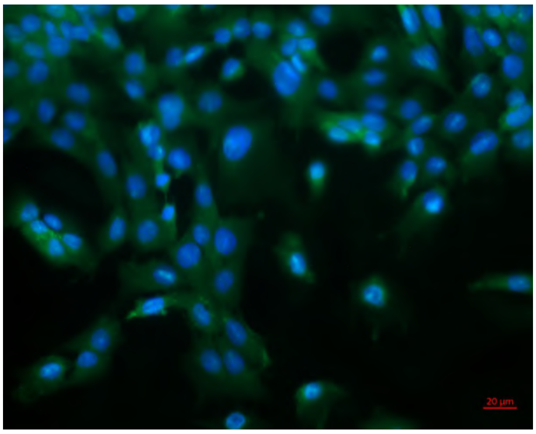

OGD/R-ACM

p-STAT3

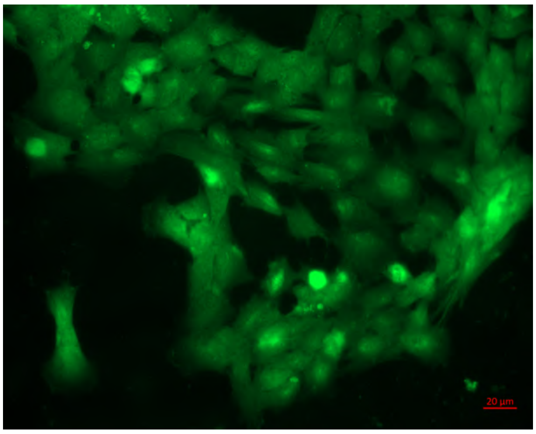

DAPI

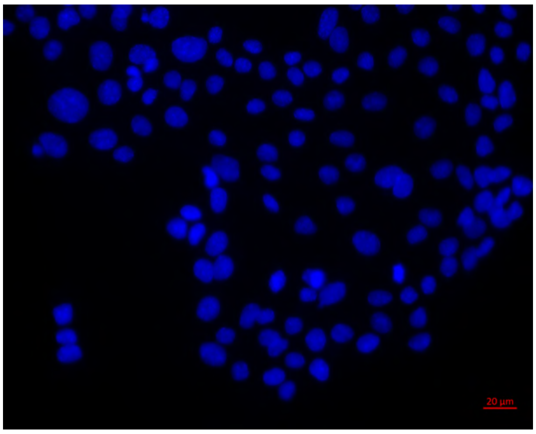

Merge

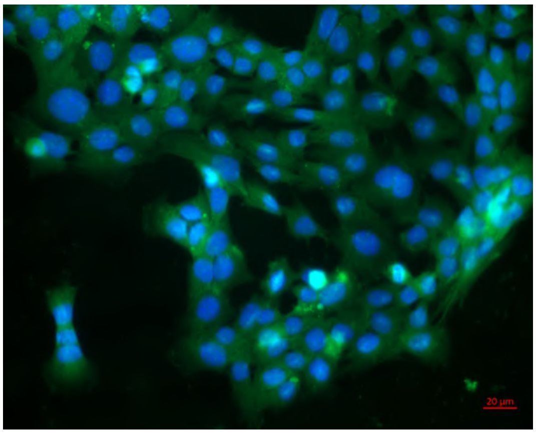

OGD/R+GLI-ACM

p-STAT3

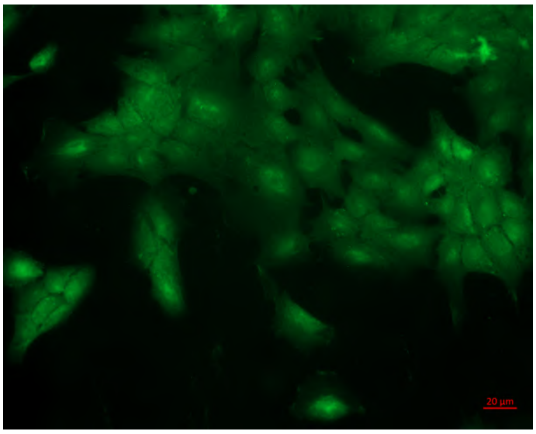

DAPI

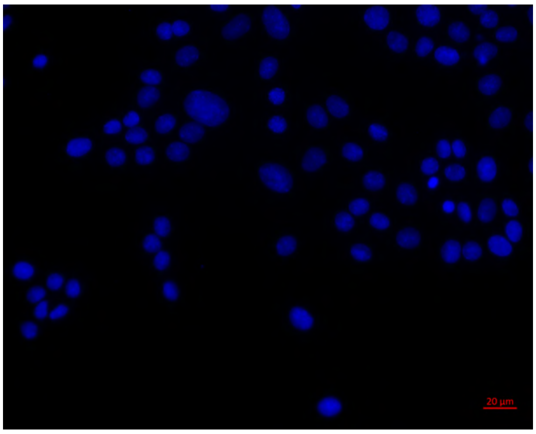

Merge

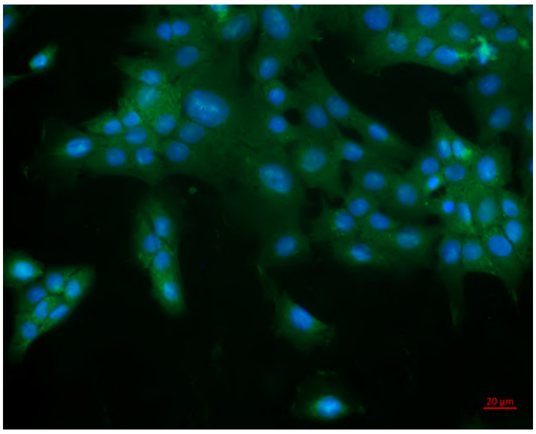

Part IV. Western blot raw images

Fig. 3C. Images were acquired from the same membrane using **ECL** for signal detection and **bright-field** imaging for membrane visualization. Lanes are labeled as 1–4 corresponding to the experimental groups: (1) Control, (2) Control + GLI, (3) OGD/R, and (4) OGD/R + GLI. Lanes labeled as "**X**" represent lanes not included in the final analysis but are shown here for full transparency of the original membrane.

GFAP

(A) Bright-field image

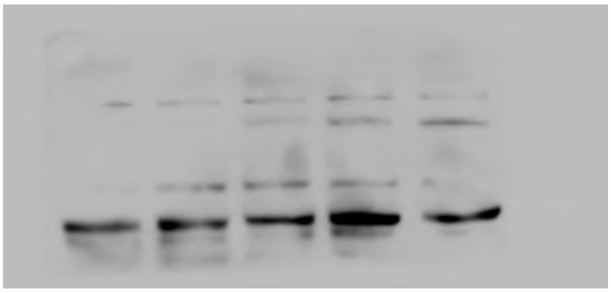

(B) Corresponding bright-field image

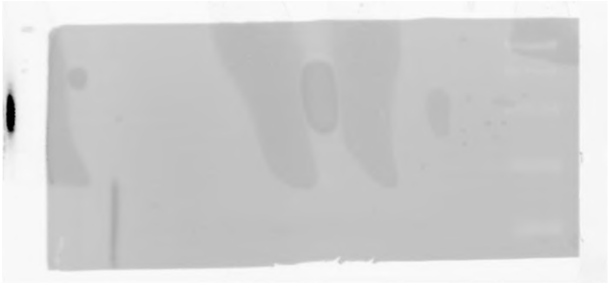

(C) Composite image (for molecular weight comparison only)

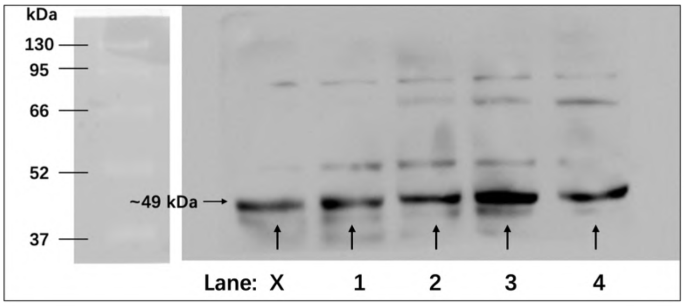

β-actin

(D) Bright-field image

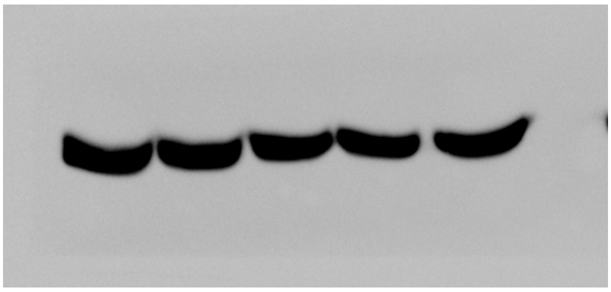

(E) Corresponding bright-field image

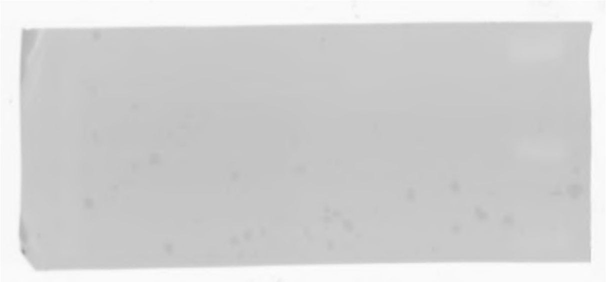

(C) Composite image (for molecular weight comparison only)

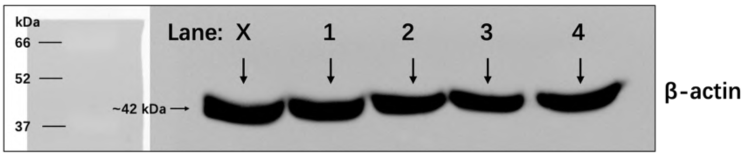

Fig. 4B. Images were acquired from the same membrane using **ECL** for signal detection and **bright-field** imaging for membrane visualization. Lanes are labeled as 1–4 corresponding to the experimental groups: (1) Control, (2) Control + GLI, (3) OGD/R, and (4) OGD/R + GLI. Lanes labeled as "**X**" represent lanes not included in the final analysis but are shown here for full transparency of the original membrane.

NOX4

(A) Bright-field image

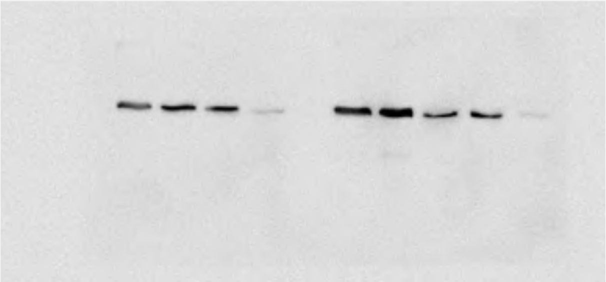

(B)ECL image-NOX4

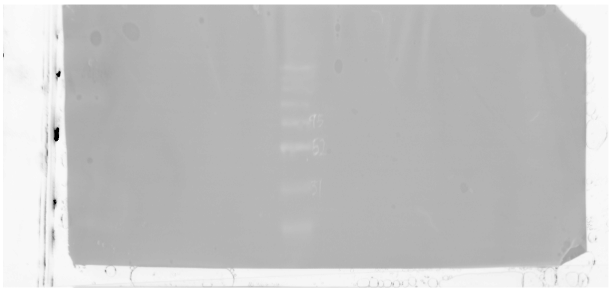

(C) Composite image (for molecular weight comparison only).

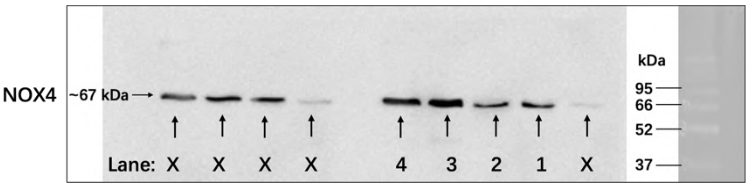

β-actin

(D) ECL image-β-actin. Images were derived from the same membrane.

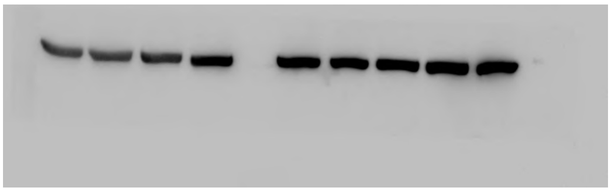

(E) ECL image-β-actin. Images were derived from the same membrane.

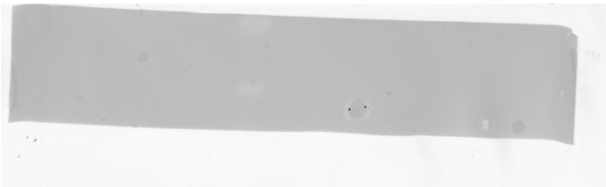

(F) Composite image (for molecular weight comparison only)

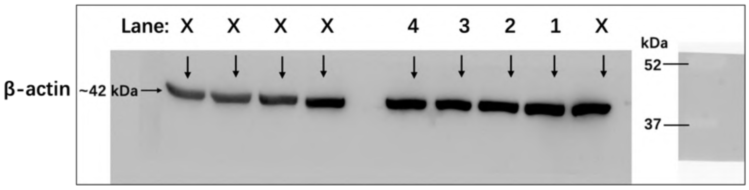

Note: The horizontal flipping was applied uniformly to the entire image and does not alter the relative band positions or intensities for Fig 4B.

Fig. 6A. Images were acquired from the same membrane using **ECL** for signal detection and **bright-field** imaging for membrane visualization. Lanes are labeled as 1–4 corresponding to the experimental groups: (1) Control, (2) Control + GLI, (3) OGD/R, and (4) OGD/R + GLI. Lanes labeled as "**X**" represent lanes not included in the final analysis but are shown here for full transparency of the original membrane.

p-STAT3

(A) ECL image

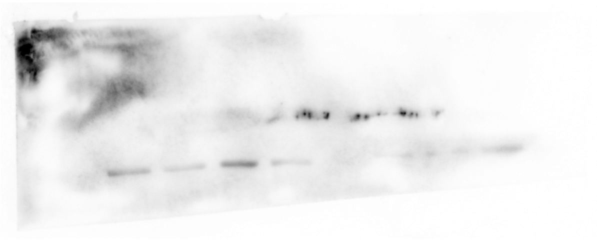

(B) Bright-field image

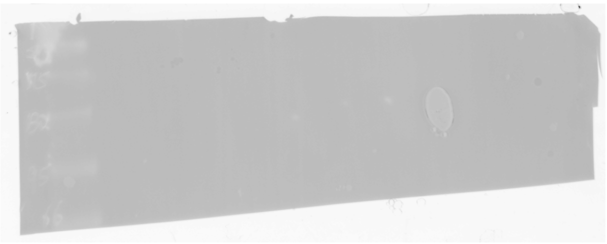

(C) Composite image (for molecular weight comparison only)

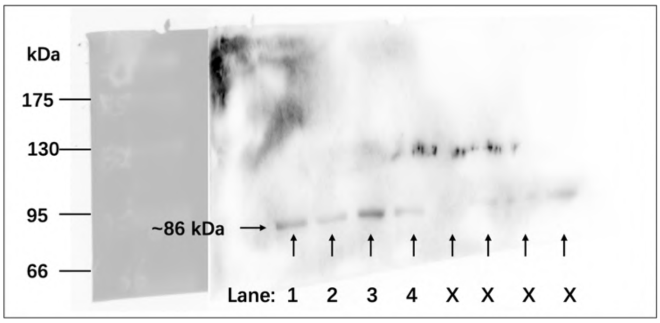

β-actin

(D) ECL image

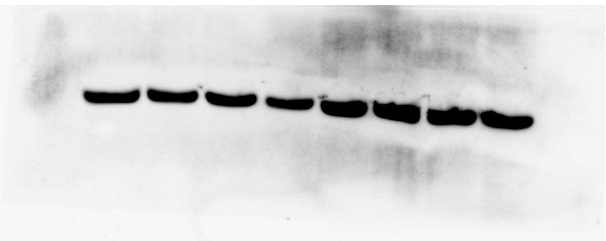

(E) Bright-field image

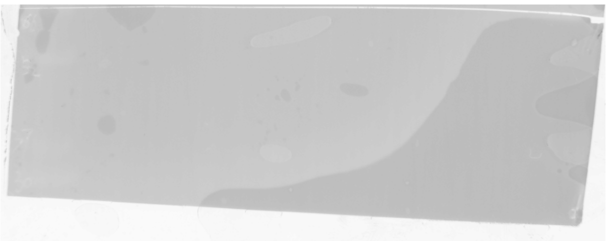

(F) Composite image (for molecular weight comparison only)

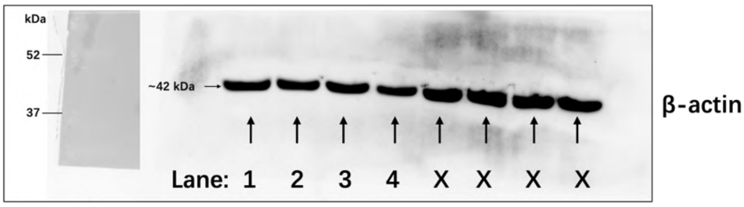

Fig. 6A. Images were acquired from the same membrane using **ECL** for signal detection and **bright-field** imaging for membrane visualization. Lanes are labeled as 1–4 corresponding to the experimental groups: (1) Control, (2) Control + GLI, (3) OGD/R, and (4) OGD/R + GLI. Lanes labeled as "**X**" represent lanes not included in the final analysis but are shown here for full transparency of the original membrane.

STAT3 and β-actin

(A) ECL image

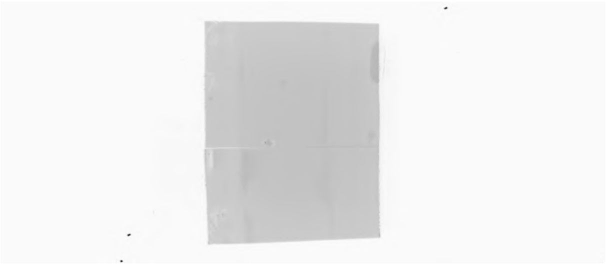

(B) Bright-field image

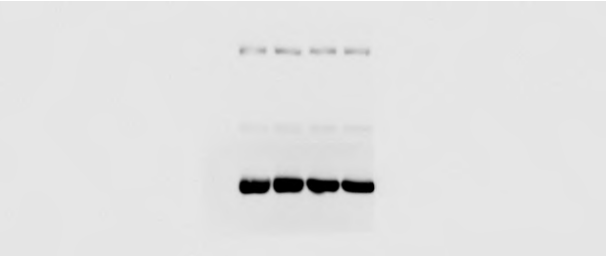

(C) Composite image (for molecular weight comparison only)

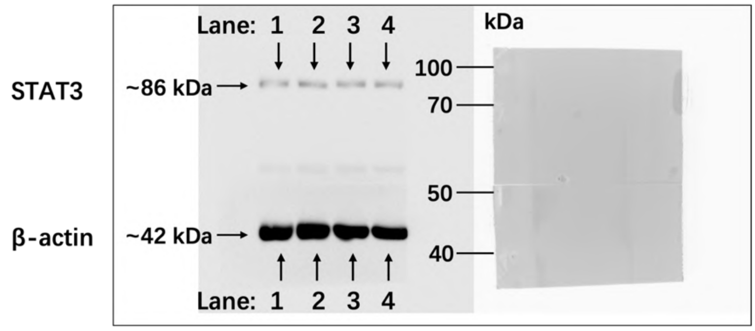

Note: The p-STAT3/STAT3 ratio was determined by normalizing p-STAT3 and STAT3 to β-actin, respectively, and then calculating the ratio between the normalized values.
Brightness and contrast were adjusted uniformly in the figures presented in the main text to improve visualization without altering the original signal.
