# Supplementary material for: Inhibition of SUR1-TRPM4 attenuates astrocyte swelling and reactivity under oxygen-glucose deprivation/reoxygenation
Source: PLoS One. 2026 Jun 24;21(6):e0352151. doi: 10.1371/journal.pone.0352151 (PMC13293404; doi:10.1371/journal.pone.0352151)
Supplement: Attachment — Submitted filename: Response to Reviewers.pdf [file pone.0352151.s004.pdf]

Sompol Tapechum, M.D., Ph.D.
Assistant Professor
Department of Physiology
Faculty of Medicine Siriraj Hospital
Mahidol University, Bangkok, Thailand.
Tel. +662-419-9723
Sompol.tap@mahidol.ac.th

Alexander A. Mongin, Ph.D.
Academic Editor
PLOS One

March 30, 2026

Dear Editors,

Thank you very much for your valuable comments and constructive suggestions. we consider all of them to be highly important and helpful in improving the quality of our manuscript. Below, we provide a detailed, point-by-point response, along with the corresponding revisions made in accordance with the reviewers' comments and recommendations. The responses for the comments are in red texts.

PONE-D-26-12414
Inhibition of SUR1–TRPM4 attenuates astrocyte swelling and reactivity under oxygen-glucose deprivation/reoxygenation
PLOS One
Dear Dr. Tapechum,

Thank you for submitting your manuscript to PLOS ONE. During preliminary evaluation of your manuscript, I have found that it has merit but does not fully meet PLOS ONE's publication criteria as it currently stands. Therefore, we invite you to submit a revised version of the manuscript that addresses the points raised during the editorial screening process.

Specifically:

During the preliminary evaluation of your manuscript, I noted that the submission does not comply with the PLOS ONE Policy on Data Availability. As stated in the journal's guidelines:

"PLOS journals require authors to make all data necessary to replicate their study's findings publicly available without restriction at the time of publication."

Compliance can be achieved by either:
(a) Providing all critical primary data and unedited images (e.g., full-length western blots, uncropped ICC and EM images) as supplemental files; or
(b) Depositing all critical primary data and unedited primary images in an appropriate public repository.

Please note that any embargoed data must still be made available for inspection during the peer review process. Failure to meet this requirement will result in rejection of the manuscript without further review.

Thank you for the suggestion, we consider the PLOS ONE Policy on Data Availability is very important. We added two Supporting Information files with this revised manuscript.

1. "S1_Raw_data" is the excel file containing the raw data of the results in this manuscript.
2. "S1_Raw_image" contains the original western blot and immunofluorescent images used in this manuscript.

In addition, I strongly recommend including individual data points (scatter plots) overlaid on all bar graphs to improve transparency and enhance the rigor of data presentation.

Thank you for the suggestion, we added individual data points (scatter plots) overlaid on all bar graphs to improve transparency and enhance the rigor of data presentation.

Please submit your revised manuscript by May 01 2026 11:59PM. If you will need more time than this to complete your revisions, please reply to this message or contact the journal office at plosone@plos.org. When you're ready to submit your revision, log on to https://www.editorialmanager.com/pone/ and select the 'Submissions Needing Revision' folder to locate your manuscript file.

Please include the following items when submitting your revised manuscript:
A letter that responds to each point raised by the academic editor and reviewer(s). You should upload this letter as a separate file labeled 'Response to Reviewers'.
A marked-up copy of your manuscript that highlights changes made to the original version. You should upload this as a separate file labeled 'Revised Manuscript with Track Changes'.
An unmarked version of your revised paper without tracked changes. You should upload this as a separate file labeled 'Manuscript'.

Thank you for the suggestion, we provided this 'Response to Reviewers' as you recommended.
We also included a marked-up copy of the manuscript that highlights changes made to the original version in a file name 'Revised Manuscript with Track Changes' and an unmarked version of the revised manuscript without tracked changes in a file name 'Manuscript'.

If you would like to make changes to your financial disclosure, please include your updated statement in your cover letter. Guidelines for resubmitting your figure files are available below the reviewer comments at the end of this letter.

Thank you for the suggestion. We included the updated statement in the cover letter.

If applicable, we recommend that you deposit your laboratory protocols in protocols.io to enhance the reproducibility of your results. Protocols.io assigns your protocol its own identifier (DOI) so that it can be cited independently in the future. For instructions see: https://journals.plos.org/plosone/s/submission-guidelines#loc-laboratory-protocols. Additionally, PLOS ONE offers an option for publishing peer-reviewed Lab Protocol articles, which describe protocols hosted on protocols.io. Read more information on sharing protocols at https://plos.org/protocols?utm_medium=editorial-email&utm_source=authorletters&utm_campaign=protocols.

Thank you for the suggestion. We decided to add two supporting files containing all raw data in comply with PLOS ONE Policy on Data Availability. The two supporting files are those mentioned above, "S1_Raw_data" and "S1_Raw_image".

We look forward to receiving your revised manuscript.

Kind regards,

Alexander A. Mongin, Ph.D.
Academic Editor
PLOS One

Journal Requirements:

When submitting your revision, we need you to address these additional requirements.

1. Please ensure that your manuscript meets PLOS ONE's style requirements, including those for file naming. The PLOS ONE style templates can be found at https://journals.plos.org/plosone/s/file?id=wjVg/PLOSOne_formatting_sample_main_body.pdf and https://journals.plos.org/plosone/s/file?id=ba62/PLOSOne_formatting_sample_title_authors_affiliations.pdf

Thank you for the suggestion. We have revised our manuscript to meet PLOS ONE's style requirements including the file naming. We also rearranged the manuscript writing and correction of typographic errors for the clarity of the manuscript while still maintained the integrity of our work.

2. Thank you for stating in your Funding Statement:
"This research was supported by Siriraj Research and Development Fund from the Faculty of Siriraj Hospital, Mahidol University, Thailand, to ST under the grant NO. R016732027. The funder did not play any role in the study design, data collection and analysis, decision to publish, or preparation of the manuscript.

URL: https://si.mahidol.ac.th/th/research-academics/research/fund_research.html"

Please provide an amended statement that declares *all* the funding or sources of support (whether external or internal to your organization) received during this study, as detailed online in our guide for authors at http://journals.plos.org/plosone/s/submit-now. Please also include the statement "There was no additional external funding received for this study." in your updated Funding Statement.
Please include your amended Funding Statement within your cover letter. We will change the online submission form on your behalf.

Thank you for the suggestion. We have updated the funding statement by adding "There was no additional external funding received for this study." online and also declared in the cover letter.

3. Thank you for stating the following financial disclosure:
"This research was supported by Siriraj Research and Development Fund from the Faculty of Siriraj Hospital, Mahidol University, Thailand, to ST under the grant NO. R016732027. The funder did not play any role in the study design, data collection and analysis, decision to publish, or preparation of the manuscript.

URL: https://si.mahidol.ac.th/th/research-academics/research/fund_research.html"

We note that one or more of the authors is affiliated with the funding organization, indicating the funder may have had some role in the design, data collection, analysis or preparation of your

manuscript for publication; in other words, the funder played an indirect role through the participation of the co-authors. If the funding organization did not play a role in the study design, data collection and analysis, decision to publish, or preparation of the manuscript and only provided financial support in the form of authors' salaries and/or research materials, please do the following:

a. Review your statements relating to the author contributions, and ensure you have specifically and accurately indicated the role(s) that these authors had in your study. These amendments should be made in the online form.

Thank you for the suggestion. We have reviewed our statements regarding the author contributions and affirm that all the information provided is accurate.

b. Confirm in your cover letter that you agree with the following statement, and we will change the online submission form on your behalf:
"The funder provided support in the form of salaries for authors [insert relevant initials], but did not have any additional role in the study design, data collection and analysis, decision to publish, or preparation of the manuscript. The specific roles of these authors are articulated in the 'author contributions' section.

Thank you for the suggestion. We have confirmed in the cover letter that we agreed with the following statement.
"The funder provided support in the form of salaries for authors [NP, RS, UV, ST], but did not have any additional role in the study design, data collection and analysis, decision to publish, or preparation of the manuscript. The specific roles of these authors are articulated in the 'author contributions' section."

4. We note that your Data Availability Statement is currently as follows: All relevant data are within the manuscript and its Supporting Information files.

Please confirm at this time whether or not your submission contains all raw data required to replicate the results of your study. Authors must share the "minimal data set" for their submission. PLOS defines the minimal data set to consist of the data required to replicate all study findings reported in the article, as well as related metadata and methods (https://journals.plos.org/plosone/s/data-availability#loc-minimal-data-set-definition).

For example, authors should submit the following data:

- The values behind the means, standard deviations and other measures reported;
- The values used to build graphs;
- The points extracted from images for analysis.

Authors do not need to submit their entire data set if only a portion of the data was used in the reported study.

If your submission does not contain these data, please either upload them as Supporting Information files or deposit them to a stable, public repository and provide us with the relevant URLs, DOIs, or accession numbers. For a list of recommended repositories, please see https://journals.plos.org/plosone/s/recommended-repositories.

If there are ethical or legal restrictions on sharing a de-identified data set, please explain them in detail (e.g., data contain potentially sensitive information, data are owned by a third-party organization, etc.) and who has imposed them (e.g., an ethics committee). Please also provide contact information for a data access committee, ethics committee, or other institutional body to which data requests may be sent. If data are owned by a third party, please indicate how others may request data access.

Thank you for the suggestion. We have added all relevant data in the manuscript and the two Supporting Information files to comply with the above statement.

5. PLOS ONE now requires that authors provide the original uncropped and unadjusted images underlying all blot or gel results reported in a submission's figures or Supporting Information files. This policy and the journal's other requirements for blot/gel reporting and figure preparation are described in detail at https://journals.plos.org/plosone/s/figures#loc-blot-and-gel-reporting-requirements and https://journals.plos.org/plosone/s/figures#loc-preparing-figures-from-image-files. When you submit your revised manuscript, please ensure that your figures adhere fully to these guidelines and provide the original underlying images for all blot or gel data reported in your submission. See the following link for instructions on providing the original image data: https://journals.plos.org/plosone/s/figures#loc-original-images-for-blots-and-gels.
In your cover letter, please note whether your blot/gel image data are in Supporting Information or posted at a public data repository, provide the repository URL if relevant, and provide specific details as to which raw blot/gel images, if any, are not available. Email us at plosone@plos.org if you have any questions.

Thank you for the suggestion. We have added the original uncropped and unadjusted images underlying all blot or gel results reported in a submission's figures or Supporting Information files. The file name is "S1_Raw_image".

6. If the reviewer comments include a recommendation to cite specific previously published works, please review and evaluate these publications to determine whether they are relevant and should be cited. There is no requirement to cite these works unless the editor has indicated otherwise.
[Note: HTML markup is below. Please do not edit.]

[NOTE: If reviewer comments were submitted as an attachment file, they will be attached to this email and accessible via the submission site. Please log into your account, locate the manuscript record, and check for the action link "View Attachments". If this link does not appear, there are no attachment files.]

Thank you for the suggestion. We agree to comply with those requirements.

To ensure your figures meet our technical requirements, please review our figure guidelines: https://journals.plos.org/plosone/s/figures

You may also use PLOS's free figure tool, NAAS, to help you prepare publication quality figures: https://journals.plos.org/plosone/s/figures#loc-tools-for-figure-preparation.

NAAS will assess whether your figures meet our technical requirements by comparing each figure against our figure specifications.

Thank you for the suggestion. We have revised all the figures in the manuscript and used NAAS to help preparation of publication quality figures as your recommendation

We highly value your recommendations and the publisher's guidelines. If any further requirements are needed to improve the publication, we would be pleased to provide them.

Sincerely,
Sompol Tapechum, M.D., Ph.D.
Assistant Professor
Department of Physiology
Faculty of Medicine Siriraj Hospital
Mahidol University, Bangkok, Thailand.
