# Supplementary material for: Inhibition of SUR1-TRPM4 attenuates astrocyte swelling and reactivity under oxygen-glucose deprivation/reoxygenation
Source: PLoS One. 2026 Jun 24;21(6):e0352151. doi: 10.1371/journal.pone.0352151 (PMC13293404; doi:10.1371/journal.pone.0352151)
Supplement: Attachment — Submitted filename: Response to Reviewers.docx [file pone.0352151.s005.docx]

Sompol Tapechum, M.D., Ph.D.

Assistant Professor

Department of Physiology

Faculty of Medicine Siriraj Hospital

Mahidol University, Bangkok, Thailand.

Tel. +662-419-9723

[Sompol.tap@mahidol.ac.th](mailto:Sompol.tap@mahidol.ac.th)

Alexander A. Mongin, Ph.D.

Academic Editor

PLOS One

May 21, 2026

Dear Editors,

We sincerely thank you and the reviewers for the careful evaluation of our manuscript and for the constructive comments and suggestions. We appreciate the opportunity to revise our work for further consideration in PLOS ONE.

Refer to the following response from the editor:

| **From:** | "PLOS ONE" plosone@plos.org |
| --- | --- |
| **Subject:** | PLOS ONE Decision: Revision required [PONE-D-26-12414R1] |

PONE-D-26-12414R1

Inhibition of SUR1–TRPM4 attenuates astrocyte swelling and reactivity under oxygen-glucose deprivation/reoxygenation

Dear Dr. Tapechum,

Thank you for submitting your manuscript to PLOS ONE. Your work has been evaluated by two experts in the field. After careful consideration, we feel that it has merit but does not fully meet PLOS ONE’s publication criteria as it currently stands. Therefore, we invite you to submit a revised version of the manuscript that addresses the points raised during the review process.

Author response:

We have carefully addressed all comments raised by the reviewers and the editor in a detailed point-by-point response document and have revised the manuscript accordingly. The responses for the comments are in red texts. In addition to addressing the reviewers’ and editor's feedback, we have corrected minor grammatical errors, typos, and wording inconsistencies throughout the manuscript.

I would like to draw your particular attention to the following points:

[1] Both reviewers expressed concerns about novelty. These concerns do not need to be addressed because PLOS ONE does not require novelty or major conceptual breakthroughs for publication, but instead focuses on methodological rigor and the soundness of the conclusions. However, all other issues raised by the reviewers should be specifically addressed in detailed point-by-point responses.

Author response:

We appreciate the clarification regarding novelty criteria at PLOS ONE. Despite the SUR1-TRPM4 is not a novel participant in astroglial injury, this study has further evaluated some particular molecular pathways involved in the development of the injury. These knowledges might contribute to further explanation of the full picture of the injury (Line 27-29, 93-94,632-634).

[2] Reviewer 2 raised a valid concern regarding the limitations of using an immortalized cell line in place of primary astrocytes. This limitation should be explicitly acknowledged in the manuscript text. I would also advise against referring to CTX-TNA2 cells as “astrocytes”; terms such as “CTX-TNA2 cells” or “astroglial cell line” would be more appropriate.

Author response:

Thank you very much for the comment. In response to Reviewer 2’s concern regarding the use of an immortalized cell line, we have explicitly acknowledged this limitation in the Discussion section (Line 436-437, 452-458). We also revised the terminology throughout the manuscript to avoid referring to CTX-TNA2 cells as “astrocytes,” and instead use terms such as “CTX-TNA2 cells” where appropriate.

[3] Reviewer 2 also cautioned against excessive generalization of the in vitro OGD findings to astrocyte pathology in situ. You may speculate about the potential significance and applicability of your findings, but statements such as (lines 459–461) “Our findings further indicate that SUR1–TRPM4 is involved in both astrocyte swelling and astrocyte reactivity following ischemic injury” should be avoided or substantially softened.

Author response:

Thank you for the comment, we agree that the in vitro OGD might not totally represent the in vivo condition and the conclusion should be carefully stated. So, we revised the statements that may overgeneralize the in vitro OGD findings to astrocyte pathology in situ. However, we believe that this in vitro model can represent some of the processes that occur in vivo. We also emphasized this limitation in the Discussion section (Line 459-465, 514-516, 537-538, 562, 595, 603-604, 617-620, 630-631).

[4] Please address the Editor’s concerns regarding statistical analyses. Most journals have now transitioned to reporting SD rather than SEM. In all cases where control data were normalized to 100% or unity (e.g., Fig. 1A, Fig. 3C, Fig. 4A,B, Fig. 6B–D), one-way ANOVA is not an appropriate statistical test. Instead, you may use one-sample t-tests with Bonferroni correction for multiple comparisons or consult a statistician regarding alternative approaches.

Author response:

Thank you very much for the comment. We addressed the concerns regarding statistical analyses by revising the statistical methods and reanalyzing the relevant datasets. SD is now reported instead of SEM throughout the manuscript. For datasets normalized to 100% or unity, we replaced one-way ANOVA with appropriate one-sample statistical analyses with Bonferroni correction, as recommended. The Methods section has also been updated accordingly (Line 2018-216). The figure legends from figure 1 to figure 8 have also been updated accordingly.

[5] Please clarify what is meant by the term “intracellular concentration” as presented in Fig. 2D.

Author response:

Thank you very much for the comment. We clarified the meaning of the term “intracellular concentration” from the Tomocube HT-2H system in the Method section (Line 158-160).

[6] It appears that in the Western blot analyses, β-actin loading controls were probed on separate membranes. If this is the case, it must be clearly stated in the Methods and/or figure legends.

Thank you very much for the comment. We clarify that the β-actin loading controls are from the same membrane as the proteins of interest. However, to increase the clarity of the pictures, the exposure time for the control β-actin bands is shorter than the proteins of interest due to low activity of proteins of interest as shown in the supplement data file. This ratio might not represent the real proteins level but should be possible for comparison among different treatments which are subjected to the same measure. This massage for clarification is added in the Methods and figure legends (Line 207-208).

[7] Both reviewers expressed concerns regarding the resolution and/or composition of the figures. Personally, I found the high-resolution versions generally acceptable (perhaps the reviewers did not access them). Nevertheless, I agree that several figures, particularly Figure 7, will be difficult to read when printed and may even be problematic in the online format. Please consider reorganizing these figures to improve overall legibility.

Thank you very much for the comment. We carefully reviewed and reorganized the all the figures to improve readability and presentation quality, particularly Figure 7. Higher-resolution and revised figure files have been uploaded with the resubmission.

Please submit your revised manuscript by Jun 24 2026 11:59PM. If you will need more time than this to complete your revisions, please reply to this message or contact the journal office at [plosone@plos.org](mailto:plosone@plos.org). When you're ready to submit your revision, log on to https://www.editorialmanager.com/pone/ and select the 'Submissions Needing Revision' folder to locate your manuscript file.

Please include the following items when submitting your revised manuscript:

- A letter that responds to each point raised by the academic editor and reviewer(s). You should upload this letter as a separate file labeled 'Response to Reviewers'.
- A marked-up copy of your manuscript that highlights changes made to the original version. You should upload this as a separate file labeled 'Revised Manuscript with Track Changes'.
- An unmarked version of your revised paper without tracked changes. You should upload this as a separate file labeled 'Manuscript'.

If you would like to make changes to your financial disclosure, please include your updated statement in your cover letter. Guidelines for resubmitting your figure files are available below the reviewer comments at the end of this letter.

If applicable, we recommend that you deposit your laboratory protocols in protocols.io to enhance the reproducibility of your results. Protocols.io assigns your protocol its own identifier (DOI) so that it can be cited independently in the future. For instructions see: <https://journals.plos.org/plosone/s/submission-guidelines#loc-laboratory-protocols>. Additionally, PLOS ONE offers an option for publishing peer-reviewed Lab Protocol articles, which describe protocols hosted on protocols.io. Read more information on sharing protocols at <https://plos.org/protocols?utm_medium=editorial-email&utm_source=authorletters&utm_campaign=protocols>.

As the corresponding author, your ORCID iD is verified in the submission system and will appear in the published article. PLOS supports the use of ORCID, and we encourage all coauthors to register for an ORCID iD and use it as well. Please encourage your coauthors to verify their ORCID iD within the submission system before final acceptance, as unverified ORCID iDs will not appear in the published article. *Only* the individual author can complete the verification step; PLOS staff *cannot* verify ORCID iDs on behalf of authors.

We look forward to receiving your revised manuscript.

Kind regards,

Alexander A. Mongin, Ph.D.
Academic Editor
PLOS One

Journal Requirements:

If the reviewer comments include a recommendation to cite specific previously published works, please review and evaluate these publications to determine whether they are relevant and should be cited. There is no requirement to cite these works unless the editor has indicated otherwise. 


[Note: HTML markup is below. Please do not edit.]

Reviewers' comments:

Reviewer's Responses to Questions

**Comments to the Author**

1. If the authors have adequately addressed your comments raised in a previous round of review and you feel that this manuscript is now acceptable for publication, you may indicate that here to bypass the “Comments to the Author” section, enter your conflict of interest statement in the “Confidential to Editor” section, and submit your "Accept" recommendation.

Reviewer #1: (No Response)

Reviewer #2: (No Response)

2. Is the manuscript technically sound, and do the data support the conclusions?

The manuscript must describe a technically sound piece of scientific research with data that supports the conclusions. Experiments must have been conducted rigorously, with appropriate controls, replication, and sample sizes. The conclusions must be drawn appropriately based on the data presented.

Reviewer #1: Yes

Reviewer #2: Partly

3. Has the statistical analysis been performed appropriately and rigorously?

Reviewer #1: Yes

Reviewer #2: (No Response)

4. Have the authors made all data underlying the findings in their manuscript fully available?

The [PLOS Data policy](http://www.plosone.org/static/policies.action#sharing) requires authors to make all data underlying the findings described in their manuscript fully available without restriction, with rare exception (please refer to the Data Availability Statement in the manuscript PDF file). The data should be provided as part of the manuscript or its supporting information, or deposited to a public repository. For example, in addition to summary statistics, the data points behind means, medians and variance measures should be available. If there are restrictions on publicly sharing data—e.g. participant privacy or use of data from a third party—those must be specified.

Reviewer #1: Yes

Reviewer #2: Yes

5. Is the manuscript presented in an intelligible fashion and written in standard English?

PLOS ONE does not copyedit accepted manuscripts, so the language in submitted articles must be clear, correct, and unambiguous. Any typographical or grammatical errors should be corrected at revision, so please note any specific errors here.

Reviewer #1: Yes

Reviewer #2: Yes

6. Review Comments to the Author

Please use the space provided to explain your answers to the questions above. You may also include additional comments for the author, including concerns about dual publication, research ethics, or publication ethics. (Please upload your review as an attachment if it exceeds 20,000 characters)

Reviewer #1: The authors employ an astrocytic cell line and an in vitro model of ischemia–reperfusion, combined with pharmacological interventions, to demonstrate that glibenclamide-sensitive SUR1-TRPM4 channels are not only involved in astrocytic swelling but also contribute to ischemia–reperfusion-induced ROS production, ER stress, and inflammatory responses.
Overall, the manuscript is clearly written and well organized. The experimental design is described in sufficient detail, and the results are presented and discussed in a comprehensive manner. The data appear robust and convincing, and the experiments were conducted rigorously, with appropriate replication across treatment conditions.

Major Comments
Figure quality
The quality of the figures is currently insufficient for publication. Several images are of low resolution, and some labels are difficult to read. All figures should be replaced with high-resolution versions to ensure clarity and readability.

Author response:

We thank the reviewer for this comment. All figures have been replaced with higher-resolution versions to improve clarity and readability. In particular, Figure 7 has been reorganized and enlarged to improve visualization of fluorescence signals and labeling. We also adjusted font sizes and panel spacing throughout the figures to enhance legibility in both online and print formats.

Redundancy of astrocyte swelling experiments
The role of glibenclamide in reducing astrocyte swelling via SUR1-TRPM4 channels has already been well established in the literature. Repeating these findings may not add substantial novelty. The authors may consider reducing this section and instead placing greater emphasis on the less well-characterized downstream effects.

Author response:

Thank you very much, we appreciate this important suggestion. The role of SUR1-TRPM4 in astrocyte swelling has been published elsewhere. However, our study focused more extensively on the downstream consequences associated with SUR1–TRPM4 activation, including ROS production, ER stress, inflammatory signaling, and conditioned medium effects. We also revised the Discussion to better clarify the rationale and contribution of the present study within the existing literature (Line 450-452,463-466, 606-617). Additional citations are added as appropriate.

Lack of mechanistic insight into downstream signaling
While the study convincingly demonstrates downstream effects such as ROS production, ER stress, and inflammation, the underlying signaling mechanisms remain unclear and are not addressed in the manuscript. For example, it would be important to consider whether these effects are mediated by changes in intracellular calcium levels. Additionally, the factors responsible for the effects of the conditioned media are not identified. Including experiments to explore these mechanisms would significantly strengthen the manuscript and provide valuable mechanistic insight.

Author response:

We thank the reviewer for this thoughtful comment. We agree that intracellular calcium signaling and identification of factors within conditioned media represent important mechanistic questions. However, these investigations were beyond the scope of the current study. We have now explicitly acknowledged these limitations in the Discussion section and added a statement indicating that future studies are needed to investigate calcium-dependent mechanisms and to identify specific mediators present in conditioned media responsible for downstream effects (Line 478-485, 597-602).

Minor Comments
Therapeutic context of glibenclamide
In the final part of the Introduction, the authors suggest that glibenclamide may have therapeutic potential. However, this concept has already been proposed in earlier studies, and the compound has been under preclinical investigation for brain ischemia for some time. The text should be revised to better reflect the current state of the field.

Author response:

We thank the reviewer for this comment. We agree with the reviewer and revised the final section of the Introduction to better reflect the current state of the field (Line 89-94). The revised text now acknowledges prior preclinical investigations of glibenclamide in ischemic brain injury and avoids overstating novelty or therapeutic implications. In the manuscript, where appropriate, we emphasized on other aspect of study beside the already know role of glibenclamide in reduce astrocyte swelling (Line 630-632).

Figure 8 – pSTAT3 interpretation
Figure 8 does not fully reflect the described results. Specifically, pSTAT3 levels appear to be increased not only in cells treated with conditioned media but also - more prominently - in ischemic astrocytes themselves. This aspect should be clearly represented in the figure and/or clarified in the figure legend.

Author response:

We thank the reviewer for this observation. We have revised the description of Figure 8 and clarified in both the Results section (Line 416-425) and figure legend (Line 427-434) that pSTAT3 levels were elevated not only in conditioned medium-treated cells but also in ischemic CTX-TNA2 cells themselves (Figure 6). The revised figure presentation more accurately reflects these findings. These findings are also discussed in the Discussion section (Line 578-582).

Reviewer #2:

1. This m/s is not particularly novel, as earlier studies have shown similar results. As the authors know, Simard’s group has published work identical to the current findings (e.g., SUR1-TRPM4 channel activation and phasic secretion of MMP-9 induced by tPA in brain endothelial cells, Volodymyr Gerzanich,….J. Marc Simard; Cell swelling and a nonselective cation channel regulated by internal Ca2+ and ATP in native reactive astrocytes from adult rat brain. JNeurosci 21: 6512–6521), and the authors cited some of them, except for the pharmacological manipulation.

Author response:

We appreciate the reviewer’s comments, we revised the manuscript to better position our findings within the existing literature and clarified the aspects of downstream cellular responses examined in the present study. We also reviewed the suggested references and incorporated relevant citations where appropriate (Line 27-29, 93-94,632-634).

2. The cell line used in this study is unacceptable. The rat astrocyte cell line CTX-TNA2 (ATCC® CRL-2006™) is an immortalized, type 1 astrocyte line derived from the cortex of 1-day-old Sprague-Dawley rats. These adherent cells, often used, exhibit a fibroblast-like morphology. More importantly, only 20% of these cells exhibit glial fibrillary acidic protein (GFAP) immunoreactivity. Thus, this cell line can't be used for any GFAP-related applications. As the authors know, these cells often lack specific functions of normal cells, such as metabolic pathways or tissue-specific markers, due to changes in gene expression and metabolic rearrangement. Since these cells are derived from tumors, the results should have been validated in primary cells to ensure physiological relevance.

Author response:

We thank the reviewer for this important concern. We acknowledge that CTX-TNA2 is an immortalized astroglial cell line with known limitations compared with primary astrocytes. Accordingly, we have explicitly discussed these limitations in the revised Discussion section(Line 453-459). In addition, throughout the manuscript, we replaced the term “astrocytes” with “CTX-TNA2 cells” where appropriate to avoid overinterpretation of the cellular phenotype. We agree that validation in primary astrocytes would further strengthen physiological relevance and have acknowledged this as an important direction for future studies (Line 635-637).

3. The argument that inhibition of SUR1–TRPM4 attenuates astrocyte swelling in injury-like settings, and the strongest evidence is in activated astrocytes and post-ischemic brain tissue rather than in a pure OGD-only astrocyte culture model, is hard to accept. Because the in vitro system lacks blood components. This needs to be reversed.

Author response:

We appreciate the reviewer’s concern regarding extrapolation of in vitro OGD findings to in vivo conditions. We have substantially softened statements that generalized our findings to astrocyte pathology in situ and clarified that the current conclusions are limited to the in vitro OGD/reoxygenation model employed in this study. We also acknowledged that the absence of blood-derived factors and multicellular interactions represents an important limitation of the model system (Line 460-466).

4. The manuscript is poorly written and lacks methodological details. For example, the authors noted that they used 96-well plates. If that’s the case, how many plates were used per condition? TomoDish imaging dishes, what does n=4 mean?

Author response:

We thank the reviewer for highlighting this issue. Additional methodological details have now been added throughout the Methods section (Line 128-137, 141-142, 164-165), including the number of wells, plates, biological replicates, and clarification of sample sizes (n values) for each experiment. We also clarified the meaning of “n=4” for imaging experiments performed using TomoDish imaging dishes (Line 266-267).

5. The major issues when using Astrocyte-conditioned medium (ACM) with naive cells are reproducibility and a lack of standardization in its secretome composition. Because ACM is produced by cultured astrocytes, which often default to a reactive or immature phenotype in 2/3D systems, the precise cocktail of secreted proteins, lipids, and cytokines can vary significantly depending on astrocyte culture conditions, passage number, and basal media. It's unclear how the authors address these issues.

Author response:

We appreciate this important point. We have now added additional methodological details regarding ACM preparation, including cell passage conditions, culture conditions, timing of collection, and normalization procedures used to improve reproducibility. We also acknowledged in the Discussion that variability in conditioned media composition is an inherent limitation of this experimental approach(Line 578-582, 597-602).

6. The authors used a microplate reader for ROS measurement. But flow cytometry is a reliable tool for measuring ROS using the Invitrogen Total Reactive Oxygen Species (ROS) Assay Kit (Cat. No. 88-5930-74). Because the fluorescent signal will now be retained after cell permeabilization.

Author response:

We thank the reviewer for this valuable suggestion. We agree that flow cytometry is a sensitive and widely used method for ROS analysis. In the present study, ROS levels were measured using a microplate reader-based assay to enable standardized comparisons among experimental groups under identical experimental conditions. This method is also suggested by the manufacturer of the product in addition to flow cytometry. We have clarified this point in the Methods section (Line 167–168).

7. Provide the original number for Fig. 1A instead of expressing it as a percentage.

Author response:

We appreciate the reviewer’s helpful suggestion. The original values corresponding to the normalized data shown in Figure 1A have now been provided in the supplementary data files. For clarity of presentation, the data in the figure are expressed as percentages. Statistical analysis was performed using the method recommended by the editor(Line 210-217, 233-240).

8. It is difficult to compare legends with figures because the legends are far from the figures. This may be due to the journal's style (the journal should look into it).

Author response:

We understand the reviewer’s concern. The separation of figure legends from figures follows journal formatting requirements; however, we revised figure organization and labeling to improve readability and facilitate comparison between figures and legends.

9. The fluorescence images are of poor quality (Fig 7 specifically). The astrocytes may have been stained with May-Grunwald to better assess morphology or density. Because we don’t know what cells we are looking at.

Author response:

We thank the reviewer for this comment. Higher-resolution fluorescence images have now been provided, particularly for Figure 7. We also reorganized the figure layout to improve visualization. In addition, we clarified the staining methods used for cell identification and morphology assessment within the figure legends and Methods section (Line 236).

10. It's unclear what concentrations of Gli were used (it should be explicitly mentioned in all figure legends).

Author response:

We appreciate the reviewer’s observation. The concentrations of glibenclamide used in each experiment are now explicitly stated in all relevant figure legends and clarified in the Methods section (Line 166).

11. Fig. 9 needs to be redrawn. Astrocyte death and reactivity can occur under the same conditions, particularly in severe neurodegenerative diseases, stroke, and traumatic brain injuries. While reactive astrogliosis is traditionally viewed as a protective, hypertrophy-driven (growth) response to damage, it often transitions into a pathological state in which reactive astrocytes lose their supportive functions, become neurotoxic, and eventually die. This raises the question: Did the authors measure the neurotoxic substances associated with this condition? If so, what are they? Since this is an in vitro condition, what may have occurred in vivo?

Author response:

We thank the reviewer for this insightful comment. We agree that astrocyte reactivity and astroglial cell death may coexist under pathological conditions. Figure 9 has been revised to better reflect the complexity and limitations of the proposed model (Line 604-627). We also clarified in the Discussion that the current in vitro findings do not fully recapitulate in vivo conditions and, that, additional studies would be necessary to identify neurotoxic mediators and evaluate their relevance in vivo (Line 460-466).

7. PLOS authors have the option to publish the peer review history of their article ([what does this mean?](https://journals.plos.org/plosone/s/editorial-and-peer-review-process#loc-peer-review-history)). If published, this will include your full peer review and any attached files.

If you choose “no”, your identity will remain anonymous but your review may still be made public.

**Do you want your identity to be public for this peer review?** For information about this choice, including consent withdrawal, please see our [Privacy Policy](https://www.plos.org/privacy-policy).

 Reviewer #1: **Yes:** Helena Pivonkova

Reviewer #2: **Yes:** Arumugam R. Jayakumar

[NOTE: If reviewer comments were submitted as an attachment file, they will be attached to this email and accessible via the submission site. Please log into your account, locate the manuscript record, and check for the action link "View Attachments". If this link does not appear, there are no attachment files.]

To ensure your figures meet our technical requirements, please review our figure guidelines: https://journals.plos.org/plosone/s/figures

You may also use PLOS’s free figure tool, NAAS, to help you prepare publication quality figures: https://journals.plos.org/plosone/s/figures#loc-tools-for-figure-preparation.

NAAS will assess whether your figures meet our technical requirements by comparing each figure against our figure specifications.

*In compliance with data protection regulations, you may request that we remove your personal registration details at any time.* [*(Remove my information/details)*](https://www.editorialmanager.com/pone/login.asp?a=r)*. Please contact the publication office if you have any questions.*

Once again, we sincerely thank the Editor and Reviewers for their valuable comments and suggestions, which substantially improved the quality and clarity of our manuscript.

Sincerely,

Sompol Tapechum, M.D., Ph.D.

Assistant Professor

Department of Physiology

Faculty of Medicine Siriraj Hospital

Mahidol University, Bangkok, Thailand.
