# Supplementary material for: Inhibition of SUR1-TRPM4 attenuates astrocyte swelling and reactivity under oxygen-glucose deprivation/reoxygenation
Source: PLoS One. 2026 Jun 24;21(6):e0352151. doi: 10.1371/journal.pone.0352151 (PMC13293404; doi:10.1371/journal.pone.0352151)
Supplement: Peer review history

# Editor report: Decision Letter 0

**DOI:** 10.1371/journal.pone.0352151.r001

**Authors:** Alexander Mongin

17 Mar 2026

PONE-D-26-12414

Inhibition of SUR1–TRPM4 attenuates astrocyte swelling and reactivity under oxygen-glucose deprivation/reoxygenation

PLOS One

Dear Dr. Tapechum,

Thank you for submitting your manuscript to PLOS ONE. During preliminary evaluation of your manuscript, I have found that it has merit but does not fully meet PLOS ONE’s publication criteria as it currently stands. Therefore, we invite you to submit a revised version of the manuscript that addresses the points raised during the editorial screening process.

Specifically:

During the preliminary evaluation of your manuscript, I noted that the submission does not comply with the PLOS ONE Policy on Data Availability. As stated in the journal’s guidelines:

“PLOS journals require authors to make all data necessary to replicate their study’s findings publicly available without restriction at the time of publication.”

Compliance can be achieved by either:

(a) Providing all critical primary data and unedited images (e.g., full-length western blots, uncropped ICC and EM images) as supplemental files; **or**

(b) Depositing all critical primary data and unedited primary images in an appropriate public repository.

**Please note that any embargoed data must still be made available for inspection during the peer review process.**  Failure to meet this requirement will result in rejection of the manuscript without further review.

In addition, I strongly recommend including individual data points (scatter plots) overlaid on all bar graphs to improve transparency and enhance the rigor of data presentation.

Please submit your revised manuscript by May 01 2026 11:59PM. If you will need more time than this to complete your revisions, please reply to this message or contact the journal office at plosone@plos.org. When you're ready to submit your revision, log on to https://www.editorialmanager.com/pone/ and select the 'Submissions Needing Revision' folder to locate your manuscript file.

Please include the following items when submitting your revised manuscript:

- A letter that responds to each point raised by the academic editor and reviewer(s). You should upload this letter as a separate file labeled 'Response to Reviewers'.
- A marked-up copy of your manuscript that highlights changes made to the original version. You should upload this as a separate file labeled 'Revised Manuscript with Track Changes'.
- An unmarked version of your revised paper without tracked changes. You should upload this as a separate file labeled 'Manuscript'.

If you would like to make changes to your financial disclosure, please include your updated statement in your cover letter. Guidelines for resubmitting your figure files are available below the reviewer comments at the end of this letter.

If applicable, we recommend that you deposit your laboratory protocols in protocols.io to enhance the reproducibility of your results. Protocols.io assigns your protocol its own identifier (DOI) so that it can be cited independently in the future. For instructions see: https://journals.plos.org/plosone/s/submission-guidelines#loc-laboratory-protocols. Additionally, PLOS ONE offers an option for publishing peer-reviewed Lab Protocol articles, which describe protocols hosted on protocols.io. Read more information on sharing protocols at https://plos.org/protocols?utm_medium=editorial-email&utm_source=authorletters&utm_campaign=protocols.

We look forward to receiving your revised manuscript.

Kind regards,

Alexander A. Mongin, Ph.D.

Academic Editor

PLOS One

Journal Requirements:

When submitting your revision, we need you to address these additional requirements.

1. Please ensure that your manuscript meets PLOS ONE's style requirements, including those for file naming. The PLOS ONE style templates can be found at

https://journals.plos.org/plosone/s/file?id=wjVg/PLOSOne_formatting_sample_main_body.pdf and

https://journals.plos.org/plosone/s/file?id=ba62/PLOSOne_formatting_sample_title_authors_affiliations.pdf

2. Thank you for stating in your Funding Statement:

“This research was supported by Siriraj Research and Development Fund from the Faculty of Siriraj Hospital, Mahidol University, Thailand, to ST under the grant NO. R016732027.  The funder did not play any role in the study design, data collection and analysis, decision to publish, or preparation of the manuscript.

URL: https://si.mahidol.ac.th/th/research-academics/research/fund_research.html”

Please provide an amended statement that declares *all* the funding or sources of support (whether external or internal to your organization) received during this study, as detailed online in our guide for authors at http://journals.plos.org/plosone/s/submit-now. Please also include the statement “There was no additional external funding received for this study.” in your updated Funding Statement.

Please include your amended Funding Statement within your cover letter. We will change the online submission form on your behalf.

3. Thank you for stating the following financial disclosure:

“This research was supported by Siriraj Research and Development Fund from the Faculty of Siriraj Hospital, Mahidol University, Thailand, to ST under the grant NO. R016732027.  The funder did not play any role in the study design, data collection and analysis, decision to publish, or preparation of the manuscript.

URL: https://si.mahidol.ac.th/th/research-academics/research/fund_research.html”

We note that one or more of the authors is affiliated with the funding organization, indicating the funder may have had some role in the design, data collection, analysis or preparation of your manuscript for publication; in other words, the funder played an indirect role through the participation of the co-authors. If the funding organization did not play a role in the study design, data collection and analysis, decision to publish, or preparation of the manuscript and only provided financial support in the form of authors' salaries and/or research materials, please do the following:

a. Review your statements relating to the author contributions, and ensure you have specifically and accurately indicated the role(s) that these authors had in your study. These amendments should be made in the online form.

b. Confirm in your cover letter that you agree with the following statement, and we will change the online submission form on your behalf:

“The funder provided support in the form of salaries for authors [insert relevant initials], but did not have any additional role in the study design, data collection and analysis, decision to publish, or preparation of the manuscript. The specific roles of these authors are articulated in the ‘author contributions’ section.

>4. We note that your Data Availability Statement is currently as follows: All relevant data are within the manuscript and its Supporting Information files.

Please confirm at this time whether or not your submission contains all raw data required to replicate the results of your study. Authors must share the “minimal data set” for their submission. PLOS defines the minimal data set to consist of the data required to replicate all study findings reported in the article, as well as related metadata and methods (https://journals.plos.org/plosone/s/data-availability#loc-minimal-data-set-definition).

For example, authors should submit the following data:

- The values behind the means, standard deviations and other measures reported;

- The values used to build graphs;

- The points extracted from images for analysis.

Authors do not need to submit their entire data set if only a portion of the data was used in the reported study.

If your submission does not contain these data, please either upload them as Supporting Information files or deposit them to a stable, public repository and provide us with the relevant URLs, DOIs, or accession numbers. For a list of recommended repositories, please see https://journals.plos.org/plosone/s/recommended-repositories.

If there are ethical or legal restrictions on sharing a de-identified data set, please explain them in detail (e.g., data contain potentially sensitive information, data are owned by a third-party organization, etc.) and who has imposed them (e.g., an ethics committee). Please also provide contact information for a data access committee, ethics committee, or other institutional body to which data requests may be sent. If data are owned by a third party, please indicate how others may request data access.

5. PLOS ONE now requires that authors provide the original uncropped and unadjusted images underlying all blot or gel results reported in a submission’s figures or Supporting Information files. This policy and the journal’s other requirements for blot/gel reporting and figure preparation are described in detail at https://journals.plos.org/plosone/s/figures#loc-blot-and-gel-reporting-requirements and https://journals.plos.org/plosone/s/figures#loc-preparing-figures-from-image-files. When you submit your revised manuscript, please ensure that your figures adhere fully to these guidelines and provide the original underlying images for all blot or gel data reported in your submission. See the following link for instructions on providing the original image data: https://journals.plos.org/plosone/s/figures#loc-original-images-for-blots-and-gels.

In your cover letter, please note whether your blot/gel image data are in Supporting Information or posted at a public data repository, provide the repository URL if relevant, and provide specific details as to which raw blot/gel images, if any, are not available. Email us at plosone@plos.org if you have any questions.

6. If the reviewer comments include a recommendation to cite specific previously published works, please review and evaluate these publications to determine whether they are relevant and should be cited. There is no requirement to cite these works unless the editor has indicated otherwise.

[Note: HTML markup is below. Please do not edit.]

[NOTE: If reviewer comments were submitted as an attachment file, they will be attached to this email and accessible via the submission site. Please log into your account, locate the manuscript record, and check for the action link "View Attachments". If this link does not appear, there are no attachment files.]

To ensure your figures meet our technical requirements, please review our figure guidelines: https://journals.plos.org/plosone/s/figures

You may also use PLOS’s free figure tool, NAAS, to help you prepare publication quality figures: https://journals.plos.org/plosone/s/figures#loc-tools-for-figure-preparation.

NAAS will assess whether your figures meet our technical requirements by comparing each figure against our figure specifications.

# Author comment: Author response to Decision Letter 1

**DOI:** 10.1371/journal.pone.0352151.r002

30 Mar 2026

Sompol Tapechum, M.D., Ph.D.

Assistant Professor

Department of Physiology

Faculty of Medicine Siriraj Hospital

Mahidol University, Bangkok, Thailand.

Tel. +662-419-9723

Sompol.tap@mahidol.ac.th

Alexander A. Mongin, Ph.D.

Academic Editor

PLOS One

March 30, 2026

Dear Editors,

Thank you very much for your valuable comments and constructive suggestions. we consider all of them to be highly important and helpful in improving the quality of our manuscript. Below, we provide a detailed, point-by-point response, along with the corresponding revisions made in accordance with the reviewers’ comments and recommendations. The responses for the comments are in red texts.

PONE-D-26-12414

Inhibition of SUR1–TRPM4 attenuates astrocyte swelling and reactivity under oxygen-glucose deprivation/reoxygenation

PLOS One

Dear Dr. Tapechum,

Thank you for submitting your manuscript to PLOS ONE. During preliminary evaluation of your manuscript, I have found that it has merit but does not fully meet PLOS ONE’s publication criteria as it currently stands. Therefore, we invite you to submit a revised version of the manuscript that addresses the points raised during the editorial screening process.

Specifically:

During the preliminary evaluation of your manuscript, I noted that the submission does not comply with the PLOS ONE Policy on Data Availability. As stated in the journal’s guidelines:

“PLOS journals require authors to make all data necessary to replicate their study’s findings publicly available without restriction at the time of publication.”

Compliance can be achieved by either:

(a) Providing all critical primary data and unedited images (e.g., full-length western blots, uncropped ICC and EM images) as supplemental files; or

(b) Depositing all critical primary data and unedited primary images in an appropriate public repository.

Please note that any embargoed data must still be made available for inspection during the peer review process. Failure to meet this requirement will result in rejection of the manuscript without further review.

Thank you for the suggestion, we consider the PLOS ONE Policy on Data Availability is very important. We added two Supporting Information files with this revised manuscript.

1. “S1_Raw_data” is the excel file containing the raw data of the results in this manuscript.

2. “S1_Raw_image” contains the original western blot and immunofluorescent images used in this manuscript.

In addition, I strongly recommend including individual data points (scatter plots) overlaid on all bar graphs to improve transparency and enhance the rigor of data presentation.

Thank you for the suggestion, we added individual data points (scatter plots) overlaid on all bar graphs to improve transparency and enhance the rigor of data presentation.

Please submit your revised manuscript by May 01 2026 11:59PM. If you will need more time than this to complete your revisions, please reply to this message or contact the journal office at plosone@plos.org. When you're ready to submit your revision, log on to https://www.editorialmanager.com/pone/ and select the 'Submissions Needing Revision' folder to locate your manuscript file.

Please include the following items when submitting your revised manuscript:

A letter that responds to each point raised by the academic editor and reviewer(s). You should upload this letter as a separate file labeled 'Response to Reviewers'.

A marked-up copy of your manuscript that highlights changes made to the original version. You should upload this as a separate file labeled 'Revised Manuscript with Track Changes'.

An unmarked version of your revised paper without tracked changes. You should upload this as a separate file labeled 'Manuscript'.

Thank you for the suggestion, we provided this 'Response to Reviewers' as you recommended.

We also included a marked-up copy of the manuscript that highlights changes made to the original version in a file name 'Revised Manuscript with Track Changes' and an unmarked version of the revised manuscript without tracked changes in a file name 'Manuscript'.

If you would like to make changes to your financial disclosure, please include your updated statement in your cover letter. Guidelines for resubmitting your figure files are available below the reviewer comments at the end of this letter.

Thank you for the suggestion. We included the updated statement in the cover letter.

If applicable, we recommend that you deposit your laboratory protocols in protocols.io to enhance the reproducibility of your results. Protocols.io assigns your protocol its own identifier (DOI) so that it can be cited independently in the future. For instructions see: https://journals.plos.org/plosone/s/submission-guidelines#loc-laboratory-protocols. Additionally, PLOS ONE offers an option for publishing peer-reviewed Lab Protocol articles, which describe protocols hosted on protocols.io. Read more information on sharing protocols at https://plos.org/protocols?utm_medium=editorial-email&utm_source=authorletters&utm_campaign=protocols.

Thank you for the suggestion. We decided to add two supporting files containing all raw data in comply with PLOS ONE Policy on Data Availability. The two supporting files are those mentioned above, “S1_Raw_data” and “S1_Raw_image”.

We look forward to receiving your revised manuscript.

Kind regards,

Alexander A. Mongin, Ph.D.

Academic Editor

PLOS One

Journal Requirements:

When submitting your revision, we need you to address these additional requirements.

1. Please ensure that your manuscript meets PLOS ONE's style requirements, including those for file naming. The PLOS ONE style templates can be found at

https://journals.plos.org/plosone/s/file?id=wjVg/PLOSOne_formatting_sample_main_body.pdf and

https://journals.plos.org/plosone/s/file?id=ba62/PLOSOne_formatting_sample_title_authors_affiliations.pdf

Thank you for the suggestion. We have revised our manuscript to meet PLOS ONE's style requirements including the file naming. We also rearranged the manuscript writing and correction of typographic errors for the clarity of the manuscript while still maintained the integrity of our work.

2. Thank you for stating in your Funding Statement:

“This research was supported by Siriraj Research and Development Fund from the Faculty of Siriraj Hospital, Mahidol University, Thailand, to ST under the grant NO. R016732027. The funder did not play any role in the study design, data collection and analysis, decision to publish, or preparation of the manuscript.

URL: https://si.mahidol.ac.th/th/research-academics/research/fund_research.html”

Please provide an amended statement that declares *all* the funding or sources of support (whether external or internal to your organization) received during this study, as detailed online in our guide for authors at http://journals.plos.org/plosone/s/submit-now. Please also include the statement “There was no additional external funding received for this study.” in your updated Funding Statement.

Please include your amended Funding Statement within your cover letter. We will change the online submission form on your behalf.

Thank you for the suggestion. We have updated the funding statement by adding “There was no additional external funding received for this study.” online and also declared in the cover letter.

3. Thank you for stating the following financial disclosure:

“This research was supported by Siriraj Research and Development Fund from the Faculty of Siriraj Hospital, Mahidol University, Thailand, to ST under the grant NO. R016732027. The funder did not play any role in the study design, data collection and analysis, decision to publish, or preparation of the manuscript.

URL: https://si.mahidol.ac.th/th/research-academics/research/fund_research.html”

We note that one or more of the authors is affiliated with the funding organization, indicating the funder may have had some role in the design, data collection, analysis or preparation of your manuscript for publication; in other words, the funder played an indirect role through the participation of the co-authors. If the funding organization did not play a role in the study design, data collection and analysis, decision to publish, or preparation of the manuscript and only provided financial support in the form of authors' salaries and/or research materials, please do the following:

a. Review your statements relating to the author contributions, and ensure you have specifically and accurately indicated the role(s) that these authors had in your study. These amendments should be made in the online form.

Thank you for the suggestion. We have reviewed our statements regarding the author contributions and affirm that all the information provided is accurate.

b. Confirm in your cover letter that you agree with the following statement, and we will change the online submission form on your behalf:

“The funder provided support in the form of salaries for authors [insert relevant initials], but did not have any additional role in the study design, data collection and analysis, decision to publish, or preparation of the manuscript. The specific roles of these authors are articulated in the ‘author contributions’ section.

Thank you for the suggestion. We have confirmed in the cover letter that we agreed with the following statement.

“The funder provided support in the form of salaries for authors [NP, RS, UV, ST], but did not have any additional role in the study design, data collection and analysis, decision to publish, or preparation of the manuscript. The specific roles of these authors are articulated in the ‘author contributions’ section.”

4. We note that your Data Availability Statement is currently as follows: All relevant data are within the manuscript and its Supporting Information files.

Please confirm at this time whether or not your submission contains all raw data required to replicate the results of your study. Authors must share the “minimal data set” for their submission. PLOS defines the minimal data set to consist of the data required to replicate all study findings reported in the article, as well as related metadata and methods (https://journals.plos.org/plosone/s/data-availability#loc-minimal-data-set-definition).

For example, authors should submit the following data:

- The values behind the means, standard deviations and other measures reported;

- The values used to build graphs;

- The points extracted from images for analysis.

Authors do not need to submit their entire data set if only a portion of the data was used in the reported study.

If your submission does not contain these data, please either upload them as Supporting Information files or deposit them to a stable, public repository and provide us with the relevant URLs, DOIs, or accession numbers. For a list of recommended repositories, please see https://journals.plos.org/plosone/s/recommended-repositories.

If there are ethical or legal restrictions on sharing a de-identified data set, please explain them in detail (e.g., data contain potentially sensitive information, data are owned by a third-party organization, etc.) and who has imposed them (e.g., an ethics committee). Please also provide contact information for a data access committee, ethics committee, or other institutional body to which data requests may be sent. If data are owned by a third party, please indicate how others may request data access.

Thank you for the suggestion. We have added all relevant data in the manuscript and the two Supporting Information files to comply with the above statement.

5. PLOS ONE now requires that authors provide the original uncropped and unadjusted images underlying all blot or gel results reported in a submission’s figures or Supporting Information files. This policy and the journal’s other requirements for blot/gel reporting and figure preparation are described in detail at https://journals.plos.org/plosone/s/figures#loc-blot-and-gel-reporting-requirements and https://journals.plos.org/plosone/s/figures#loc-preparing-figures-from-image-files. When you submit your revised manuscript, please ensure that your figures adhere fully to these guidelines and provide the original underlying images for all blot or gel data reported in your submission. See the following link for instructions on providing the original image data: https://journals.plos.org/plosone/s/figures#loc-original-images-for-blots-and-gels.

In your cover letter, please note whether your blot/gel image data are in Supporting Information or posted at a public data repository, provide the repository URL if relevant, and provide specific details as to which raw blot/gel images, if any, are not available. Email us at plosone@plos.org if you have any questions.

Thank you for the suggestion. We have added the original uncropped and unadjusted images underlying all blot or gel results reported in a submission’s figures or Supporting Information files. The file name is “S1_Raw_image”.

6. If the reviewer comments include a recommendation to cite specific previously published works, please review and evaluate these publications to determine whether they are relevant and should be cited. There is no requirement to cite these works unless the editor has indicated otherwise.

[Note: HTML markup is below. Please do not edit.]

[NOTE: If reviewer comments were submitted as an attachment file, they will be attached to this email and accessible via the submission site. Please log into your account, locate the manuscript record, and check for the action link "View Attachments". If this link does not appear, there are no attachment files.]

Thank you for the suggestion. We agree to comply with those requirements.

To ensure your figures meet our technical requirements, please review our figure guidelines: https://journals.plos.org/plosone/s/figures

You may also use PLOS’s free figure tool, NAAS, to help you prepare publication quality figures: https://journals.plos.org/plosone/s/figures#loc-tools-for-figure-preparation.

NAAS will assess whether your figures meet our technical requirements by comparing each figure against our figure specifications.

Thank you for the suggestion. We have revised all the figures in the manuscript and used NAAS to help preparation of publication quality figures as your recommendation

We highly value your recommendations and the publisher’s guidelines. If any further requirements are needed to improve the publication, we would be pleased to provide them.

Sincerely,

Sompol Tapechum, M.D., Ph.D.

Assistant Professor

Department of Physiology

Faculty of Medicine Siriraj Hospital

Mahidol University, Bangkok, Thailand.

Attachment Submitted filename: Response to Reviewers.pdf

# Peer review: Decision Letter 1

**DOI:** 10.1371/journal.pone.0352151.r003

**Authors:** Alexander Mongin, Alexander Mongin

10 May 2026

PONE-D-26-12414R1

Inhibition of SUR1–TRPM4 attenuates astrocyte swelling and reactivity under oxygen-glucose deprivation/reoxygenation

Dear Dr. Tapechum,

Thank you for submitting your manuscript to PLOS ONE. Your work has been evaluated by two experts in the field. After careful consideration, we feel that it has merit but does not fully meet PLOS ONE’s publication criteria as it currently stands. Therefore, we invite you to submit a revised version of the manuscript that addresses the points raised during the review process.

I would like to draw your particular attention to the following points:

[1] Both reviewers expressed concerns about novelty. These concerns do not need to be addressed because PLOS ONE does not require novelty or major conceptual breakthroughs for publication, but instead focuses on methodological rigor and the soundness of the conclusions. However, all other issues raised by the reviewers should be specifically addressed in detailed point-by-point responses.

[2] Reviewer 2 raised a valid concern regarding the limitations of using an immortalized cell line in place of primary astrocytes. This limitation should be explicitly acknowledged in the manuscript text. I would also advise against referring to CTX-TNA2 cells as “astrocytes”; terms such as “CTX-TNA2 cells” or “astroglial cell line” would be more appropriate.

[3] Reviewer 2 also cautioned against excessive generalization of the in vitro OGD findings to astrocyte pathology in situ. You may speculate about the potential significance and applicability of your findings, but statements such as (lines 459–461) “Our findings further indicate that SUR1–TRPM4 is involved in both astrocyte swelling and astrocyte reactivity following ischemic injury” should be avoided or substantially softened.

[4] Please address the Editor’s concerns regarding statistical analyses. Most journals have now transitioned to reporting SD rather than SEM. In all cases where control data were normalized to 100% or unity (e.g., Fig. 1A, Fig. 3C, Fig. 4A,B, Fig. 6B–D), one-way ANOVA is not an appropriate statistical test. Instead, you may use one-sample t-tests with Bonferroni correction for multiple comparisons or consult a statistician regarding alternative approaches.

[5] Please clarify what is meant by the term “intracellular concentration” as presented in Fig. 2D.

[6] It appears that in the Western blot analyses, β-actin loading controls were probed on separate membranes. If this is the case, it must be clearly stated in the Methods and/or figure legends.

[7] Both reviewers expressed concerns regarding the resolution and/or composition of the figures. Personally, I found the high-resolution versions generally acceptable (perhaps the reviewers did not access them). Nevertheless, I agree that several figures, particularly Figure 7, will be difficult to read when printed and may even be problematic in the online format. Please consider reorganizing these figures to improve overall legibility.

Please submit your revised manuscript by Jun 24 2026 11:59PM. If you will need more time than this to complete your revisions, please reply to this message or contact the journal office at plosone@plos.org. When you're ready to submit your revision, log on to https://www.editorialmanager.com/pone/ and select the 'Submissions Needing Revision' folder to locate your manuscript file.

Please include the following items when submitting your revised manuscript:

- A letter that responds to each point raised by the academic editor and reviewer(s). You should upload this letter as a separate file labeled 'Response to Reviewers'.
- A marked-up copy of your manuscript that highlights changes made to the original version. You should upload this as a separate file labeled 'Revised Manuscript with Track Changes'.
- An unmarked version of your revised paper without tracked changes. You should upload this as a separate file labeled 'Manuscript'.

If you would like to make changes to your financial disclosure, please include your updated statement in your cover letter. Guidelines for resubmitting your figure files are available below the reviewer comments at the end of this letter.

If applicable, we recommend that you deposit your laboratory protocols in protocols.io to enhance the reproducibility of your results. Protocols.io assigns your protocol its own identifier (DOI) so that it can be cited independently in the future. For instructions see: https://journals.plos.org/plosone/s/submission-guidelines#loc-laboratory-protocols. Additionally, PLOS ONE offers an option for publishing peer-reviewed Lab Protocol articles, which describe protocols hosted on protocols.io. Read more information on sharing protocols at https://plos.org/protocols?utm_medium=editorial-email&utm_source=authorletters&utm_campaign=protocols.

As the corresponding author, your ORCID iD is verified in the submission system and will appear in the published article. PLOS supports the use of ORCID, and we encourage all coauthors to register for an ORCID iD and use it as well. Please encourage your coauthors to verify their ORCID iD within the submission system before final acceptance, as unverified ORCID iDs will not appear in the published article. *Only*  the individual author can complete the verification step; PLOS staff *cannot*  verify ORCID iDs on behalf of authors.

We look forward to receiving your revised manuscript.

Kind regards,

Alexander A. Mongin, Ph.D.

Academic Editor

PLOS One

Journal Requirements:

If the reviewer comments include a recommendation to cite specific previously published works, please review and evaluate these publications to determine whether they are relevant and should be cited. There is no requirement to cite these works unless the editor has indicated otherwise.

[Note: HTML markup is below. Please do not edit.]

Reviewers' comments:

Reviewer's Responses to Questions

**Comments to the Author**

1. If the authors have adequately addressed your comments raised in a previous round of review and you feel that this manuscript is now acceptable for publication, you may indicate that here to bypass the “Comments to the Author” section, enter your conflict of interest statement in the “Confidential to Editor” section, and submit your "Accept" recommendation.

Reviewer #1: (No Response)

Reviewer #2: (No Response)

**********

2. Is the manuscript technically sound, and do the data support the conclusions?

The manuscript must describe a technically sound piece of scientific research with data that supports the conclusions. Experiments must have been conducted rigorously, with appropriate controls, replication, and sample sizes. The conclusions must be drawn appropriately based on the data presented.

Reviewer #1: Yes

Reviewer #2: Partly

**********

3. Has the statistical analysis been performed appropriately and rigorously?

Reviewer #1: Yes

Reviewer #2: (No Response)

**********

4. Have the authors made all data underlying the findings in their manuscript fully available?

The PLOS Data policy requires authors to make all data underlying the findings described in their manuscript fully available without restriction, with rare exception (please refer to the Data Availability Statement in the manuscript PDF file). The data should be provided as part of the manuscript or its supporting information, or deposited to a public repository. For example, in addition to summary statistics, the data points behind means, medians and variance measures should be available. If there are restrictions on publicly sharing data—e.g. participant privacy or use of data from a third party—those must be specified.

Reviewer #1: Yes

Reviewer #2: Yes

**********

5. Is the manuscript presented in an intelligible fashion and written in standard English?

PLOS ONE does not copyedit accepted manuscripts, so the language in submitted articles must be clear, correct, and unambiguous. Any typographical or grammatical errors should be corrected at revision, so please note any specific errors here.

Reviewer #1: Yes

Reviewer #2: Yes

**********

6. Review Comments to the Author

Please use the space provided to explain your answers to the questions above. You may also include additional comments for the author, including concerns about dual publication, research ethics, or publication ethics. (Please upload your review as an attachment if it exceeds 20,000 characters)

Reviewer #1: The authors employ an astrocytic cell line and an in vitro model of ischemia–reperfusion, combined with pharmacological interventions, to demonstrate that glibenclamide-sensitive SUR1-TRPM4 channels are not only involved in astrocytic swelling but also contribute to ischemia–reperfusion-induced ROS production, ER stress, and inflammatory responses.

Overall, the manuscript is clearly written and well organized. The experimental design is described in sufficient detail, and the results are presented and discussed in a comprehensive manner. The data appear robust and convincing, and the experiments were conducted rigorously, with appropriate replication across treatment conditions.

Major Comments

Figure quality

The quality of the figures is currently insufficient for publication. Several images are of low resolution, and some labels are difficult to read. All figures should be replaced with high-resolution versions to ensure clarity and readability.

Redundancy of astrocyte swelling experiments

The role of glibenclamide in reducing astrocyte swelling via SUR1-TRPM4 channels has already been well established in the literature. Repeating these findings may not add substantial novelty. The authors may consider reducing this section and instead placing greater emphasis on the less well-characterized downstream effects.

Lack of mechanistic insight into downstream signaling

While the study convincingly demonstrates downstream effects such as ROS production, ER stress, and inflammation, the underlying signaling mechanisms remain unclear and are not addressed in the manuscript. For example, it would be important to consider whether these effects are mediated by changes in intracellular calcium levels. Additionally, the factors responsible for the effects of the conditioned media are not identified. Including experiments to explore these mechanisms would significantly strengthen the manuscript and provide valuable mechanistic insight.

Minor Comments

Therapeutic context of glibenclamide

In the final part of the Introduction, the authors suggest that glibenclamide may have therapeutic potential. However, this concept has already been proposed in earlier studies, and the compound has been under preclinical investigation for brain ischemia for some time. The text should be revised to better reflect the current state of the field.

Figure 8 – pSTAT3 interpretation

Figure 8 does not fully reflect the described results. Specifically, pSTAT3 levels appear to be increased not only in cells treated with conditioned media but also - more prominently - in ischemic astrocytes themselves. This aspect should be clearly represented in the figure and/or clarified in the figure legend.

Reviewer #2: 1. This m/s is not particularly novel, as earlier studies have shown similar results. As the authors know, Simard’s group has published work identical to the current findings (e.g., SUR1-TRPM4 channel activation and phasic secretion of MMP-9 induced by tPA in brain endothelial cells, Volodymyr Gerzanich,….J. Marc Simard; Cell swelling and a nonselective cation channel regulated by internal Ca2+ and ATP in native reactive astrocytes from adult rat brain. JNeurosci 21: 6512–6521), and the authors cited some of them, except for the pharmacological manipulation.

2. The cell line used in this study is unacceptable. The rat astrocyte cell line CTX-TNA2 (ATCC® CRL-2006™) is an immortalized, type 1 astrocyte line derived from the cortex of 1-day-old Sprague-Dawley rats. These adherent cells, often used, exhibit a fibroblast-like morphology. More importantly, only 20% of these cells exhibit glial fibrillary acidic protein (GFAP) immunoreactivity. Thus, this cell line can't be used for any GFAP-related applications. As the authors know, these cells often lack specific functions of normal cells, such as metabolic pathways or tissue-specific markers, due to changes in gene expression and metabolic rearrangement. Since these cells are derived from tumors, the results should have been validated in primary cells to ensure physiological relevance.

3. The argument that inhibition of SUR1–TRPM4 attenuates astrocyte swelling in injury-like settings, and the strongest evidence is in activated astrocytes and post-ischemic brain tissue rather than in a pure OGD-only astrocyte culture model, is hard to accept. Because the in vitro system lacks blood components. This needs to be reversed.

4. The manuscript is poorly written and lacks methodological details. For example, the authors noted that they used 96-well plates. If that’s the case, how many plates were used per condition? TomoDish imaging dishes, what does n=4 mean?

5. The major issues when using Astrocyte-conditioned medium (ACM) with naive cells are reproducibility and a lack of standardization in its secretome composition. Because ACM is produced by cultured astrocytes, which often default to a reactive or immature phenotype in 2/3D systems, the precise cocktail of secreted proteins, lipids, and cytokines can vary significantly depending on astrocyte culture conditions, passage number, and basal media. It's unclear how the authors address these issues.

6. The authors used a microplate reader for ROS measurement. But flow cytometry is a reliable tool for measuring ROS using the Invitrogen Total Reactive Oxygen Species (ROS) Assay Kit (Cat. No. 88-5930-74). Because the fluorescent signal will now be retained after cell permeabilization.

7. Provide the original number for Fig. 1A instead of expressing it as a percentage.

8. It is difficult to compare legends with figures because the legends are far from the figures. This may be due to the journal's style (the journal should look into it).

9. The fluorescence images are of poor quality (Fig 7 specifically). The astrocytes may have been stained with May-Grunwald to better assess morphology or density. Because we don’t know what cells we are looking at.

10. It's unclear what concentrations of Gli were used (it should be explicitly mentioned in all figure legends).

11. Fig. 9 needs to be redrawn. Astrocyte death and reactivity can occur under the same conditions, particularly in severe neurodegenerative diseases, stroke, and traumatic brain injuries. While reactive astrogliosis is traditionally viewed as a protective, hypertrophy-driven (growth) response to damage, it often transitions into a pathological state in which reactive astrocytes lose their supportive functions, become neurotoxic, and eventually die. This raises the question: Did the authors measure the neurotoxic substances associated with this condition? If so, what are they? Since this is an in vitro condition, what may have occurred in vivo?

**********

7. PLOS authors have the option to publish the peer review history of their article (what does this mean?). If published, this will include your full peer review and any attached files.

If you choose “no”, your identity will remain anonymous but your review may still be made public.

**Do you want your identity to be public for this peer review?**  For information about this choice, including consent withdrawal, please see our Privacy Policy.

Reviewer #1: **Yes:**  Helena Pivonkova

Reviewer #2: **Yes:**  Arumugam R. Jayakumar

[NOTE: If reviewer comments were submitted as an attachment file, they will be attached to this email and accessible via the submission site. Please log into your account, locate the manuscript record, and check for the action link "View Attachments". If this link does not appear, there are no attachment files.]

To ensure your figures meet our technical requirements, please review our figure guidelines: https://journals.plos.org/plosone/s/figures

You may also use PLOS’s free figure tool, NAAS, to help you prepare publication quality figures: https://journals.plos.org/plosone/s/figures#loc-tools-for-figure-preparation.

NAAS will assess whether your figures meet our technical requirements by comparing each figure against our figure specifications.

# Author comment: Author response to Decision Letter 2

**DOI:** 10.1371/journal.pone.0352151.r004

22 May 2026

[1] Both reviewers expressed concerns about novelty. These concerns do not need to be addressed because PLOS ONE does not require novelty or major conceptual breakthroughs for publication, but instead focuses on methodological rigor and the soundness of the conclusions. However, all other issues raised by the reviewers should be specifically addressed in detailed point-by-point responses.

Author response:

We appreciate the clarification regarding novelty criteria at PLOS ONE. Despite the SUR1-TRPM4 is not a novel participant in astroglial injury, this study has further evaluated some particular molecular pathways involved in the development of the injury. These knowledges might contribute to further explanation of the full picture of the injury (Line 27-29, 93-94,632-634).

[2] Reviewer 2 raised a valid concern regarding the limitations of using an immortalized cell line in place of primary astrocytes. This limitation should be explicitly acknowledged in the manuscript text. I would also advise against referring to CTX-TNA2 cells as “astrocytes”; terms such as “CTX-TNA2 cells” or “astroglial cell line” would be more appropriate.

Author response:

Thank you very much for the comment. In response to Reviewer 2’s concern regarding the use of an immortalized cell line, we have explicitly acknowledged this limitation in the Discussion section (Line 436-437, 452-458). We also revised the terminology throughout the manuscript to avoid referring to CTX-TNA2 cells as “astrocytes,” and instead use terms such as “CTX-TNA2 cells” where appropriate.

[3] Reviewer 2 also cautioned against excessive generalization of the in vitro OGD findings to astrocyte pathology in situ. You may speculate about the potential significance and applicability of your findings, but statements such as (lines 459–461) “Our findings further indicate that SUR1–TRPM4 is involved in both astrocyte swelling and astrocyte reactivity following ischemic injury” should be avoided or substantially softened.

Author response:

Thank you for the comment, we agree that the in vitro OGD might not totally represent the in vivo condition and the conclusion should be carefully stated. So, we revised the statements that may overgeneralize the in vitro OGD findings to astrocyte pathology in situ. However, we believe that this in vitro model can represent some of the processes that occur in vivo. We also emphasized this limitation in the Discussion section (Line 459-465, 514-516, 537-538, 562, 595, 603-604, 617-620, 630-631).

[4] Please address the Editor’s concerns regarding statistical analyses. Most journals have now transitioned to reporting SD rather than SEM. In all cases where control data were normalized to 100% or unity (e.g., Fig. 1A, Fig. 3C, Fig. 4A,B, Fig. 6B–D), one-way ANOVA is not an appropriate statistical test. Instead, you may use one-sample t-tests with Bonferroni correction for multiple comparisons or consult a statistician regarding alternative approaches.

Author response:

Thank you very much for the comment. We addressed the concerns regarding statistical analyses by revising the statistical methods and reanalyzing the relevant datasets. SD is now reported instead of SEM throughout the manuscript. For datasets normalized to 100% or unity, we replaced one-way ANOVA with appropriate one-sample statistical analyses with Bonferroni correction, as recommended. The Methods section has also been updated accordingly (Line 2018-216). The figure legends from figure 1 to figure 8 have also been updated accordingly.

[5] Please clarify what is meant by the term “intracellular concentration” as presented in Fig. 2D.

Author response:

Thank you very much for the comment. We clarified the meaning of the term “intracellular concentration” from the Tomocube HT-2H system in the Method section (Line 158-160).

[6] It appears that in the Western blot analyses, β-actin loading controls were probed on separate membranes. If this is the case, it must be clearly stated in the Methods and/or figure legends.

Thank you very much for the comment. We clarify that the β-actin loading controls are from the same membrane as the proteins of interest. However, to increase the clarity of the pictures, the exposure time for the control β-actin bands is shorter than the proteins of interest due to low activity of proteins of interest as shown in the supplement data file. This ratio might not represent the real proteins level but should be possible for comparison among different treatments which are subjected to the same measure. This massage for clarification is added in the Methods and figure legends (Line 207-208).

[7] Both reviewers expressed concerns regarding the resolution and/or composition of the figures. Personally, I found the high-resolution versions generally acceptable (perhaps the reviewers did not access them). Nevertheless, I agree that several figures, particularly Figure 7, will be difficult to read when printed and may even be problematic in the online format. Please consider reorganizing these figures to improve overall legibility.

Thank you very much for the comment. We carefully reviewed and reorganized the all the figures to improve readability and presentation quality, particularly Figure 7. Higher-resolution and revised figure files have been uploaded with the resubmission.

6. Review Comments to the Author

Please use the space provided to explain your answers to the questions above. You may also include additional comments for the author, including concerns about dual publication, research ethics, or publication ethics. (Please upload your review as an attachment if it exceeds 20,000 characters)

Reviewer #1: The authors employ an astrocytic cell line and an in vitro model of ischemia–reperfusion, combined with pharmacological interventions, to demonstrate that glibenclamide-sensitive SUR1-TRPM4 channels are not only involved in astrocytic swelling but also contribute to ischemia–reperfusion-induced ROS production, ER stress, and inflammatory responses.

Overall, the manuscript is clearly written and well organized. The experimental design is described in sufficient detail, and the results are presented and discussed in a comprehensive manner. The data appear robust and convincing, and the experiments were conducted rigorously, with appropriate replication across treatment conditions.

Major Comments

Figure quality

The quality of the figures is currently insufficient for publication. Several images are of low resolution, and some labels are difficult to read. All figures should be replaced with high-resolution versions to ensure clarity and readability.

Author response:

We thank the reviewer for this comment. All figures have been replaced with higher-resolution versions to improve clarity and readability. In particular, Figure 7 has been reorganized and enlarged to improve visualization of fluorescence signals and labeling. We also adjusted font sizes and panel spacing throughout the figures to enhance legibility in both online and print formats.

Redundancy of astrocyte swelling experiments

The role of glibenclamide in reducing astrocyte swelling via SUR1-TRPM4 channels has already been well established in the literature. Repeating these findings may not add substantial novelty. The authors may consider reducing this section and instead placing greater emphasis on the less well-characterized downstream effects.

Author response:

Thank you very much, we appreciate this important suggestion. The role of SUR1-TRPM4 in astrocyte swelling has been published elsewhere. However, our study focused more extensively on the downstream consequences associated with SUR1–TRPM4 activation, including ROS production, ER stress, inflammatory signaling, and conditioned medium effects. We also revised the Discussion to better clarify the rationale and contribution of the present study within the existing literature (Line 450-452,463-466, 606-617). Additional citations are added as appropriate.

Lack of mechanistic insight into downstream signaling

While the study convincingly demonstrates downstream effects such as ROS production, ER stress, and inflammation, the underlying signaling mechanisms remain unclear and are not addressed in the manuscript. For example, it would be important to consider whether these effects are mediated by changes in intracellular calcium levels. Additionally, the factors responsible for the effects of the conditioned media are not identified. Including experiments to explore these mechanisms would significantly strengthen the manuscript and provide valuable mechanistic insight.

Author response:

We thank the reviewer for this thoughtful comment. We agree that intracellular calcium signaling and identification of factors within conditioned media represent important mechanistic questions. However, these investigations were beyond the scope of the current study. We have now explicitly acknowledged these limitations in the Discussion section and added a statement indicating that future studies are needed to investigate calcium-dependent mechanisms and to identify specific mediators present in conditioned media responsible for downstream effects (Line 478-485, 597-602).

Minor Comments

Therapeutic context of glibenclamide

In the final part of the Introduction, the authors suggest that glibenclamide may have therapeutic potential. However, this concept has already been proposed in earlier studies, and the compound has been under preclinical investigation for brain ischemia for some time. The text should be revised to better reflect the current state of the field.

Author response:

We thank the reviewer for this comment. We agree with the reviewer and revised the final section of the Introduction to better reflect the current state of the field (Line 89-94). The revised text now acknowledges prior preclinical investigations of glibenclamide in ischemic brain injury and avoids overstating novelty or therapeutic implications. In the manuscript, where appropriate, we emphasized on other aspect of study beside the already know role of glibenclamide in reduce astrocyte swelling (Line 630-632).

Figure 8 – pSTAT3 interpretation

Figure 8 does not fully reflect the described results. Specifically, pSTAT3 levels appear to be increased not only in cells treated with conditioned media but also - more prominently - in ischemic astrocytes themselves. This aspect should be clearly represented in the figure and/or clarified in the figure legend.

Author response:

We thank the reviewer for this observation. We have revised the description of Figure 8 and clarified in both the Results section (Line 416-425) and figure legend (Line 427-434) that pSTAT3 levels were elevated not only in conditioned medium-treated cells but also in ischemic CTX-TNA2 cells themselves (Figure 6). The revised figure presentation more accurately reflects these findings. These findings are also discussed in the Discussion section (Line 578-582).

Reviewer #2:

1. This m/s is not particularly novel, as earlier studies have shown similar results. As the authors know, Simard’s group has published work identical to the current findings (e.g., SUR1-TRPM4 channel activation and phasic secretion of MMP-9 induced by tPA in brain endothelial cells, Volodymyr Gerzanich,….J. Marc Simard; Cell swelling and a nonselective cation channel regulated by internal Ca2+ and ATP in native reactive astrocytes from adult rat brain. JNeurosci 21: 6512–6521), and the authors cited some of them, except for the pharmacological manipulation.

Author response:

We appreciate the reviewer’s comments, we revised the manuscript to better position our findings within the existing literature and clarified the aspects of downstream cellular responses examined in the present study. We also reviewed the suggested references and incorporated relevant citations where appropriate (Line 27-29, 93-94,632-634).

2. The cell line used in this study is unacceptable. The rat astrocyte cell line CTX-TNA2 (ATCC® CRL-2006™) is an immortalized, type 1 astrocyte line derived from the cortex of 1-day-old Sprague-Dawley rats. These adherent cells, often used, exhibit a fibroblast-like morphology. More importantly, only 20% of these cells exhibit glial fibrillary acidic protein (GFAP) immunoreactivity. Thus, this cell line can't be used for any GFAP-related applications. As the authors know, these cells often lack specific functions of normal cells, such as metabolic pathways or tissue-specific markers, due to changes in gene expression and metabolic rearrangement. Since these cells are derived from tumors, the results should have been validated in primary cells to ensure physiological relevance.

Author response:

We thank the reviewer for this important concern. We acknowledge that CTX-TNA2 is an immortalized astroglial cell line with known limitations compared with primary astrocytes. Accordingly, we have explicitly discussed these limitations in the revised Discussion section(Line 453-459). In addition, throughout the manuscript, we replaced the term “astrocytes” with “CTX-TNA2 cells” where appropriate to avoid overinterpretation of the cellular phenotype. We agree that validation in primary astrocytes would further strengthen physiological relevance and have acknowledged this as an important direction for future studies (Line 635-637).

3. The argument that inhibition of SUR1–TRPM4 attenuates astrocyte swelling in injury-like settings, and the strongest evidence is in activated astrocytes and post-ischemic brain tissue rather than in a pure OGD-only astrocyte culture model, is hard to accept. Because the in vitro system lacks blood components. This needs to be reversed.

Author response:

We appreciate the reviewer’s concern regarding extrapolation of in vitro OGD findings to in vivo conditions. We have substantially softened statements that generalized our findings to astrocyte pathology in situ and clarified that the current conclusions are limited to the in vitro OGD/reoxygenation model employed in this study. We also acknowledged that the absence of blood-derived factors and multicellular interactions represents an important limitation of the model system (Line 460-466).

4. The manuscript is poorly written and lacks methodological details. For example, the authors noted that they used 96-well plates. If that’s the case, how many plates were used per condition? TomoDish imaging dishes, what does n=4 mean?

Author response:

We thank the reviewer for highlighting this issue. Additional methodological details have now been added throughout the Methods section (Line 128-137, 141-142, 164-165), including the number of wells, plates, biological replicates, and clarification of sample sizes (n values) for each experiment. We also clarified the meaning of “n=4” for imaging experiments performed using TomoDish imaging dishes (Line 266-267).

5. The major issues when using Astrocyte-conditioned medium (ACM) with naive cells are reproducibility and a lack of standardization in its secretome composition. Because ACM is produced by cultured astrocytes, which often default to a reactive or immature phenotype in 2/3D systems, the precise cocktail of secreted proteins, lipids, and cytokines can vary significantly depending on astrocyte culture conditions, passage number, and basal media. It's unclear how the authors address these issues.

Author response:

We appreciate this important point. We have now added additional methodological details regarding ACM preparation, including cell passage conditions, culture conditions, timing of collection, and normalization procedures used to improve reproducibility. We also acknowledged in the Discussion that variability in conditioned media composition is an inherent limitation of this experimental approach(Line 578-582, 597-602).

6. The authors used a microplate reader for ROS measurement. But flow cytometry is a reliable tool for measuring ROS using the Invitrogen Total Reactive Oxygen Species (ROS) Assay Kit (Cat. No. 88-5930-74). Because the fluorescent signal will now be retained after cell permeabilization.

Author response:

We thank the reviewer for this valuable suggestion. We agree that flow cytometry is a sensitive and widely used method for ROS analysis. In the present study, ROS levels were

Attachment Submitted filename: Response to Reviewers.docx

# Peer review: Decision Letter 2

**DOI:** 10.1371/journal.pone.0352151.r005

**Authors:** Alexander Mongin, Alexander Mongin, Alexander Mongin

8 Jun 2026

Inhibition of SUR1-TRPM4 attenuates astrocyte swelling and reactivity under oxygen-glucose deprivation/reoxygenation

PONE-D-26-12414R2

Dear Dr. Tapechum,

We’re pleased to inform you that your manuscript has been judged scientifically suitable for publication and will be formally accepted for publication once it meets all outstanding technical requirements.

Within one week, you’ll receive an e-mail detailing the required amendments. When these have been addressed, you’ll receive a formal acceptance letter and your manuscript will be scheduled for publication.

An invoice will be generated when your article is formally accepted. Please note, if your institution has a publishing partnership with PLOS and your article meets the relevant criteria, all or part of your publication costs will be covered. Please make sure your user information is up-to-date by logging into Editorial Manager at Editorial Manager® and clicking the ‘Update My Information' link at the top of the page. For questions related to billing, please contact billing support.

If your institution or institutions have a press office, please notify them about your upcoming paper to help maximize its impact. If they’ll be preparing press materials, please inform our press team as soon as possible -- no later than 48 hours after receiving the formal acceptance. Your manuscript will remain under strict press embargo until 2 pm Eastern Time on the date of publication. For more information, please contact onepress@plos.org.

Kind regards,

Alexander A. Mongin, Ph.D.

Academic Editor

PLOS One

Additional Editor Comments (optional):

Reviewers' comments:

Reviewer's Responses to Questions

**Comments to the Author**

1. If the authors have adequately addressed your comments raised in a previous round of review and you feel that this manuscript is now acceptable for publication, you may indicate that here to bypass the “Comments to the Author” section, enter your conflict of interest statement in the “Confidential to Editor” section, and submit your "Accept" recommendation.

Reviewer #1: All comments have been addressed

Reviewer #2: All comments have been addressed

**********

2. Is the manuscript technically sound, and do the data support the conclusions?

The manuscript must describe a technically sound piece of scientific research with data that supports the conclusions. Experiments must have been conducted rigorously, with appropriate controls, replication, and sample sizes. The conclusions must be drawn appropriately based on the data presented.

Reviewer #1: Yes

Reviewer #2: Yes

**********

3. Has the statistical analysis been performed appropriately and rigorously?

Reviewer #1: Yes

Reviewer #2: Yes

**********

4. Have the authors made all data underlying the findings in their manuscript fully available?

The PLOS Data policy requires authors to make all data underlying the findings described in their manuscript fully available without restriction, with rare exception (please refer to the Data Availability Statement in the manuscript PDF file). The data should be provided as part of the manuscript or its supporting information, or deposited to a public repository. For example, in addition to summary statistics, the data points behind means, medians and variance measures should be available. If there are restrictions on publicly sharing data—e.g. participant privacy or use of data from a third party—those must be specified.

Reviewer #1: Yes

Reviewer #2: Yes

**********

5. Is the manuscript presented in an intelligible fashion and written in standard English?

PLOS ONE does not copyedit accepted manuscripts, so the language in submitted articles must be clear, correct, and unambiguous. Any typographical or grammatical errors should be corrected at revision, so please note any specific errors here.

Reviewer #1: Yes

Reviewer #2: Yes

**********

6. Review Comments to the Author

Please use the space provided to explain your answers to the questions above. You may also include additional comments for the author, including concerns about dual publication, research ethics, or publication ethics. (Please upload your review as an attachment if it exceeds 20,000 characters)

Reviewer #1: (No Response)

Reviewer #2: The authors made significant improvements to their initial submission. My concerns were addressed appropriately.

**********

7. PLOS authors have the option to publish the peer review history of their article (what does this mean?). If published, this will include your full peer review and any attached files.

If you choose “no”, your identity will remain anonymous but your review may still be made public.

**Do you want your identity to be public for this peer review?**  For information about this choice, including consent withdrawal, please see our Privacy Policy.

Reviewer #1: No

Reviewer #2: **Yes:**  Arumugam Radhakrishnan Jayakumar

**********

# Editor report: Acceptance letter

**DOI:** 10.1371/journal.pone.0352151.r006

**Authors:** Alexander Mongin, Alexander Mongin, Alexander Mongin

PONE-D-26-12414R2

PLOS One

Dear Dr. Tapechum,

I'm pleased to inform you that your manuscript has been deemed suitable for publication in PLOS One. Congratulations! Your manuscript is now being handed over to our production team.

At this stage, our production department will prepare your paper for publication. This includes ensuring the following:

* All references, tables, and figures are properly cited

* All relevant supporting information is included in the manuscript submission,

* There are no issues that prevent the paper from being properly typeset

You will receive further instructions from the production team, including instructions on how to review your proof when it is ready. Please keep in mind that we are working through a large volume of accepted articles, so please give us a few days to review your paper and let you know the next and final steps.

Lastly, if your institution or institutions have a press office, please let them know about your upcoming paper now to help maximize its impact. If they'll be preparing press materials, please inform our press team within the next 48 hours. Your manuscript will remain under strict press embargo until 2 pm Eastern Time on the date of publication. For more information, please contact onepress@plos.org.

You will receive an invoice from PLOS for your publication fee after your manuscript has reached the completed accept phase. If you receive an email requesting payment before acceptance or for any other service, this may be a phishing scheme. Learn how to identify phishing emails and protect your accounts at https://explore.plos.org/phishing.

If we can help with anything else, please email us at customercare@plos.org.

Thank you for submitting your work to PLOS ONE and supporting open access.

Kind regards,

PLOS ONE Editorial Office Staff

on behalf of

Dr. Alexander A. Mongin

Academic Editor

PLOS One
